# Designing a bed-side system for predicting length of stay in a neonatal intensive care unit

**DOI:** 10.1038/s41598-021-82957-z

**Published:** 2021-02-08

**Authors:** Harpreet Singh, Su Jin Cho, Shubham Gupta, Ravneet Kaur, S. Sunidhi, Satish Saluja, Ashish Kumar Pandey, Mihoko V. Bennett, Henry C. Lee, Ritu Das, Jonathan Palma, Ryan M. McAdams, Avneet Kaur, Gautam Yadav, Yao Sun

**Affiliations:** 1 Child Health Imprints (CHIL) Pte. Ltd, Singapore, Singapore; 2 Department of Pediatrics, Ewha Womans University School of Medicine, Seoul, Korea; 3 Department of Neonatology, Sir Ganga Ram Hospital, New Delhi, India; 4 Department of Mathematics, Indraprastha Institute of Information Technology, New Delhi, India; 5 Division of Neonatal and Developmental Medicine, Department of Pediatrics, Stanford University, Stanford, CA USA; 6 California Perinatal Quality Care Collaborative, Stanford, CA USA; 7 Department of Pediatrics, University of Wisconsin School of Medicine and Public Health, Madison, USA; 8 Department of Neonatology, Apollo Cradle Hospitals, New Delhi, India; 9 Department of Pediatrics, Kalawati Hospital, Rewari, India; 10 University of California, San Francisco, USA

**Keywords:** Biotechnology, Computational biology and bioinformatics, Health care, Medical research

## Abstract

Increased length of stay (LOS) in intensive care units is directly associated with the financial burden, anxiety, and increased mortality risks. In the current study, we have incorporated the association of day-to-day nutrition and medication data of the patient during its stay in hospital with its predicted LOS. To demonstrate the same, we developed a model to predict the LOS using risk factors (a) perinatal and antenatal details, (b) deviation of nutrition and medication dosage from guidelines, and (c) clinical diagnoses encountered during NICU stay. Data of 836 patient records (12 months) from two NICU sites were used and validated on 211 patient records (4 months). A bedside user interface integrated with EMR has been designed to display the model performance results on the validation dataset. The study shows that each gestation age group of patients has unique and independent risk factors associated with the LOS. The gestation is a significant risk factor for neonates < 34 weeks, nutrition deviation for < 32 weeks, and clinical diagnosis (sepsis) for ≥ 32 weeks. Patients on medications had considerable extra LOS for ≥ 32 weeks’ gestation. The presented LOS model is tailored for each patient, and deviations from the recommended nutrition and medication guidelines were significantly associated with the predicted LOS.

## Introduction

Increased length of stay (LOS) in hospital critical care units (CCU) has been associated with adverse events, increased costs, and increased risks of mortality^1^. Studies have explored LOS prediction and its relationship with institutional, clinical, social, and psychological factors^2,3^. Institutional factors such as CCU geographic location, resources, organizational structure, and leadership affects both length of stay and patient care^4,5^. Clinical factor-based prediction studies have highlighted the relationship of LOS with different clinical diagnoses and have used different severity scores, including the Acute Physiology and Chronic Health Evaluation (APACHE), Simplified Acute Physiology Score (SAPS), and Mortality Probability Model (MPM)^6^. Social and physiological studies have explored insurance data and focused on cost-saving by involving LOS prediction in prevention programs^4^. All these efforts have led to the increasing use of mathematical models to analyze LOS to decrease cost and reduce the risk of adverse events in clinical care^5,7^.

The availability and analysis of Electronic Health Records (EHR) data have further enhanced the analysis of factors affecting LOS^8^. Recent studies have shown that predictive EHR modeling using neural network-based artificial intelligence can aid in decision-making related to patient outcomes with respect to various morbidities, types of interventions, and LOS^9,10^. CCUs have used evidence-based interventions, including standardized procedures and treatment protocols, to improve clinical outcomes and shorten LOS^11,12^. CCU's achieve care standardization by following established nutrition and medication protocols for different age groups. These include nutrition recommendations from the American Society for Parenteral and Enteral Nutrition (ASPEN)^13^ or the European Society for Pediatric Gastroenterology Hepatology and Nutrition (ESPGHAN)^14^. Similarly, medications are administered as per NEOFAX^15^, Lexicomp^16^, or local pharmacopeia guidelines.

The aforementioned approaches are often burdensome for clinical staffs that care for patients in CCUs, especially in settings like neonatal intensive care units (NICUs). The health status of critically ill and premature neonates is dynamic, and variables, such as weight, can change on a daily basis, which makes decision-making more challenging. Given the extreme fragility of these sick neonates, deviations in the prescribed nutrition or medications may have adverse effects on neonate’s health and LOS^17,18^. Various studies have reported nutrition and medication from the hospital protocols. Ana et al., studied the prescription of parenteral nutrition in preterm infants^19^. They evaluated the nutrition compliance with the hospital’s protocol and with the guidelines of American Society for Parenteral and Enteral Nutrition (ASPEN), European Society for Clinical Nutrition and Metabolism (ESPEN), and Spanish Society of Clinical Nutrition and Metabolism (SENPE). The differences in macronutrient intake and the total duration of parenteral nutrition were analyzed according to gestational age and birth weight. Eslami et al., reported types and frequency of medication errors in the NICUs, they considered prescription (dosage error to a deviation of ≥ 10% from the references) and administration errors in study^20^. Cimino et al., developed a matrix for determining the predominant type, cause category, and rate of medication prescribing errors, and explored the effectiveness of hospital-based improvement initiatives among pediatric intensive care units^21^.

Unintended nutritional or medication deviations can have short-term and long-term effects on the neonate, including worse neurodevelopmental outcomes and higher risks of chronic diseases^22,23^. The current set of LOS predictors in CCU settings fails to adapt to changing condition of neonates across gestation categories and the deviation of dosage with respect to nutrition and medication.

## Objective

In the current study, we present a LOS model that incorporates independent variables, referred to as *risk factors,* based on gestational ages in neonatal population. These risk factors represent patient-specific aspects of the CCU clinical course, including (a) antenatal and perinatal factors, (b) nutritional orders, (c) medication orders, and (d) clinical diagnosis data. The study aim is to develop a model for LOS prediction for each gestation category of a neonate using most associated independent variables. The study dataset was divided into “testing” and “validation” sets. The model was trained on the testing dataset, and the coefficients indicating the association of risk factors in predicting LOS was calculated. The performance of the generated model was then assessed on the validation dataset. The study also presents a bedside EMR integrated interface to visualize these results or coefficients of associated risk factors for each gestation category.

The scope of current work is limited to the prediction of LOS and its use for patient counseling in NICU settings. In the current form, it does not recommend clinicians to modify the nutrition and medication orders. At admission, the clinician can use the predicted value to counsel the family about the patient’s LOS. Whereas, during the hospital stay, the interface will provide an update to the healthcare staff of any change observed in the predicted LOS due to administered nutrition and medication to the neonate.

## Data and methods

### Setting and study population

**Figure 1:**
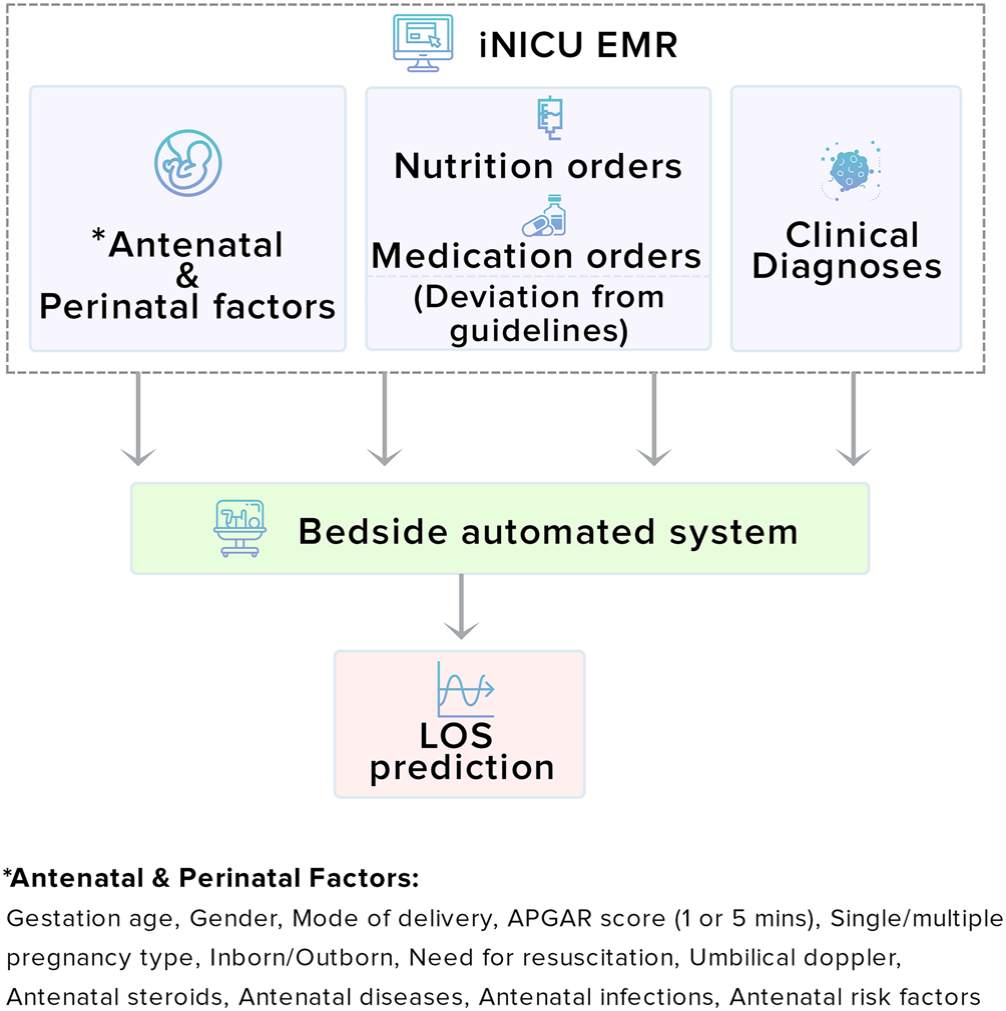
Overview of bedside automated system for predicting length of stay.

Digital data was collected for neonates admitted to two NICUs study sites over a 16 month (July 2018—November 2019) duration^24^. The study sites included a 22-beds urban (Apollo Cradle Moti Nagar, New Delhi) and 17 beds rural (Kalawati Hospital and Kamla Nursing Home, Rewari), level III NICU in India. The urban NICU consists of three neonatologists that have a doctorate in neonatal sciences. The rural NICU consists of three neonatologist that have doctorate in neonatal sciences along with, four residents and 18 nurses. The Institutional Review Board of both NICU’ approved the study with a waiver of informed consent. All electronic health records were de-identified (in accordance with HIPAA), and all the research was performed according to relevant guidelines. All neonates who stayed in the NICU for > 24 h and had nutrition orders were eligible for the study. Exclusion criteria included congenital anomalies, palliative care, and discharge on request, transfer, and death cases. Data obtained during the neonate’s NICU stay was segregated into three different risk factor categories, as shown in Fig. 1. These categories were (a) Antenatal and Perinatal factors, (b) Nutrition orders and Medication orders, and (c) Clinical diagnosis. These data were utilized to predict LOS and associated weights of risk factors displayed on the bedside tablet interface.

### Data collection and study design

De-identified individual patient admission-to-discharge data was electronically recorded using the iNICU platform^24^. The data was entered on bedside tablets through an iPAD Pro (12.9 inches, IInd generation) using a Chrome browser, and data was stored in the Postgres SQL database. The clinical diagnosis was marked by consulting neonatologist using International Classification Diseases (ICD) ninth revision during daily rounds (morning, afternoon, and evening) performed at the patient bedside.

The data extraction process extracted the information for each patient from the database and aggregated the same with assessment, medication, and nutrition entries. This step was performed in Java, and it generated a CSV file as an output. The missing data during the study was handled using a four-way approach. (1) System validations: the platform ensured mandatory data entry validations for perinatal and antenatal data such as gestation, APGAR, maternal risk factors, were enforced and notifications were sent to staff in case of missing data. The platform also implemented the medication guideline for the drugs present in the NeoFax system to recommend the correct dosage and frequency of the prescribed medications. (2) Review meetings: regular review meetings with departmental staff ensured the completeness and quality of the entered data in the iNICU platform. (3) Forward filling data: the missing nutrition orders were forward filled from previous order till the next order, there was no change in prescribed enteral or parenteral volume, (4) Imputation strategies: some data for out-patients were still missing during the data analysis process which was handled by data imputation strategy of filling with population mean. Any field containing more than 10% imputed data was not considered for the LOS prediction model.

**Figure 2:**
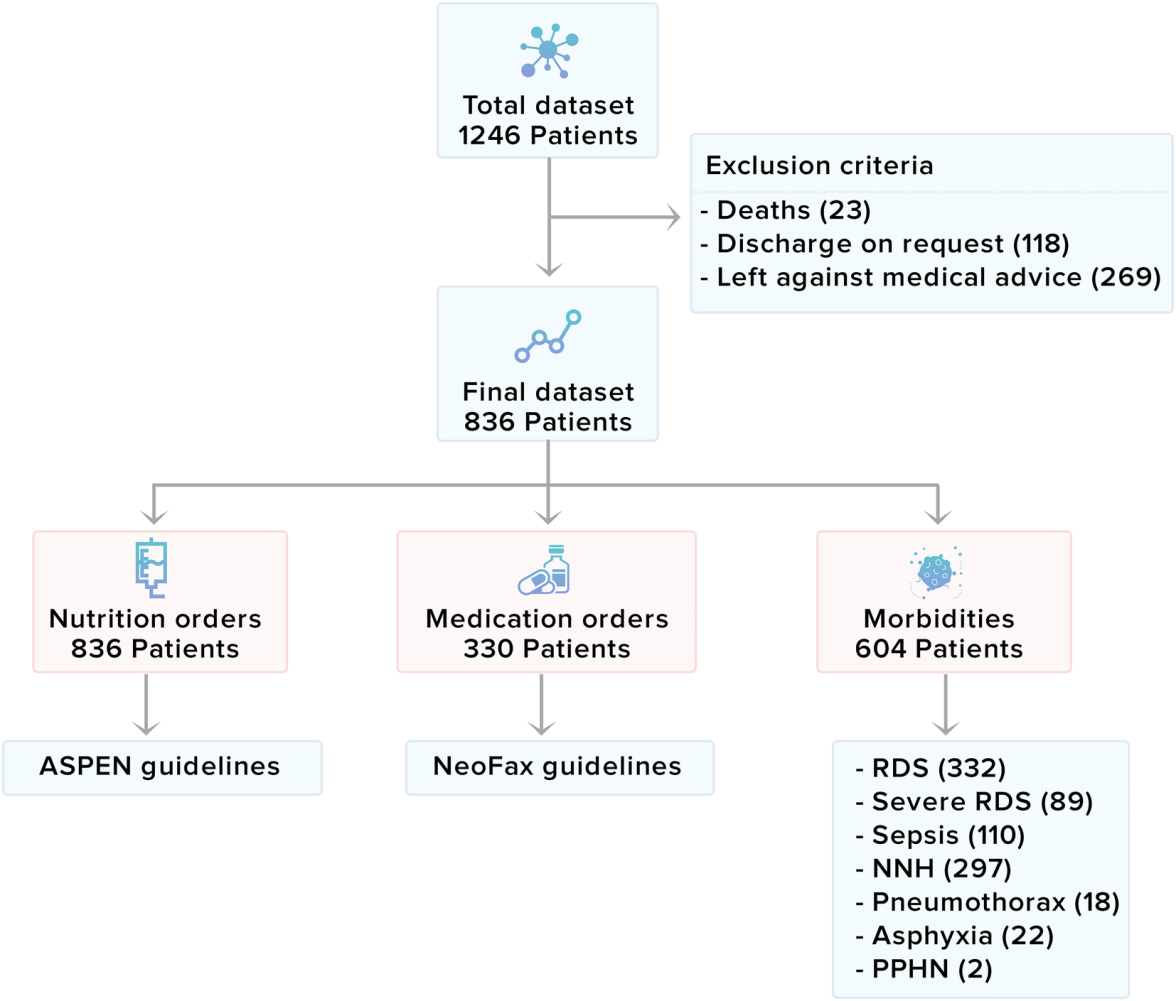
Study design for predicting length of stay using nutrition orders, medication orders, and incidence of clinical diagnosis during stay in NICU.

Patients were randomly assigned a unique identifier, and a look-up-key was not retained, which prevented anonymized data from being linked back to the original, identifiable data. All hospital and ICU identifiers were removed to protect the privacy of contributing institutions and providers. The prospective observational study design, which includes nutrition, medication, and clinical diagnosis data in LOS prediction, is demonstrated in Fig. 2.

### Risk factors for LOS

#### Antenatal and perinatal factors

The admission factors included information such as mode of delivery, pregnancy type (single or multiple), gender, inborn/outborn, gestational age, need for resuscitation, birth weight, antenatal steroids complete vs. incomplete administration (complete for dexamethasone was considered four doses and betamethasone was considered two dosages), antenatal diseases (Hypertension, Gestational Hypertension, Diabetes, Gestational Diabetes Mellitus, Chronic Kidney Disease, Hypothyroidism, Hyperthyroidism, and Miscellaneous), antenatal infections, and antenatal risk factors. The need for resuscitation was defined based on oxygen supplementation, positive pressure ventilation, or administration of chest compressions.

#### Nutrition deviation from ASPEN

**Figure 3:**
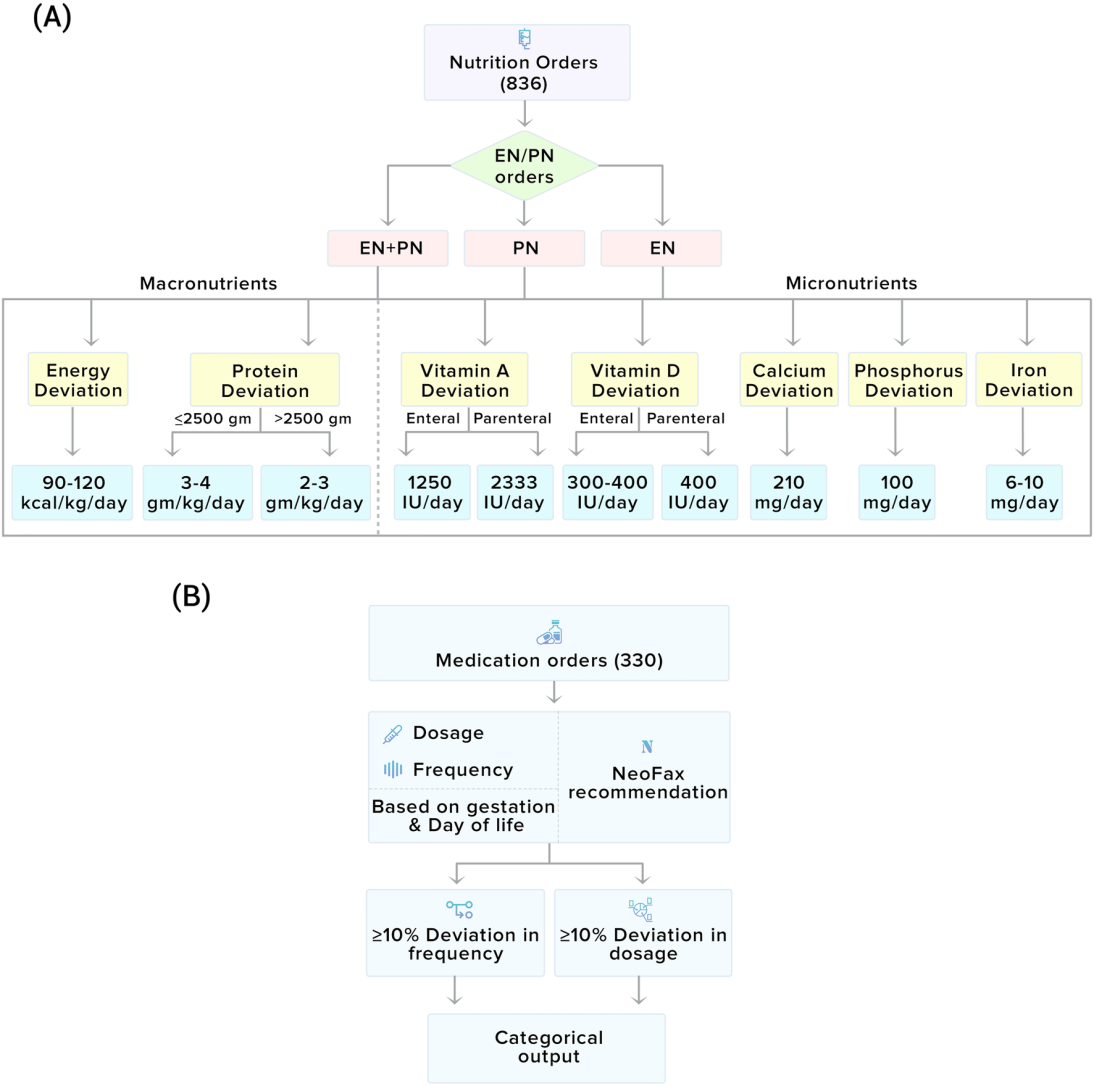
(**A**) Nutrition recommendation for neonates as per ASPEN guidelines. (**B**) Medication deviation reference and calculation pipeline.

ASPEN nutrition guidelines were followed during the study as per the gestational age to calculate the deviation in nutrition orders. Nutrition deviation was defined as any aberration observed during the process of ordering or administering enteral (EN) or parenteral nutrition (PN), categorized as discrepancies in prescribed doctor’s order(s) in comparison with recommended ASPEN guidelines^13^ while administering EN/PN volumes. Integration of nutrition guidelines such as ASPEN with Clinical Decision Support (CDS) has improved the compliance of prescribed dosage on a daily basis in regards to recommended based on gestation, birth weight, and day of life and reduces the error due to process variations amongst NICU^25,26^. Thereafter, various studies have used these deviations and related them in quality initiatives to improve nutrition adequacy or improve delivery of macronutrients^19,27^. In the NICU, there are three possible feeding scenarios for neonates (see Fig. 3A) (a) EN feeds only, (b) only PN, and (c) both EN feeds and PN.

The nutrition deviation calculation was done for macronutrients (i.e., energy and protein deviations) and micro-nutrients (Vitamin A, Vitamin D, Calcium, Phosphorus, and Iron). The deviation in protein orders was calculated as per guideline with neonates with birth weights of ≤ 2500 g and > 2500 g^13^. In cases where neonates received both EN and PN, micro-nutrients were considered based on the highest EN or PN category value.

In a CCU, there can be multiple nutrition orders in a given day based on patient severity; therefore the model calculations are redone daily (for each order) based on captured data. In cases where patient nutrition was withheld during episodes of feed intolerance or NEC (necrotizing enterocolitis), the nutrition deviation from parenteral mode was calculated as per guidelines for PN. If the baby was on both enteral (EN) and parenteral (PN) nutrition modes, the deviation was calculated as per the highest intake recommendation^25,28,29^.

#### Medication deviation from NeoFax

The medicines listed in Table S4a that were used to treat neonatal morbidities were included in the analysis. The NeoFax guidelines were followed during the study to calculate the deviation in medication orders. A medication deviation was defined as any aberration of ≥ 10% in medication dosage or frequency of the recommended value as per NeoFax guidelines^30–32^. For each neonate, medication deviation days were calculated and reported along with LOS days. In the current study, all the medical deviations were counted equal, irrespective of their severity. Figure 3B shows the medication deviation pipeline flow chart for neonates based on each neonate’s dosage and frequency of medication.

The categorical output is shown in Fig. 3B denotes the intermediate result of the decision point, comparing the prescribed medicine amount with recommended NeoFax dosage. This intermediate result, as a Boolean decision, was then inserted into the model to predict LOS based on lsmean.

#### Clinical diagnosis

Clinical diagnosis data included information on most frequent cases recorded in NICUs are: (a) hyperbilirubinemia requiring phototherapy, (b) sepsis, (c) respiratory distress including sub-categories respiratory distress syndrome, severe respiratory (mechanical ventilation and or surfactant administration), persistent pulmonary hypertension of the newborn (PPHN), pneumothorax, and (d) birth asphyxia. The iNICU bed side interface has published data dictionary encapsulating various clinical diagnosis coded as per ICD definition^33^. Early sepsis was defined as culture-proven sepsis in the first 72 h or treatment with antibiotics for at least 5 days beginning by 72 h for the presumed sepsis regardless of the culture result. Late sepsis was defined as culture-proven sepsis after 72 h of age to discharge or treatment with antibiotics for at least 5 days after 72 h of admission^34^. All cases of Birth Asphyxia were outborn and were marked at the discretion of the provider and not as per standard definition^35^.

### Analysis and validation pipeline

**Figure 4:**
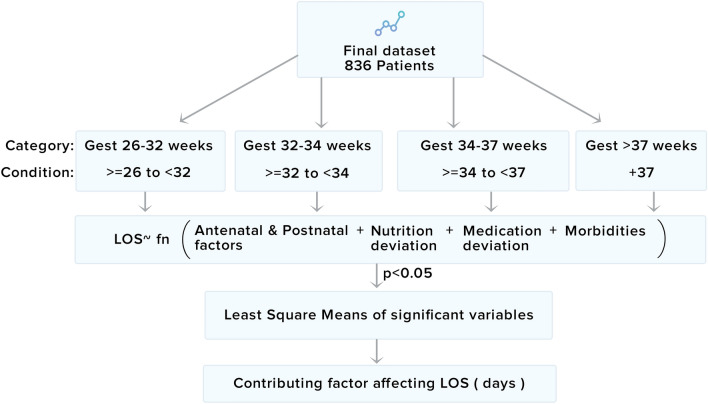
Structure for data analysis studying the effect of antenatal and perinatal factors, deviation in nutrition order, medication order, and medication.

Neonatal gestational age, which is highly correlated with many developmental and metabolic processes, is an indicator of neonatal outcomes^36,37^. The analysis pipeline step stratified data amongst different gestational age groups (Fig. 4).

The impact of independent variables (antenatal and perinatal, nutrition deviation, medication deviation, and clinical diagnosis) on LOS was studied with respect to their distribution (Supplementary method S1). Along with the normality of these distributions (Supplementary Figure S1 to S4) was their fitment with log, general linear models, and other transformed regression models was compared. We compared various models to find the most suitable one (Table S2) based on the Akaike Information Criterion (AIC), Bayesian Information Criterion (BIC), correlation coefficient, and degree of freedom^38^. The best model was found to be the *log model*. We used a randomly selected 80% population for model building and used the remaining 20% for testing. The number of iterations for randomization was fixed to the number of patients in the study. The RMSE of construction and testing dataset was 5.15 and 5.62, while the R^2^ value of the construction data set was 0.69, which is maximum compared to other models. The independent variables with p < 0.05 for the log model of each gestational category were selected as significant risk factors. These significant factors were again fed to the regression model and, their impact on LOS was calculated "in days" using the Least-Squares means (lsmean) package in R 3.5.3 (The R Foundation for Statistical Coding)^39^. The lsmean package provides Estimated Marginal Means (EMM) and Ordinary Marginal Means (OMM), which depends on a reference grid with all possible combinations of risk factors considered in LOS calculation^40^. Since the dataset considered in the current study was not balanced (26–32 weeks gestation category had only 85 patients, and not all possible combination of risk factors is seen in the dataset), we used ordinary marginal means, which assigns weights of each risk factors based on its occurrence seen in the dataset. It is more appropriate than marginal means in unbalanced datasets^41^.

In the lsmean analysis, the predicted LOS is generated as a linear model based on averages of dependent variables (such as antenatal, nutrition and medication deviations, and co-morbidities) over a reference grid. The reference grid is the set of all combinations of reference levels (overall dependent variables). The categorical dependent variable such as gender will have its reference levels as the unique possible male or female levels.

Whereas for continuous variable such as nutrition deviations, its reference level is assumed as its mean over the dataset (detailed steps of performing lsmeans is explained in Method S2). For each neonate, two vectors were considered while calculating the nutrition deviations for a “n” number of days. The first vector (× 1, × 2, …, xn) refers to nutrition value on daily basis according to ASPEN guidelines, and (× 1′, × 2′, …, xn′) referred to actual nutrition administered to the patient. We considered a deviation as a difference between prescribed and recommended guidelines and used natural Euclidean or L2 norm (Eq. 1).

1 $$Deviation \, factor = \sqrt {(x_{1} - x_{1}^{\prime } )^{2} + (x_{2} - x_{2}^{\prime } )^{2} + \cdots + (x_{n} - x_{n}^{\prime } )^{2} }$$

where, $$x_{1} , x_{2} , \ldots , x_{n} = {\text{Guidelines recommendation}},$$ $$x_{1}^{\prime } ,x_{2}^{\prime } , \ldots x_{n}^{\prime } = {\text{Doctors orders}}$$.

A standard operation in LS Mean based regression analysis is transforming the continuous variables numeric to categorical variables creating bins or quartiles^29^. Mathematically, the other norms (L^1^, L^2^, or L^∞^) are scaled form of the L^2^ norm and were equivalent to each other. The deviations were calculated in ml/kg/day on the daily basis, and these deviations consider the weight of neonate while prescribing the nutrition volume. After that, the sum of deviations on the daily basis was averaged over the LOS to calculate the spread of deviation (referred to as Deviation Factor) for a given patient). The aim of the current study is to estimate the effect “in days” of nutrition deviation on the prediction of LOS. Therefore, the deviation factor values were divided into four quartiles, and the highest quartile data (maximum deviation) was compared with the rest of the population (combined three quartiles referred to as “remaining”). This is done for the convenience to find the association of predicted LOS with respect to the “number of days” difference between two quartiles.

The model was trained on 12 months of consecutive patient data using antenatal, nutrition and medication deviations, and morbidities. The model performance was then assessed on subsequently captured 4 months of data. The best model (log model) was used with the significant risk factors for each gestation category and the LOS was predicted. During the validation stage, consultation with the clinical team leads to the development of bedside display interface to display the performance of validated model. The interface was designed to answer two clinical requirements (a) based on the gestation category of the neonate, provide the effect of associated risk factors in days (b) in case of varying (increasing or decreasing), highlight the corresponding risk factor in red color. In the current study, the interface was designed and implemented to show validation stage results, but it was not used at bedside for daily rounds.

## Results

### Descriptive statistics of the dataset

Our study presents the retrospective analysis of 16 months of data collected from two NICUs study sites. We used 12 months of data to train the LOS prediction model that included 836 patients from July 2018 to July 2019 (referred to as training data). We assessed the performance of our trained model on subsequently captured 4 months of data, including 211 patients from August 2019 to November 2019 (referred as validation data). The population characteristics and deviation data for nutrition and medication are displayed in Table 1 for baseline data and Table S3 for validation data. The antenatal steroid usage in lower gestation groups (< 34 weeks’) showed that only half the mothers received antenatal steroids. Details of maternal disease, infection, and risk factors distribution are given in Method S2. Data present about 80% of preterm (< 37 weeks’) were born by cesarean section, and half of the neonates < 34 weeks’ gestations were twins or triplets. The three most prevalent clinical diagnoses in the current study were hyperbilirubinemia requiring phototherapy (NNH), respiratory distress syndrome (RDS), and sepsis. The most significant deviations were seen with nutrition deviations amongst the smallest gestation group (26–32 weeks’). The medication deviation days were highest in the smallest gestation group (26–32 weeks’), which may be due to frequent use of caffeine and partially due to antibiotic deviations. The frequent prescription of caffeine was observed in this cohort, and its prophylactic usage for neonate under 32 weeks of gestation was observed. The 506 neonates in the “Medication Not Required” category were in NICU for clinical observation, growing preemies, and phototherapy. They received no medications but were administered vitamins and iron supplements.

**Table 1:** Baseline characteristics of the study population.

Characteristics	Gestation (weeks)			
	26–32 (n = 85)	32–34 (n = 211)	34–37 (n = 267)	> 37 (n = 273)
**Perinatal factors**				
Multiple pregnancy	45 (52.9)	116 (51.8)	59 (22.1)	5 (1.8)
**Antenatal**				
Infections	2 (2.3)	1 (0.45)	2 (0.7)	3 (0.9)
Maternal disease	27 (31.7)	78 (37)	59 (22.1)	17 (6.2)
Steroids	46 (54.1)	116 (54.8)	58 (21.7)	5 (1.83)
Risk factors	28 (32.9)	46 (21.8)	28 (10.4)	8 (2.9)
**Umbilical doppler**				
Abnormal	11 (12.9)	17 (7.6)	6 (2.1)	2 (0.6)
Normal	47 (55.2)	116 (55)	116 (43.4)	90 (33)
**Birth details**				
Gestation age*	30.07 (1.47)	33.1 (0.57)	35.4 (0.8)	38.5 (1.0)
Caesarean section	70 (82.3)	187 (88.6)	212 (79.4)	158 (57.9)
Need for PPV	6 (7.0)	7 (3.1)	5 (1.9)	6 (2.2)
Apgar 5 min, < 5	1 (1.1)	0	1 (0.3)	2 (0.73)
Gender, male	62 (72.9)	127 (60.2)	176 (65.9)	198 (72.5)
Inborn	67 (78.8)	138 (65.4)	171 (64)	95 (34.8)
Gestation, weeks*	30.1 (1.5)	33.1 (0.6)	35.5 (0.8)	38.5 (1)
Birth weight, g*	1425.4 (311.7)	1876.1 (347.8)	2236.4 (508.5)	2737 (537)
**Clinical diagnosis**				
Respiratory distress				
RDS	60 (70.5)	94 (44.5)	98 (36.7)	80 (29.3)
TTNB	16 (18.8)	22 (10.4)	40 (15)	28 (10.2)
MAS	0	0	2 (0.7)	7 (2.6)
Need for MV^#^	16 (18.8)	10 (4.7)	29 (10.9)	34 (12.5)
Pneumothorax	2 (2.3)	3 (1.3)	7 (2.6)	6 (2.2)
PPHN	0	0	0	2 (0.6)
Sepsis	33 (38.8)	14 (6.6)	29 (10.9)	34 (12.5)
NNH^$^	51 (60.0)	69 (32.7)	88 (33)	89 (32.6)
Asphyxia	0	0	2 (0.7)	20 (7.3)
**Nutrition**^‡^				
Energy deviation, kcal/kg				
IVth quartile	246.7 (67)	128.8 (66)	120.4 (53)	132.6 (47)
Remaining	143.9 (72)	46.9 (38)	40.7 (40)	39.2 (64)
Protein deviation, gram/kg				
IVth quartile	7.5 (1.3)	5.1 (1.7)	4.6 (1.7)	4.1 (1.6)
Remaining	4.3 (2.4)	2.4 (1.2)	1.9 (1.5)	1.4 (1.6)
**Medication**				
Medication received	70 (82.3)	68 (32.2)	82 (30.7)	110 (40.3)
No deviation	42 (49.4)	52 (24.6)	63 (23.5)	76 (27.8)
Deviation days^	9.5 (12.25)	2.5 (3.25)	4 (8)	3 (3.75)
Medication not required	15 (17.7)	143 (67.8)	185 (69.3)	163 (59.7)

The analysis of validation set was compared in all four gestational age groups with the Table 1 results (Supplementary Table S3). Except for birth weight and gestation, all other variables were comparable. There was no statistically significant difference in the baseline characteristics between testing and validation set in each of the gestational categories.

### Nutrition deviations/deviation in nutrition orders

Figure S5a–c show the intake in energy and protein orders (both ≤ 2500 g birth weight category and > 2500 g category) across gestation categories for their corresponding recommendations.

### Medicine deviations/deviation in medication prescription

Medicine dosage deviation with respect to NeoFax recommendations is displayed in Table S4a (top 10 medicines based on dosage are shown and complete listing in Table S4b). Although the current study did not evaluate the relationship of specific medication with the predicted LOS, Table S4a,b shows that the antibiotics and caffeine dosage have caused the highest deviation amongst all medicines and may have associative effect in predicted LOS. Caffeine overdose occurred in 5.2% (85/1604) of neonates in the lower gestation (26–32 weeks’) category. There was a large spectrum of antibiotics used across the two NICU. The LOS of patients receiving medications and its comparison with non-medicated patients is shown in Figure S6a.

Sepsis cases were not found in ‘medication not required category’ (Figure S10). Moreover, it was found that deviation is positively correlated with higher sepsis patient count.

Deviation in the frequency of medicines with respect to NeoFax recommendations is shown in Table S4c (top 10 medicines based on frequency are shown and complete listing in Table S4d). The corresponding LOS of patients is shown in Figure S6b. The trend of medication frequency deviations was similar to the dosage deviations across categories, and thus the only dosage was considered in further analysis.

### Clinical diagnosis across patient categories

All gestational age categories had neonates with RDS, hyperbilirubinemia requiring phototherapy, and sepsis as a major clinical diagnosis in decreasing order of prevalence (Figure S7). In the term category, 29.3% of neonates had RDS, while 7.3% had asphyxia. This might be due to the reason that 56% of data is from a rural site where mothers often report late for delivery, and some of the neonates might have suffered in-utero hypoxia resulting in unexplained RDS after birth. Moreover, rural sites often witness a higher number of out-born cases with unknown causes for respiratory distress like meconium aspiration and asphyxia that also gets labeled as RDS during data entry. The association effect of sepsis on LOS was found significant in the 32–34, 34–37, and > 37 weeks’ gestational age categories compared to other clinical diagnoses.

### Relationship of independent variables with predicted LOS (length of stay)

Gestational age found to be significant in predicting LOS in < 34 weeks neonates while it was not significant for neonates above 34 weeks’ of gestation (univariate analysis of LOS and gestation is shown in Figure S8). Table 2 shows the lsmeans analysis of significant independent variables and their relationship with predicted LOS (the complete list of variables with zero value (if significant) is shown in Table S5). Nutrition deviation adds 6 additional days in LOS for 26–32 weeks; gestation patients compared to median LOS. The medication deviation and clinical diagnoses are significant for LOS prediction in patient categories above 32 weeks’ gestation.

**Table 2:** Significant independent variables affecting LOS across various categories.

	26–32 weeks (n = 85)	32–34 weeks (n = 211)	34–37 weeks (n = 267)	> 37 weeks (n = 273)
Median LOS (IQR)	25 (16)	6 (7)	4 (3.5)	4 (3)
**Perinatal factors**				
Gestational category (1st quartile)	+ 19 (0.001)	+ 4 (0.000)		0 (0.023)
Gestational category (remaining quartile)	− 3 (0.001)	− 1 (0.000)		0 (0.023)
Male		+ 1 (0.01)	+ 1 (0.004)	
Caesarean delivery			+ 1 (0.038)	
NVD			0 (0.038)	
**Nutrition deviations**				
Energy deviation (4th quartile)	+ 6 (0.002)	+ 1 (0.05)		
Energy deviation (remaining)	− 7 (0.002)	− 1 (0.05)		
Protein deviation (4th quartile)			+ 1 (0.003)	+ 1 (0.014)
Protein deviation (remaining)			− 1 (0.003)	− 1 (0.014)
**Medication**				
Medication not required		0 (0.005)	0 (0.000)	− 1 (0.000)
Medication deviation				
Dose deviation		+ 7 (0.005)	+ 7 (0.000)	+ 4 (0.00)
Dose no-deviation		+ 3 (0.005)	+ 3 (0.000)	+ 2 (0.000)
**Clinical diagnosis**				
Severe RDS (invasive ventilation)			+ 5 (0.023)	+ 6 (0.000)
RDS with TTNB				+ 1 (0.019)
**RDS**				
Yes				+ 2 (0.048)
No				− 1 (0.048)
Sepsis		+ 9 (0.033)	+ 7 (0.000)	+ 7 (0.000)
NNH		+ 3 (0.001)	+ 1 (0.028)	

All values mentioned as “number of days” obtained after ls means analysis and (p-value).

*NVD* normal vaginal delivery, *RDS* respiratory distress syndrome, *TTNB* transient tachypnea of the newborn, *NNH* neonatal hyperbilirubinemia.

**Figure 5:**
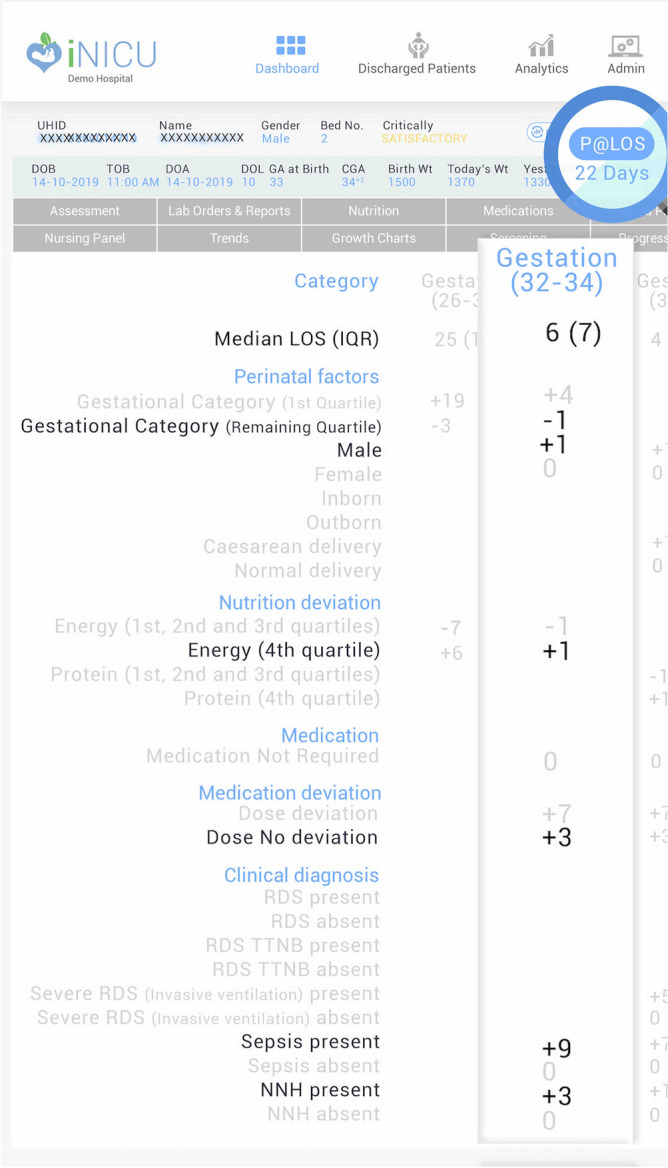
Bedside LOS prediction model displayed on iNICU interface, values marked in black represents the value applicable for the category.

**Figure 6:**
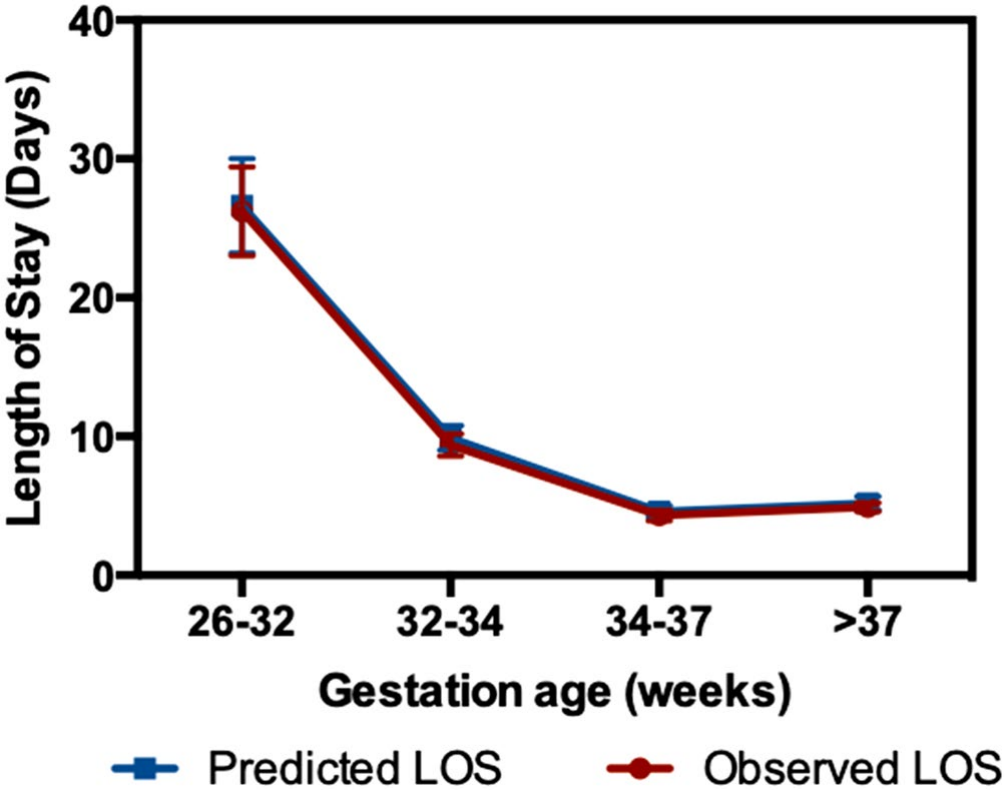
Model performance over 4 months validation data. R^2^ for < 32 gestation week is 0.95, for 32 to 34 is 0.74, for 34–37 is 0.62, for ≥ 37 is 0.64.

Based on the significant risk factors and associated effect in days on LOS for each gestation category, Fig. 5 represents the bedside interface. The interface depicts the predicted LOS value for a given patient with associated risk factors. The gestation category of an individual patient is zoomed, and associate risk factors are highlighted in bold black color. In comparison, the non-applicable risk factors for a given gestation are marked in light grey color. The selected gestation category shows the risk factors with its corresponding weight (in days) by which it affects the LOS. The predicted LOS (P@LOS) comparison with observed LOS is shown in Fig. 6 as the Root Mean Square Error (RMSE) curve across gestation categories. Table 3 shows the predicted LOS model statistics using different risk factor combinations of Antenatal, Perinatal, Nutrition, Medication and Diagnoses Details variables. The corresponding AIC/BIC values for different prediction models and R-squared value were analyzed as comparison of model characteristics. It was seen that most of these models have AIC/BIC value in similar ranges so the most optimum R-square value generating least difference between observed and predicted LOS value was used to select the best model (Table S1a–d). The R-squared varied from 49 to 94.5% (Fig. 6) across gestation categories.

**Table 3:** Difference between observed and predicted LOS (in days).

	26–32 weeks	32–34 weeks	34–37 weeks	> = 37 weeks
Predicted average LOS^a^	26.2 (3.2)	9.4 (0.8)	4.3 (0.3)	4.9 (0.4)
Observed average LOS	26.6 (3.4)	9.9 (0.9)	4.6 (0.4)	5.2 (0.5)
Risk factors used for prediction*				
1. Antenatal and perinatal	0.8	1.6	0.8	0.8
2. Antenatal and perinatal and diagnoses	0.5	1.2	0.7	0.5
3. Antenatal and perinatal and nutrition	0.4	1.0	0.4	0.6
4. Antenatal and perinatal and medication	0.5	0.9	0.7	0.2
5. Antenatal and perinatal, nutrition and diagnoses	0.3	0.7	0.4	0.3
6. Antenatal and perinatal, medication and diagnoses	0.3	1.7	0.6	1.1
7. Antenatal and perinatal, nutrition, medication and diagnoses	0.4	0.5	0.4	0.1

All values mentioned as “number of days”.

^a^Mean (SD).

## Discussion

Improving LOS prediction is a top priority in CCU settings for resource planning, reducing possibility of hospital-acquired infections, and improving financial efficiency. When considering fragile premature neonates in the NICU, this becomes even more significant. Scoring systems, such as SNAPPE, SNAP II^42^, and CRIB II^43^, have been used to assess illness severity and predict the morbidity, mortality, and prognosis. These scoring systems, which incorporate data from the first few hours of patient stay or specific clinical events, have been used to predict LOS^44,45^. In the current study, LOS could be predicted across different neonatal gestational age categories using a bedside interface based on antenatal and perinatal information, medication and nutrition details, and clinical diagnosis details. Since the adoption of the EMR in hospitals has been prolific worldwide, the application of a bedside interface in various NICU settings using a variety of data parameters shows promise to predict LOS.

The presented model predicts the LOS at different stages of patient stay in the NICU. At admission, the clinician can use the predicted value to counsel the parents about the neonate’s LOS in the NICU. During the course of hospital stay, the tool will provide a daily update to the healthcare staff if any change is observed in the predicted pattern of LOS in response to any deviation in the nutrition and medication administered from the recommended guidelines. Nutrition and medications are significant factors affecting the growth of neonates in the NICU. Persistent nutrition deficits can directly impact the neonate's overall growth rate and neurodevelopment. We presented deviation of prescribed macronutrients (such as energy, proteins) (Table S6a,b) and micronutrients (such as vitamin A and D, calcium, phosphorus, and iron) with respect to prescribed dose over time (Figure S9). The micronutrients were not considered during overall LOS prediction, as both the NICUs in the study did not provide micronutrients in the PN solution. Individual EN analysis of micronutrients was documented in supplementary sections Table S6c–g. The univariate analysis of LOS with medication deviation showed that caffeine and antibiotics were the most prevalent types of medications with deviations.

The developed LOS prediction model presents the median LOS of 25 days for 26–32 week gestation. Since these patients stay in NICU for prolonged period for developmental care, the morbidities do not associate with increased LOS. The lowest gestation and energy deviation accounted for 19 and 6 additional days respectively in 26–32 week gestation category. Moreover, the male gender adds an additional day in the predicted LOS of 32–37 weeks along with associative effect of NNH. For gestations between 32 weeks and above, categories show relationship with severe RDS, sepsis, and medications. The performance of developed model is determined by comparing the predicted and observed LOS, and it was inferred that the model using combination of antenatal and perinatal, nutrition, medication and diagnoses is the most optimum for all gestation categories. The LS mean based regression model predicts LOS with less than 0.5 day of difference with observed LOS for all gestation categories.

We demonstrated a LOS relationship with deviation in nutrition (energy and protein), medication (frequency and dosage), and clinical diagnosis, but further studies are needed to validate these findings. The results of the current study only address the association of independent risk factors with predicted LOS. The predictive models are not necessarily good causal models. The “nutrition and medication deviations” may not always imply an inadvertent deviation, but in some cases, they may have been purposeful due to individual patient circumstances. In these cases, it is not the deviation itself that may be contributing to increased LOS, but rather the underlying clinical condition that leads to the rationale for the deviation, as well as contributing to the increased LOS. Whether it is a cause or an association, the relationship between the deviation and increased LOS would still be present. Therefore the relationship of caffeine deviation and increased LOS for 26–32 weeks’ gestation group (Table S4a) needs further use of deterministic models to study causality. The severity of infection is not considered in this study, which may be associated with a high dose of antibiotics. Our study has certain limitations as our results are limited by risk factors representing clinical practice variations of only two NICUs. Our study population contained only 85 patients in 28–32 weeks’ gestation; so future LOS prediction studies will need to include larger sample sizes to determine the impact of various clinical parameters in the different gestational age groups. There was a high prevalence of cesarean births across all gestations, which may have influenced the results, so our findings may not apply in settings with lower rates of cesarean births. External validation in different NICU settings and varying clinical practices will further strengthen the findings of the current study. The effects of an overdose of medication such as, e.g. hepatotoxicity and nephrotoxicity were not considered in this study. The current study did not include the severity of morbidities (sepsis, RDS, and NNH) in the model. However, it instead only included their incidence, which may have resulted in the lack of utilization of available data and lower accuracy of prediction. In current study design, a one-time non-significant overdose would not categorize a patient in the highest quartile. The objective of the current study was to predict LOS based on the NICU’s environment existing practices and highlighting its relationship with independent risk factors. This will remain a limitation in this kind of model. Our data set did not have extreme deviations, however in a given data set if there are subjects with extreme deviations, their impact on LOS can be evaluated.

The major strength of this model is that it demonstrates the capability to learn from an individual NICU’s clinical data to build a good prediction model for LOS. Further studies are needed to establish the causal relationship between these entities to establish the role of predicted LOS in improving operational efficiency. It is contemplated that as the dataset for the LOS prediction model becomes voluminous, it can identify patterns of treatment regimen that may be most suited for a neonate in a given gestation category. This in future, studies may enable reduced overdose of antibiotics, and improved clinical outcomes, which could ultimately result in reduced emotional and financial anxiety for parents^34,38^. This may lead to the development of an early alert system regarding deviations in medications or nutrition that eventually may help to improve the LOS.

## Supplementary Information

Supplementary Information

## Data Availability

The code that underpins the prediction of LOS using various risk factors in ICU is openly available. The drive containing the code (Java and R) used to generate the descriptive statistics and tables included in this paper are available at: https://github.com/los-paper1/CHI-LOS. README.md file has all the scripts-related comments and other steps for executing the code.

## Abbreviations

NICU: Neonatal intensive care unit
ICU: Intensive care unit
LOS: Length of stay
EHR: Electronic health record
EMR: Electronic medical record
CPAP: Continuous positive airway pressure
ASPEN: American society for parenteral and enteral nutrition
ESPGHAN: European society for paediatric gastroenterology hepatology and nutrition
PPHN: Persistent pulmonary hypertension of the newborn
APGAR: Appearance, pulse, grimace, activity, and respiration
EMM: Estimated marginal mean
OMM: Ordinary marginal mean
AIC: Akaike information criterion
BIC: Bayesian information criterion
PPV: Positive pressure ventilation
TTNB: Transient tachypnea of the newborn
MAS: Meconium aspiration syndrome
MV: Mechanical ventilation
NNH: Neonatal hyperbilirubinemia
PN: Parenteral nutrition
TPN: Total parenteral nutrition
SNAP: Score for neonatal acute physiology
CRIB: Clinical risk index for babies
SNAPPE: Score for neonatal acute physiology-perinatal extension
NVD: Normal vaginal delivery
IQR: Interquartile range
TcB: Transcutaneous bilirubin
RDS: Respiratory distress syndrome
iNICU: Integrated neonatal intensive care unit
APACHE: Acute physiology and chronic health evaluation
SAPS: Simplified acute physiology score
MPM: Mortality probability model
