# Supplementary material for: Designing a bed-side system for predicting length of stay in a neonatal intensive care unit
Source: Sci Rep. 2021 Feb 8;11:3342. doi: 10.1038/s41598-021-82957-z (PMC7870925; doi:10.1038/s41598-021-82957-z)
Supplement: Supplementary file 1 — Supplementary Information [file 41598_2021_82957_MOESM1_ESM.docx]

**Supplementary Material**

**Designing a bed-side system for predicting length of stay in a neonatal intensive care unit**

Harpreet Singh^1*^, Su Jin Cho^2^, Shubham Gupta^1^, Ravneet Kaur^1^, Sunidhi S.^1^, Satish Saluja^3^, Ashish Kumar Pandey^4^, Mihoko V. Bennett^5,6^, Henry C. Lee^5,6^, Ritu Das^1^, Jonathan Palma^5^, Ryan M. McAdams^7^, Avneet Kaur^8^, Gautam Yadav^9^, Yao Sun^10^

**Affiliations:**

^1^Child Health Imprints (CHIL) Pte. Ltd.;

^2^Department of Pediatrics, Ewha Womans University School of Medicine, Seoul, Korea, ^3^Department of Neonatology, Sir Ganga Ram Hospital, New Delhi, India;

^4^Department of Mathematics, Indraprastha Institute of Information Technology, New Delhi, India;

^5^Division of Neonatal and Developmental Medicine, Department of Pediatrics, Stanford University, Stanford, California;

^6^California Perinatal Quality Care Collaborative, Stanford, California;

^7^Department of Pediatrics, University of Wisconsin School of Medicine and Public Health, Madison, USA;

^8^Department of Neonatology, Apollo Cradle Hospitals, New Delhi, India;

^9^Department of Pediatrics, Kalawati Hospital, Rewari, India; and

^10^University of California, San Francisco, USA

***Corresponding author:**

Dr. Harpreet Singh

E-mail: [harpreet@childhealthimprints.com](mailto:harpreet@childhealthimprints.com)

Child Health Imprints (CHIL) Pte. Ltd,

14 Robinson Road, Far East Finance Building,

Singapore 048545

Telephone: +91-9910861112

**Supplementary Method S1**

After studying the distribution of each variable and the normality of these distributions we have tried log and other transformed regression models along with general linear models. The plots of all significant variables (for conciseness) are presented from Figure S1 to S4). Thereafter, the study considered various regression models to find the most suitable one (Table S2) based on the Akaike Information Criterion (AIC), Bayesian Information Criterion (BIC), correlation coefficient, and degree of freedom. The best model was found to be the *log model*.

The selected log model was then used with the significant risk factors for each gestation category and the LOS was predicted. Table 3 shows the predicted LOS model statistics using different risk factor combinations of Antenatal, Perinatal, Nutrition, Medication and Diagnoses Details variables. The corresponding AIC/BIC values for different prediction models and R-squared value were analyzed as comparison of model characteristics. It was seen that most of these models have AIC/BIC value in similar ranges so the most optimum R-square value generating least difference between observed and predicted LOS value was used to select the best model (Table S1 (a) to (d).

The predicted LOS (P@LOS) was compared with observed LOS in Figure 6 as the Root Mean Square Error (RMSE) curve across gestation categories.

1. For 26 - 32 weeks


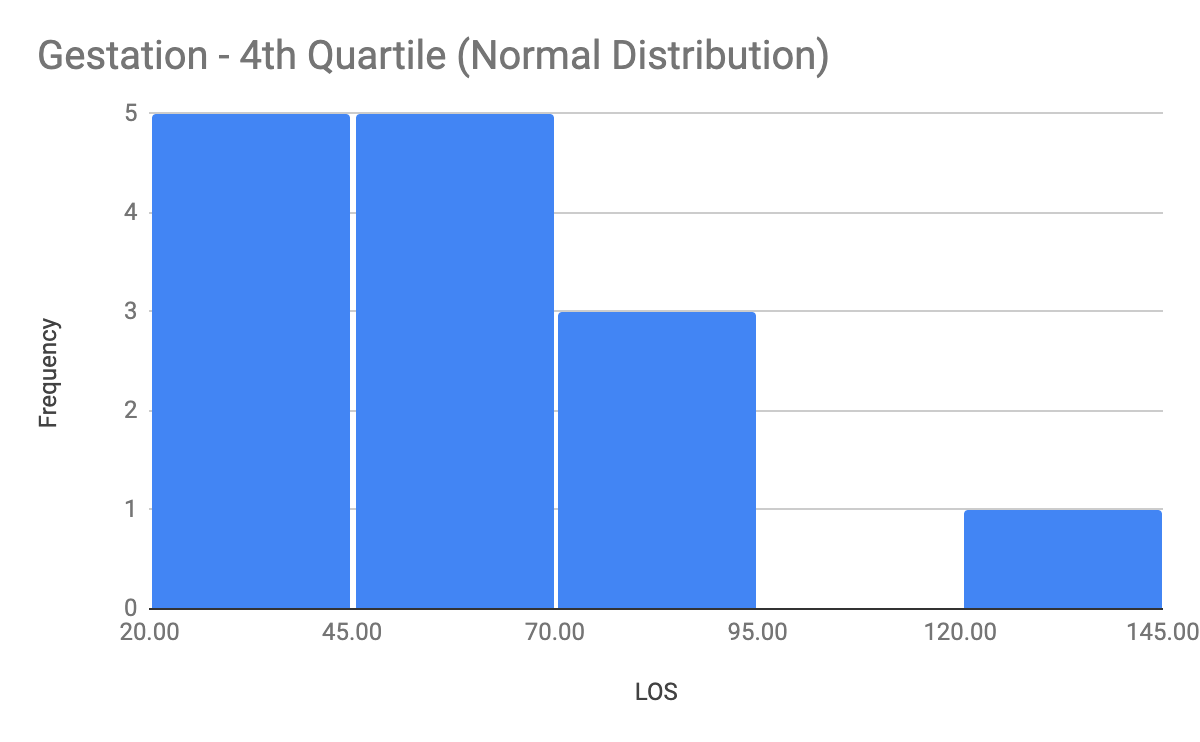


Figure S1(a): Normal distribution of 26-32 weeks gestation (first quartile)


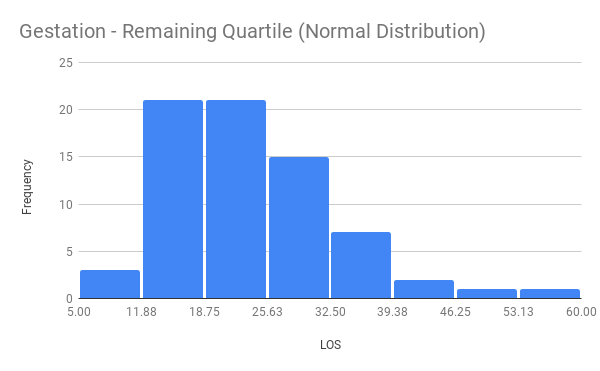


Figure S1(b): Normal distribution of 26-32 weeks gestation (remaining quartile)


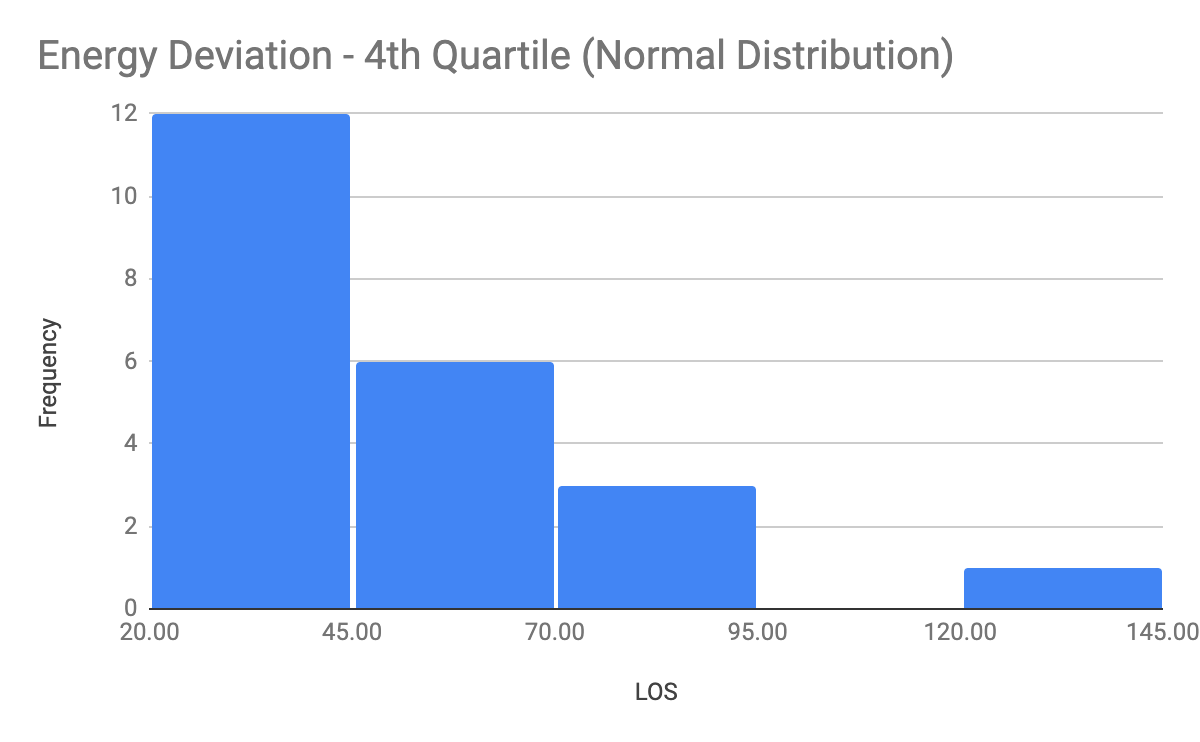


Figure S1 (c): Normal distribution of energy deviation of 26-32 weeks gestation (fourth quartile)


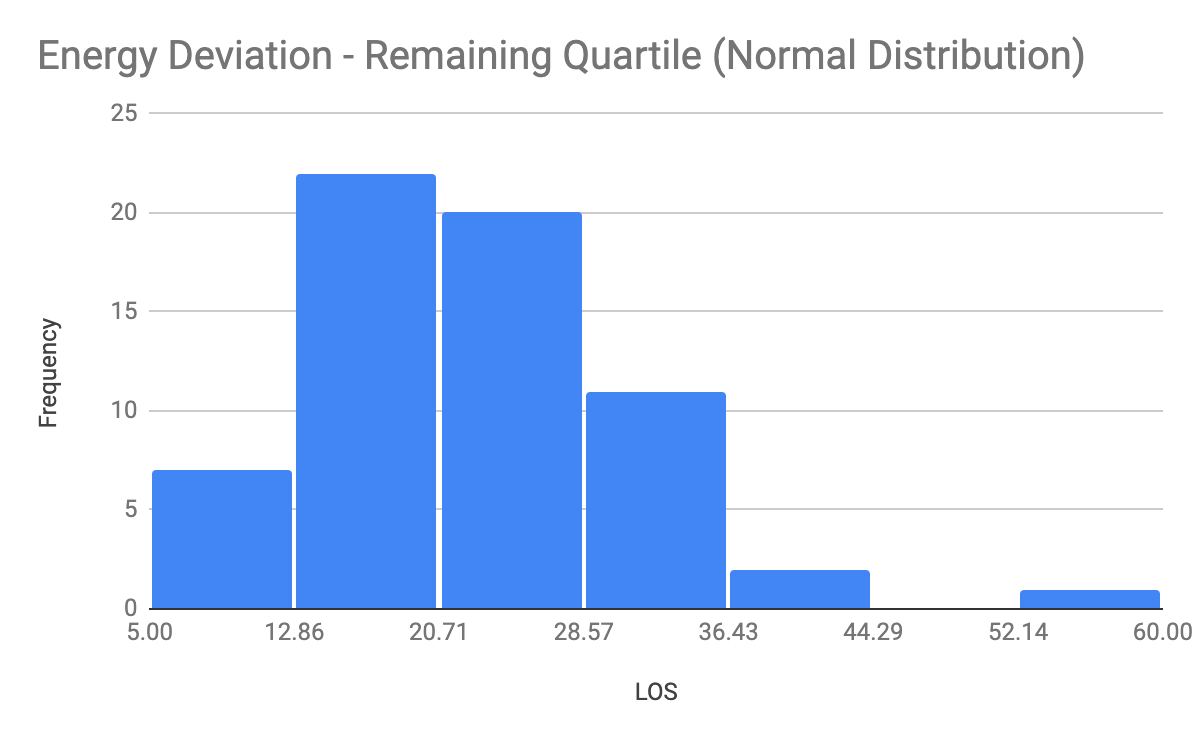


Figure S1 (d): Normal distribution of energy deviation of 26-32 weeks gestation (remaining quartile)

2. For 32 - 34 weeks


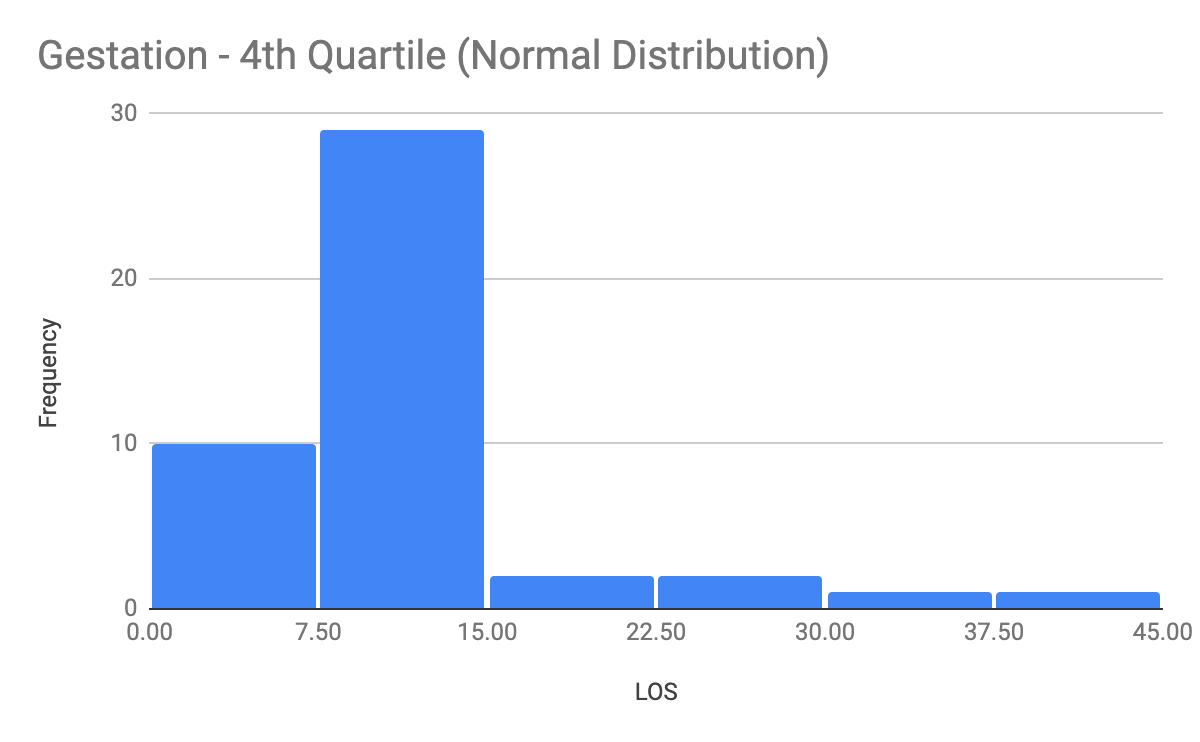


Figure S2 (a): Normal distribution of 32-34 weeks gestation (first quartile)


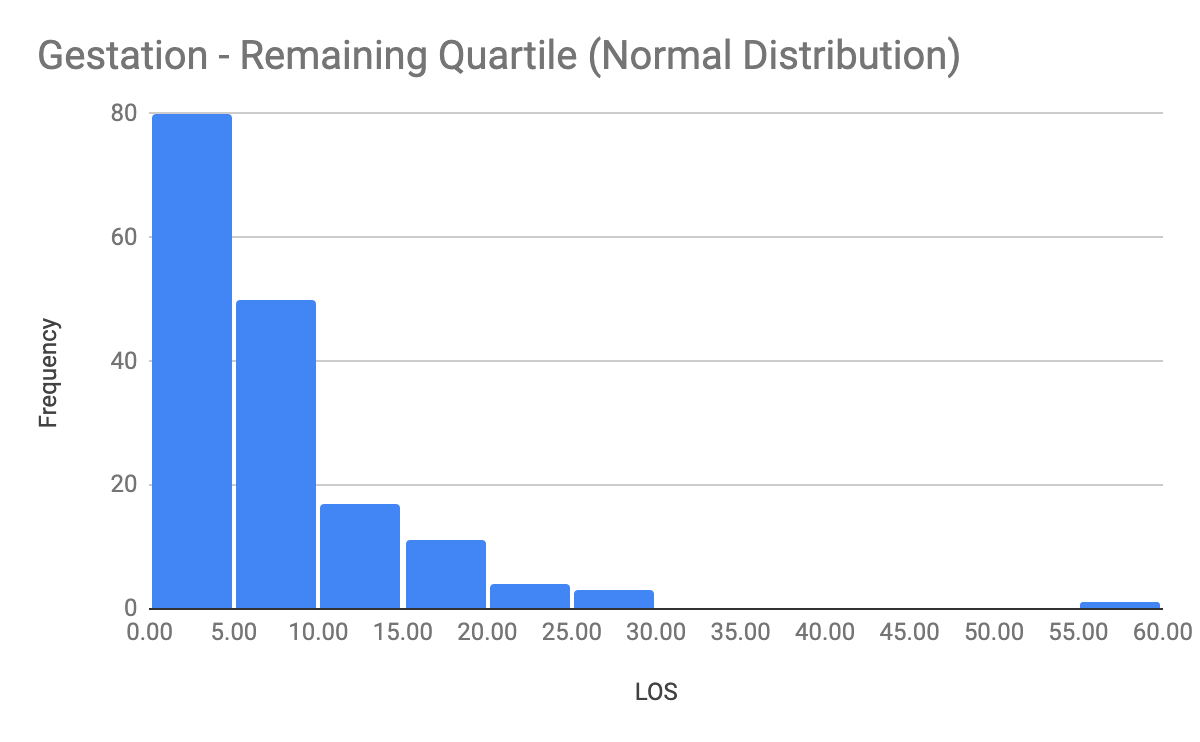


Figure S2 (b): Normal distribution of 32-34 weeks gestation (remaining quartile)


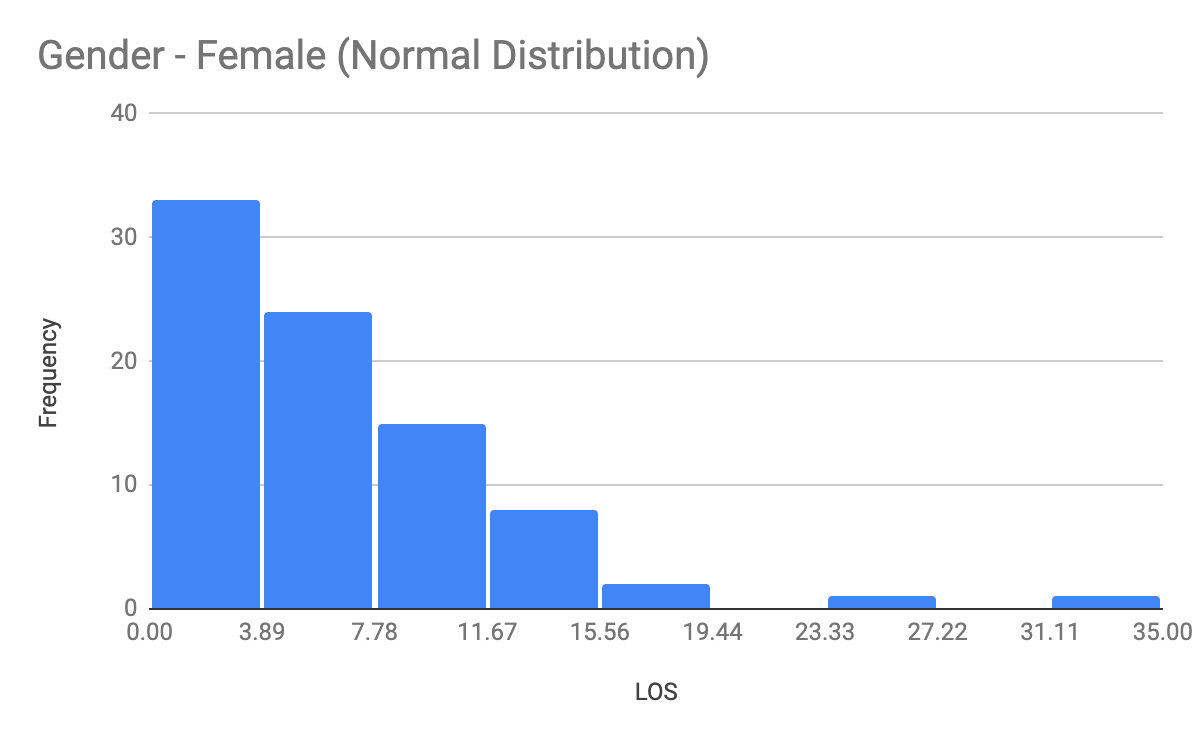


Figure S2 (c): Normal distribution of female of 32-34 weeks gestation


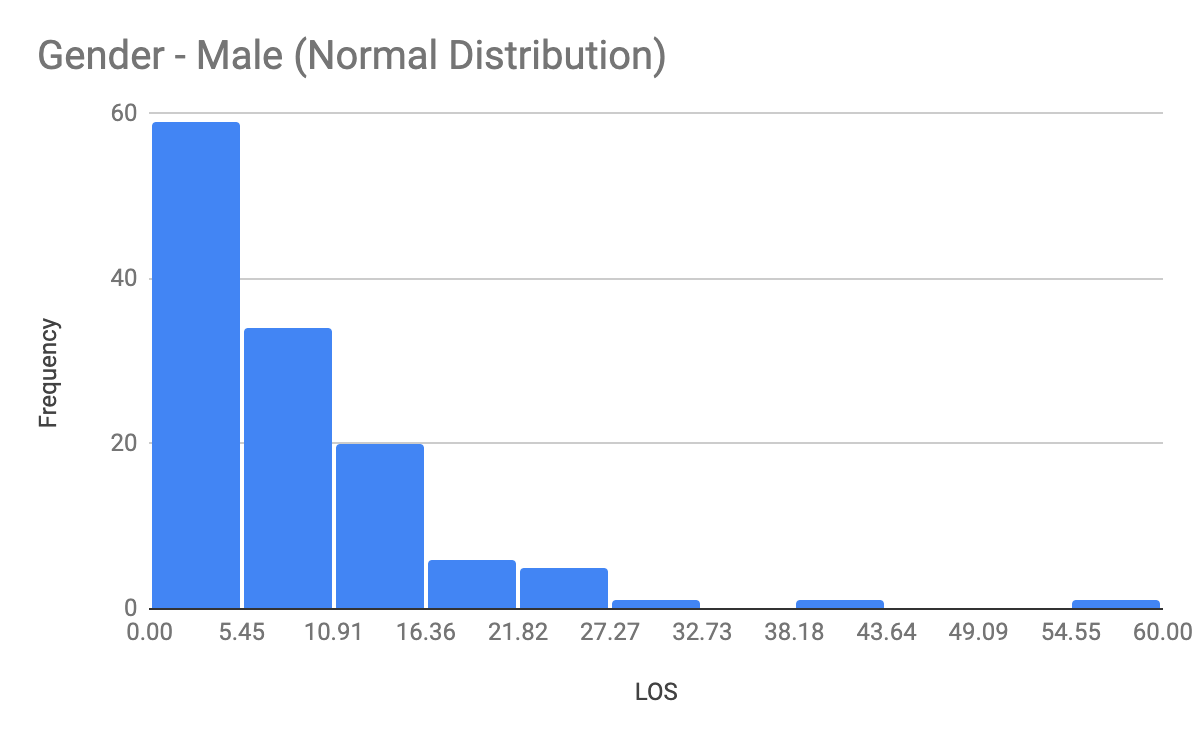


Figure S2 (d): Normal distribution of female of 32-34 weeks gestation


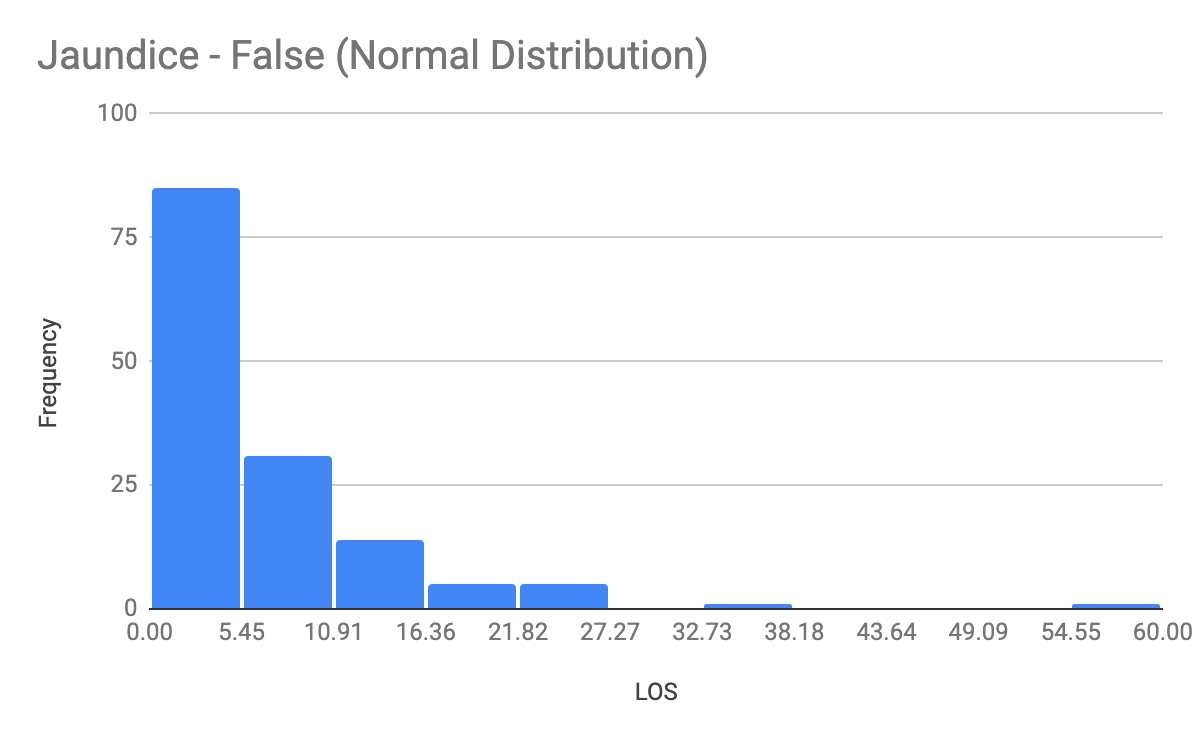


Figure S2 (e): Normal distribution of jaundice (false cases) of 32-34 weeks gestation


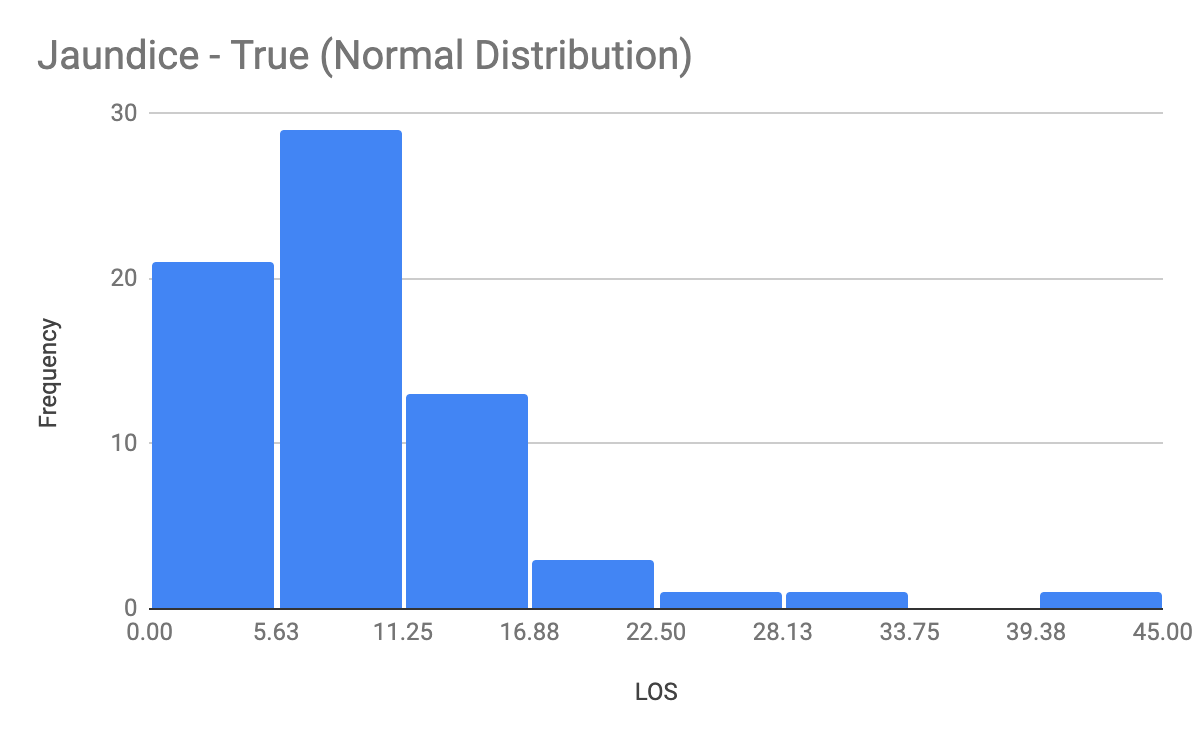


Figure S2 (f): Normal distribution of jaundice (true cases) of 32-34 weeks gestation


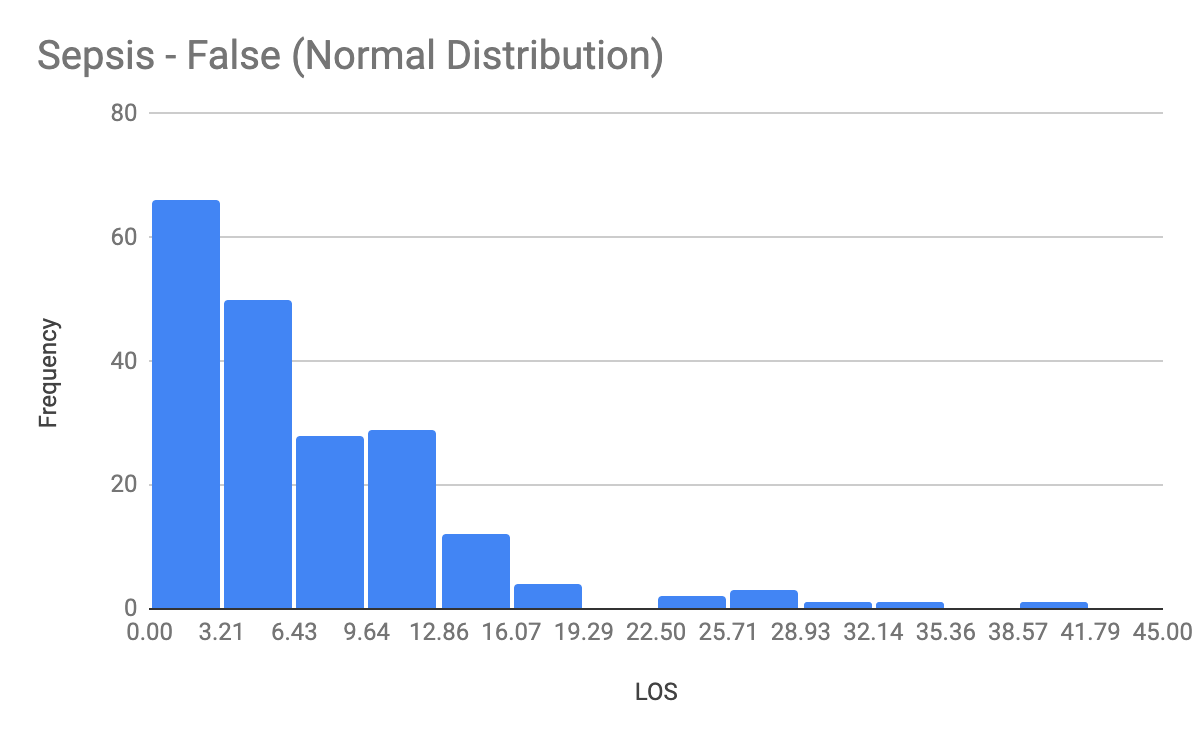


Figure S2 (g): Normal distribution of sepsis (false cases) of 32-34 weeks gestation


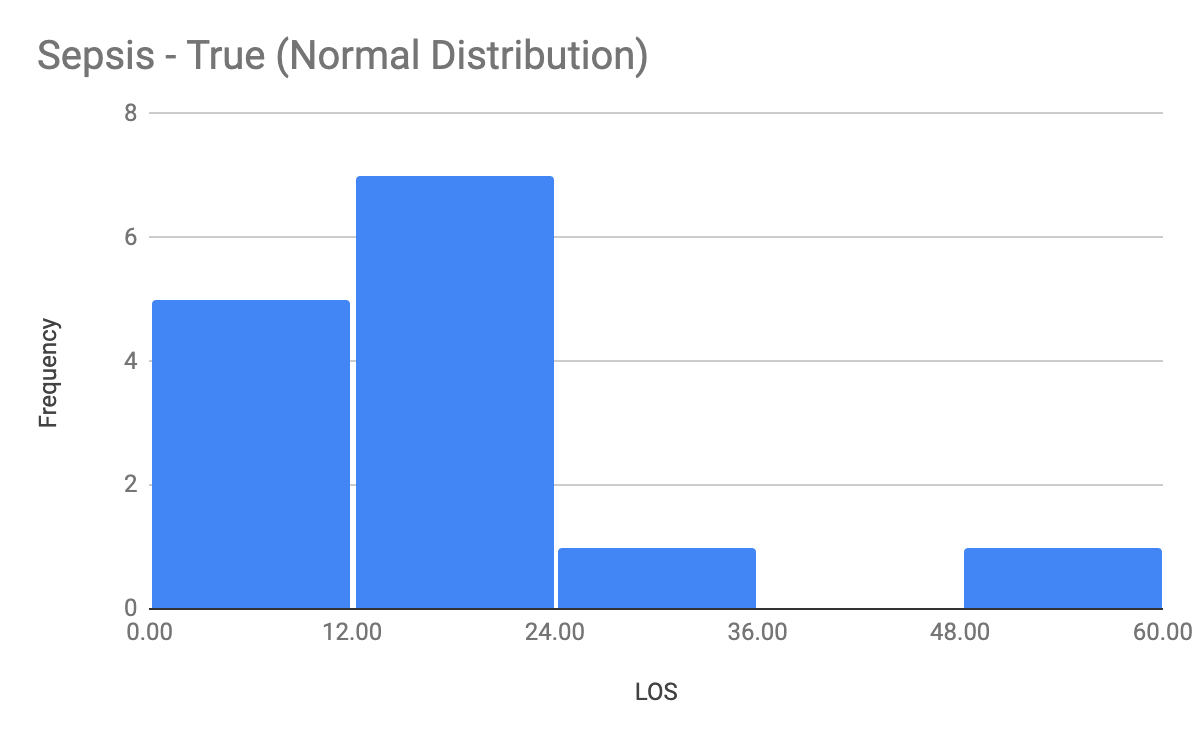


Figure S2 (h): Normal distribution of Sepsis (true cases) of 32-34 weeks gestation


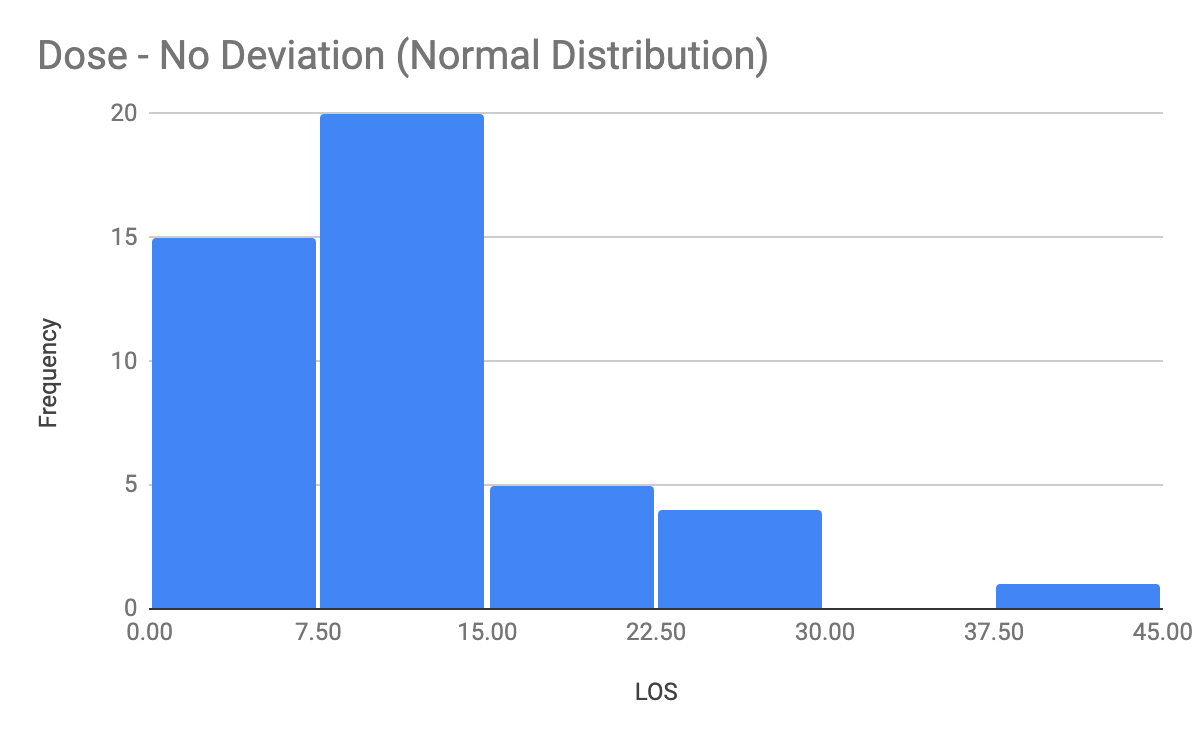


Figure S2 (i): Normal distribution of medication dose (no deviation) of 32-34 weeks gestation


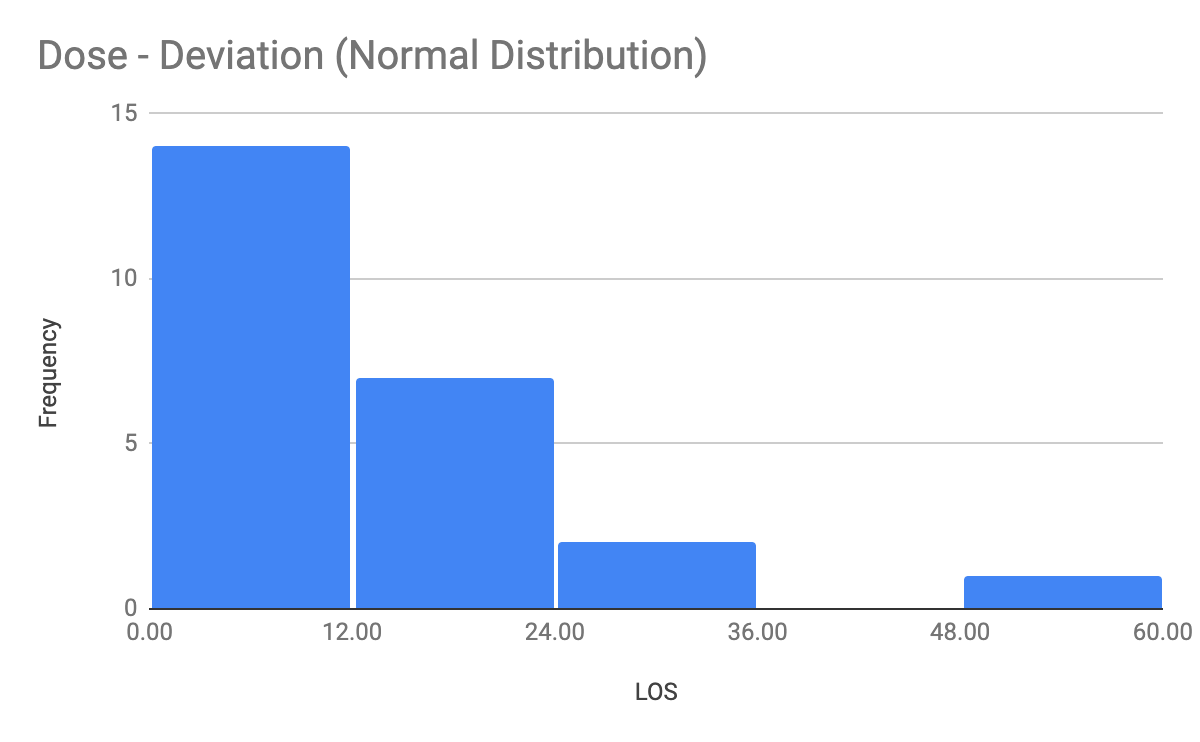


Figure S2 (j): Normal distribution of medication dose (deviation) of 32-34 weeks gestation


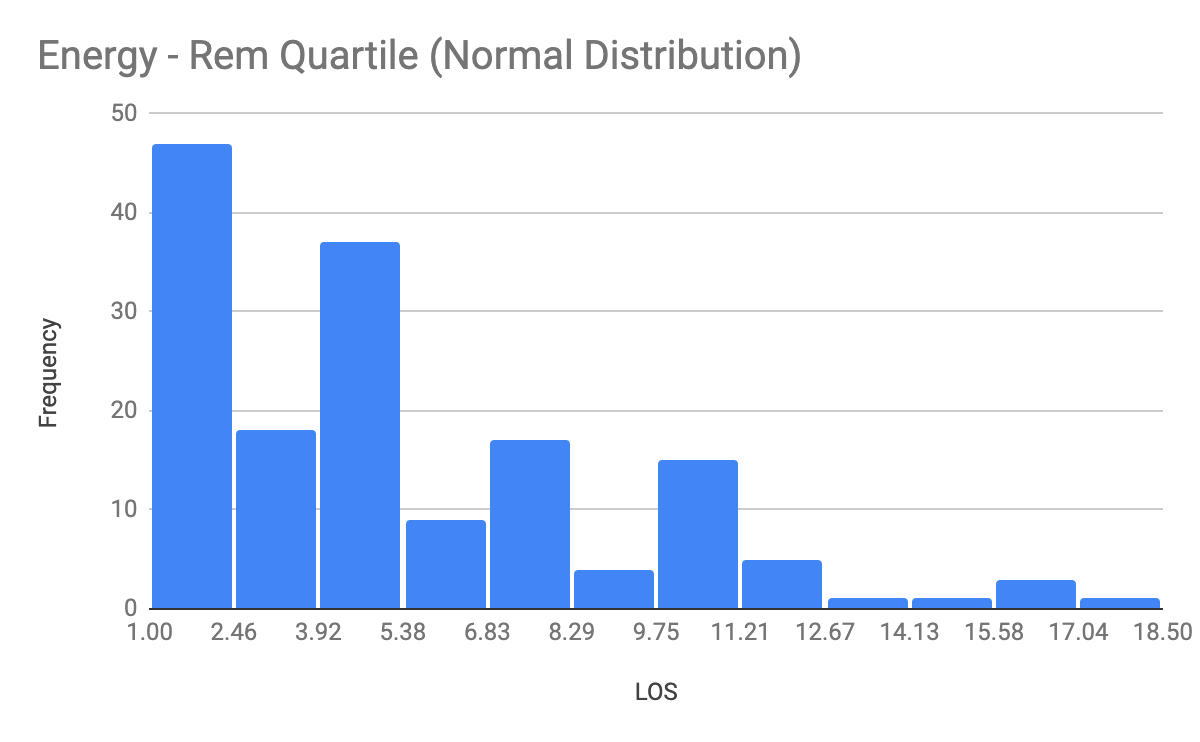


Figure S2 (k): Normal distribution of energy deviation of 32-34 weeks gestation (remaining quartile)


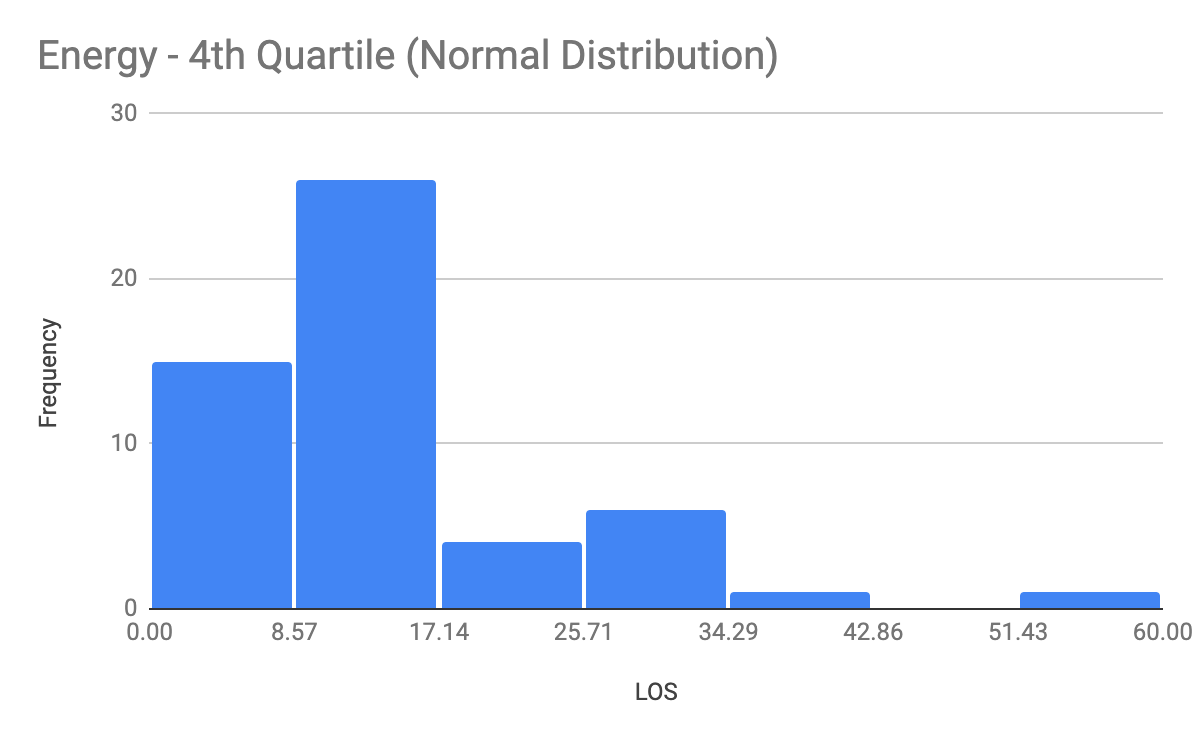


Figure S2 (l): Normal distribution of energy deviation of 32-34 weeks gestation (fourth quartile)

3. For 34 - 37 weeks


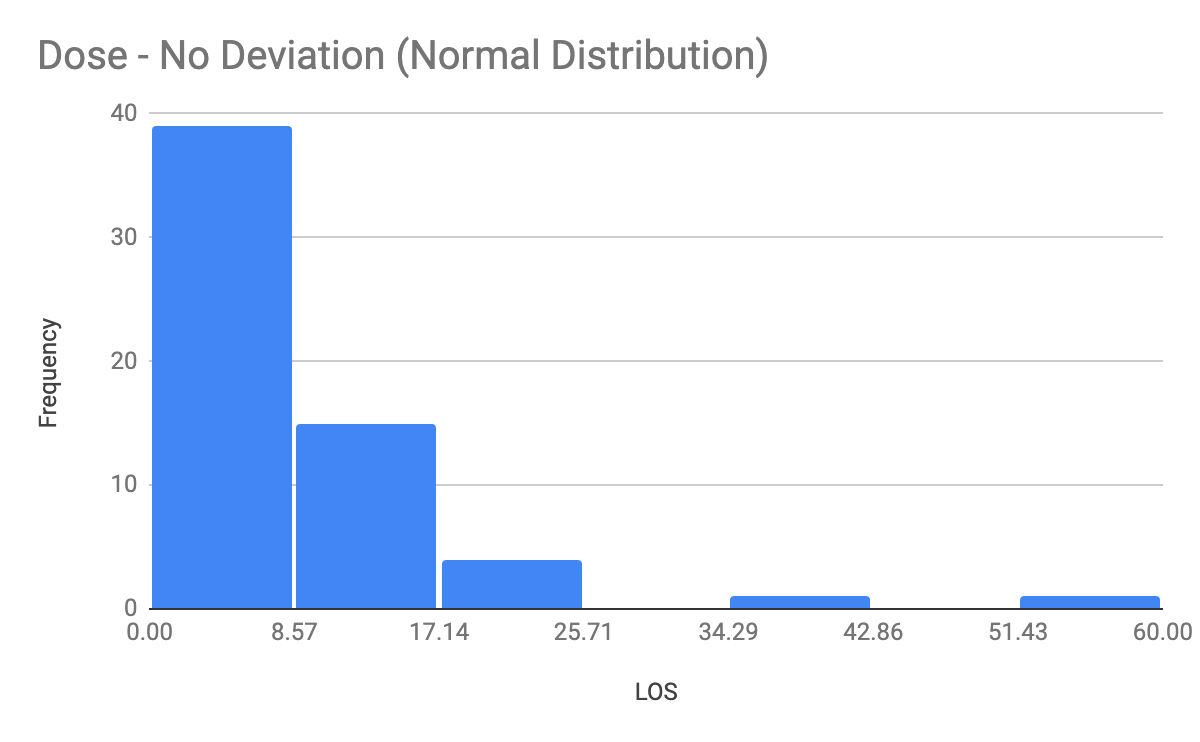


Figure S3 (a): Normal distribution of medication dose (no deviation) of 34-37 weeks gestation


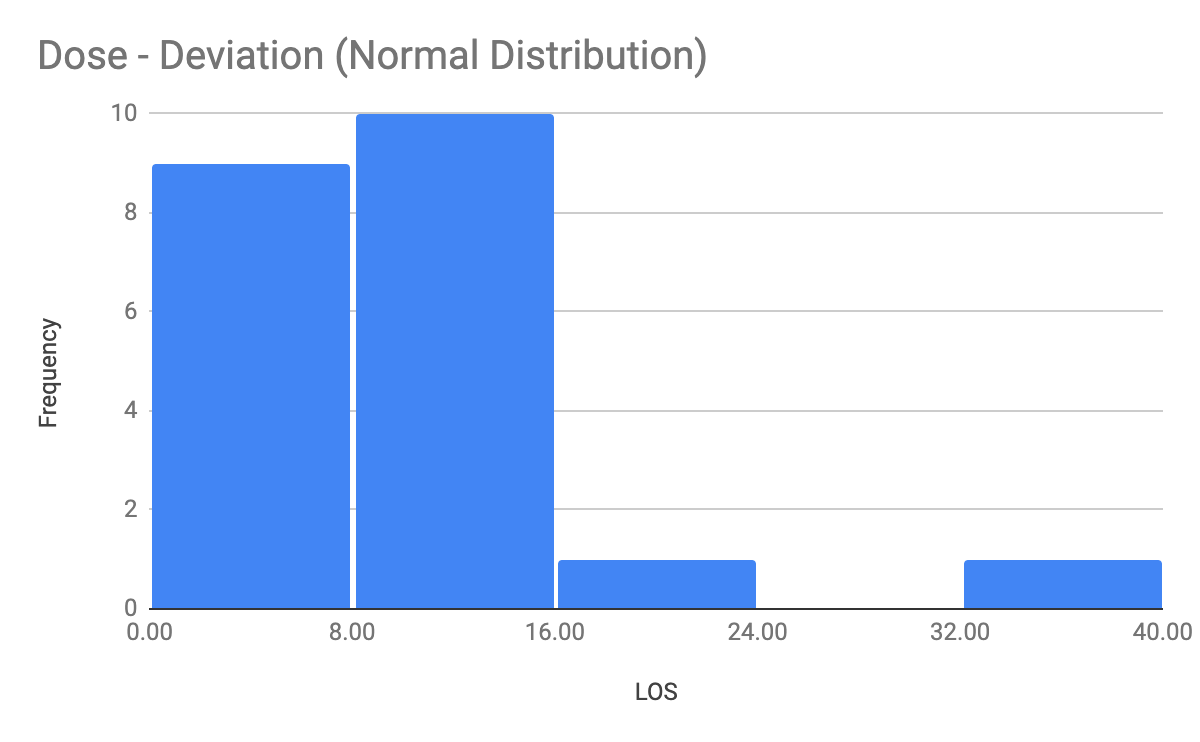


Figure S3 (b): Normal distribution of medication dose (deviation) of 34-37 weeks gestation


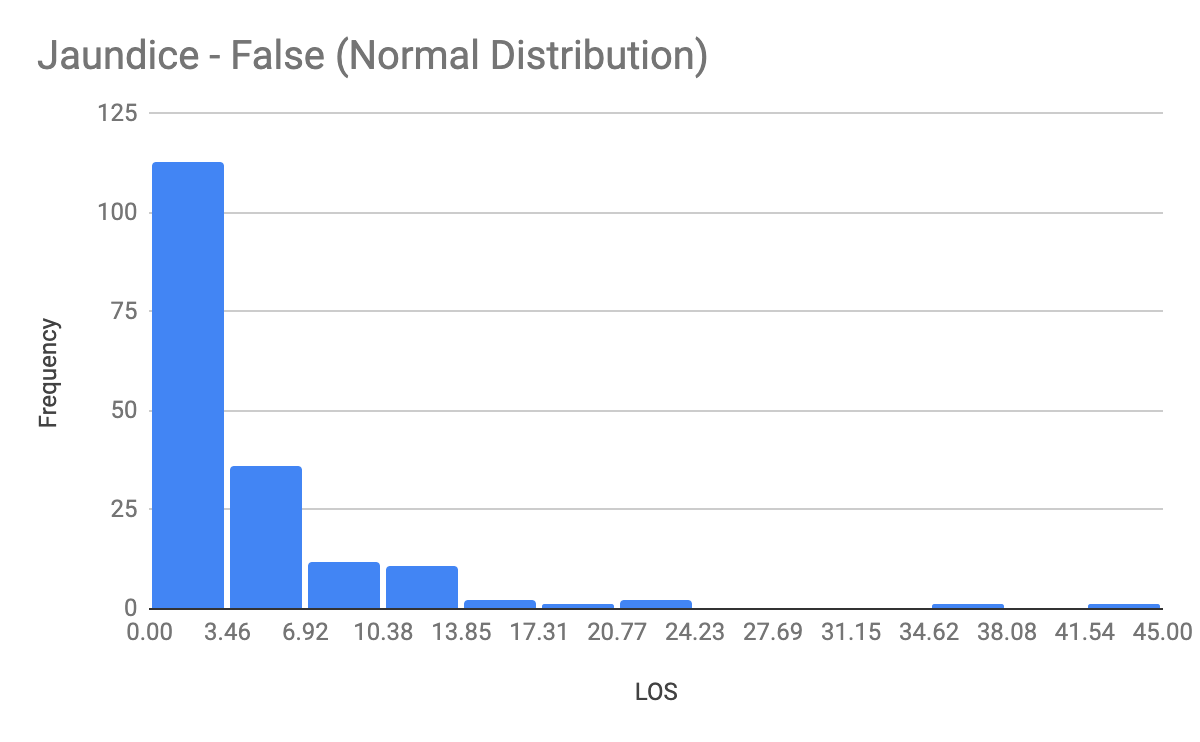


Figure S3 (c): Normal distribution of jaundice (false cases) of 34-37 weeks gestation


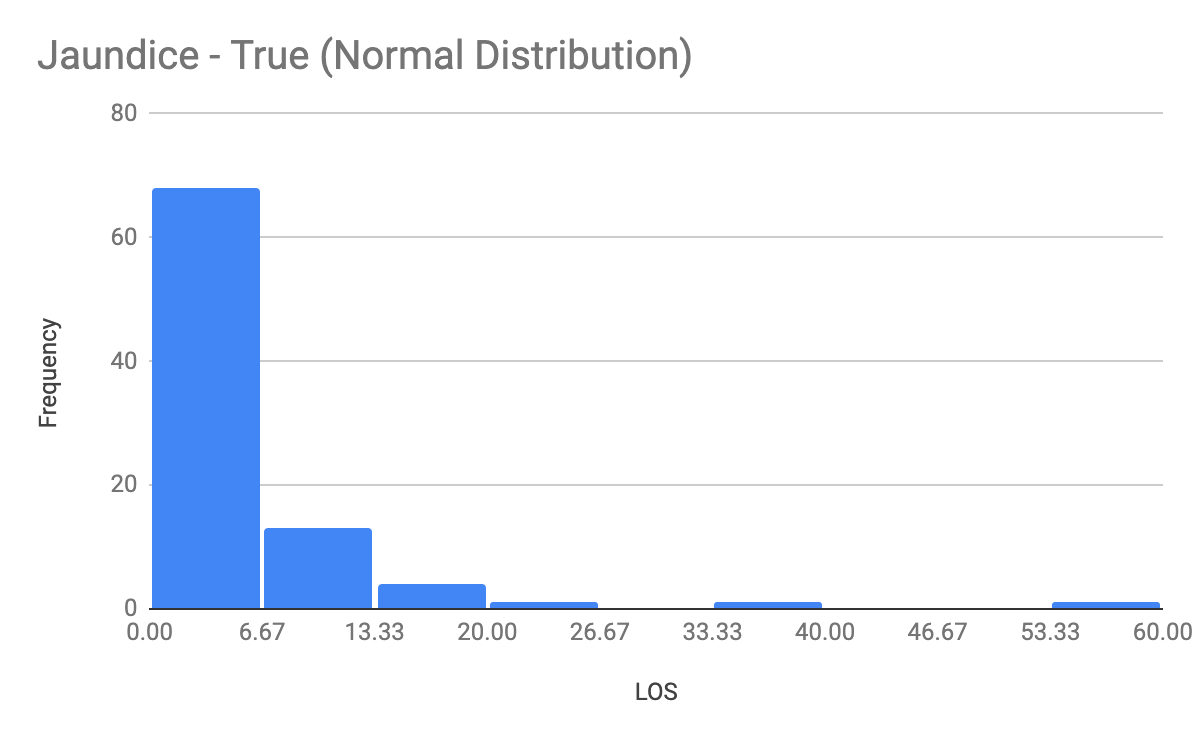


Figure S3 (d): Normal distribution of jaundice (true cases) of 34-37 weeks gestation


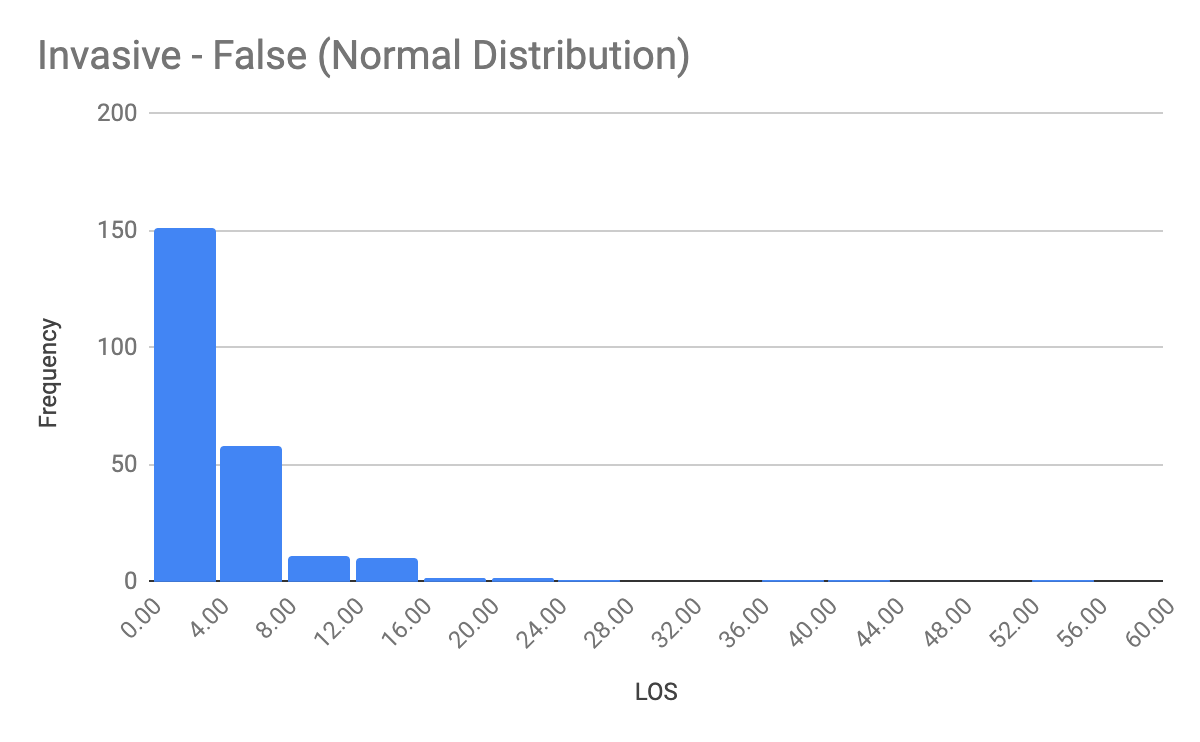


Figure S3 (e): Normal distribution of invasive (false cases) of 34-37 weeks gestation


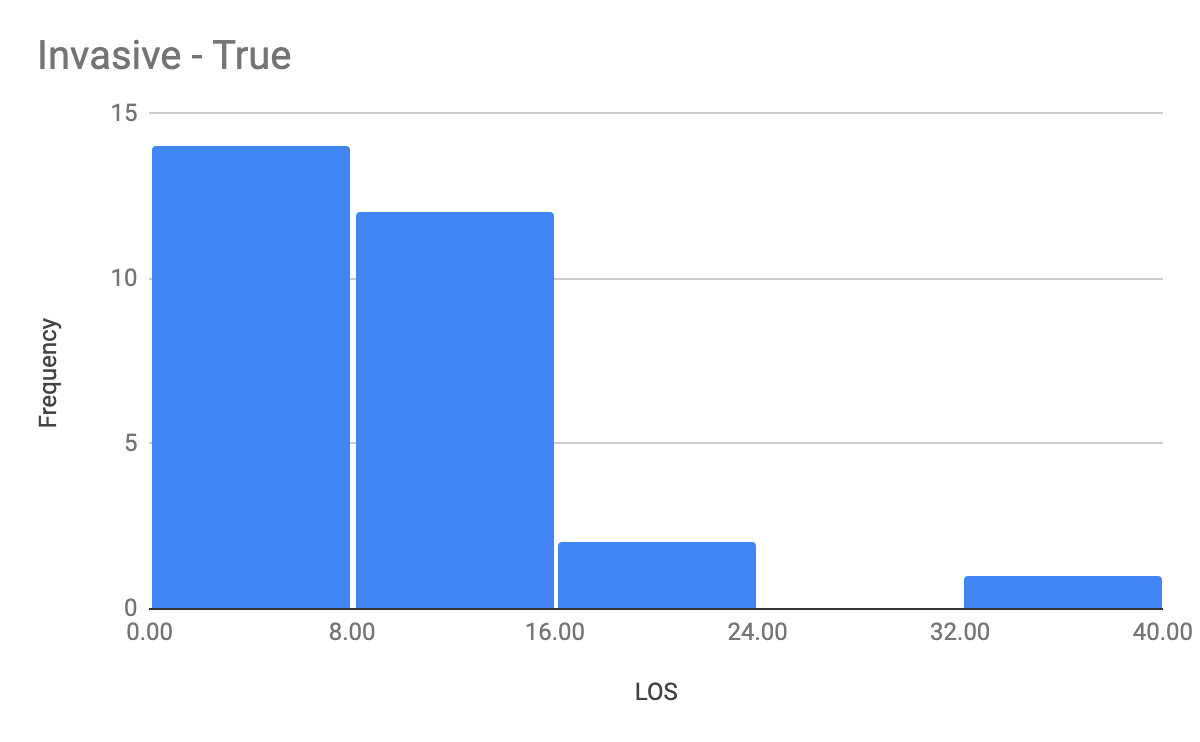


Figure S3 (f): Normal distribution of invasive (true cases) of 34-37 weeks gestation


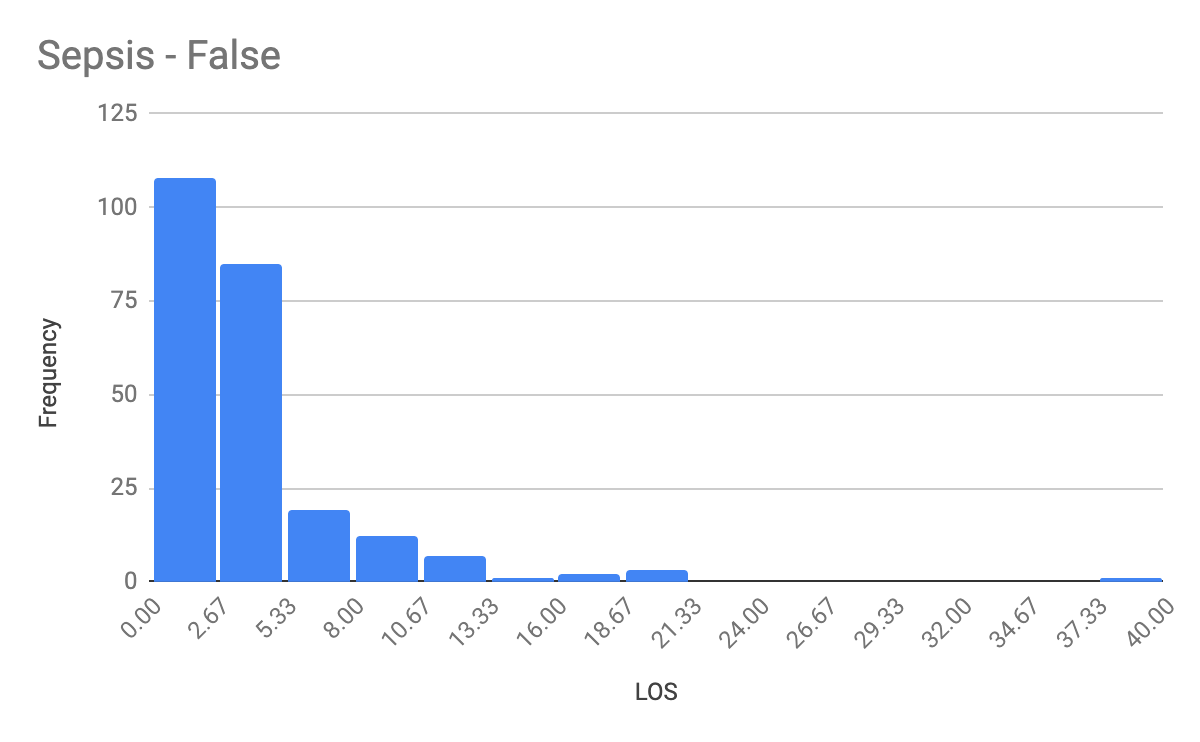


Figure S3 (g): Normal distribution of sepsis (false cases) of 34-37 weeks gestation


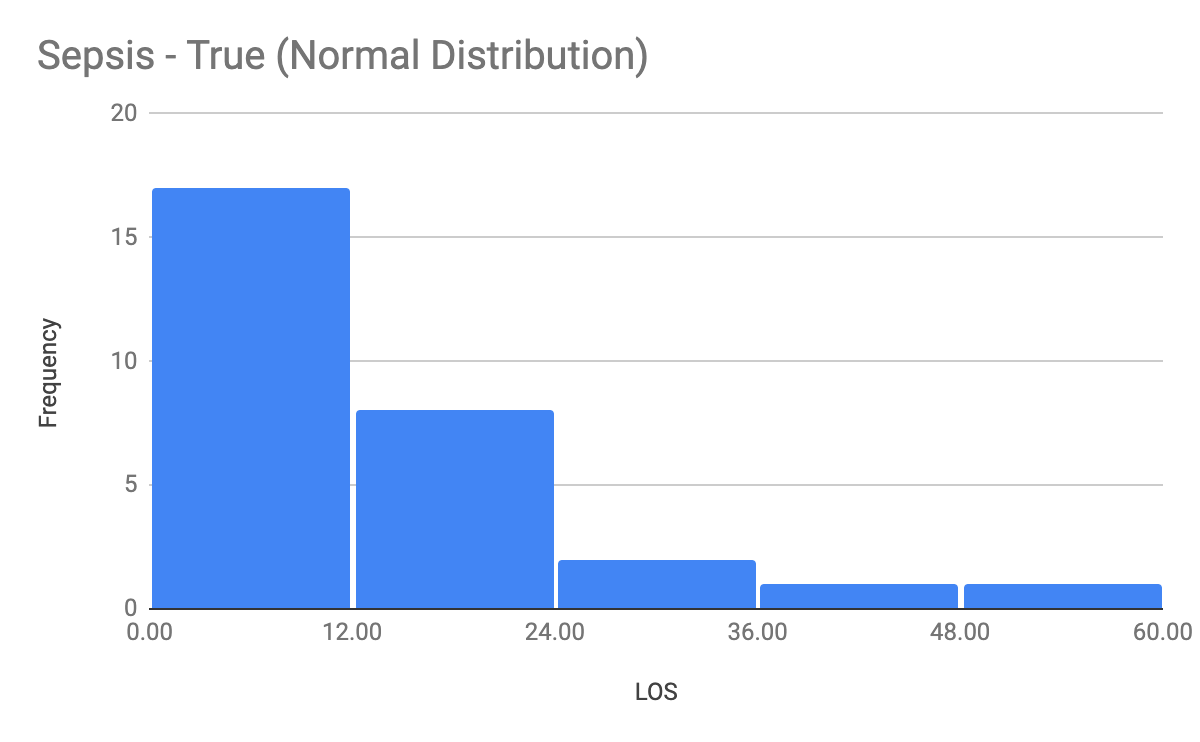


Figure S3 (h): Normal distribution of sepsis (true cases) of 34-37 weeks gestation


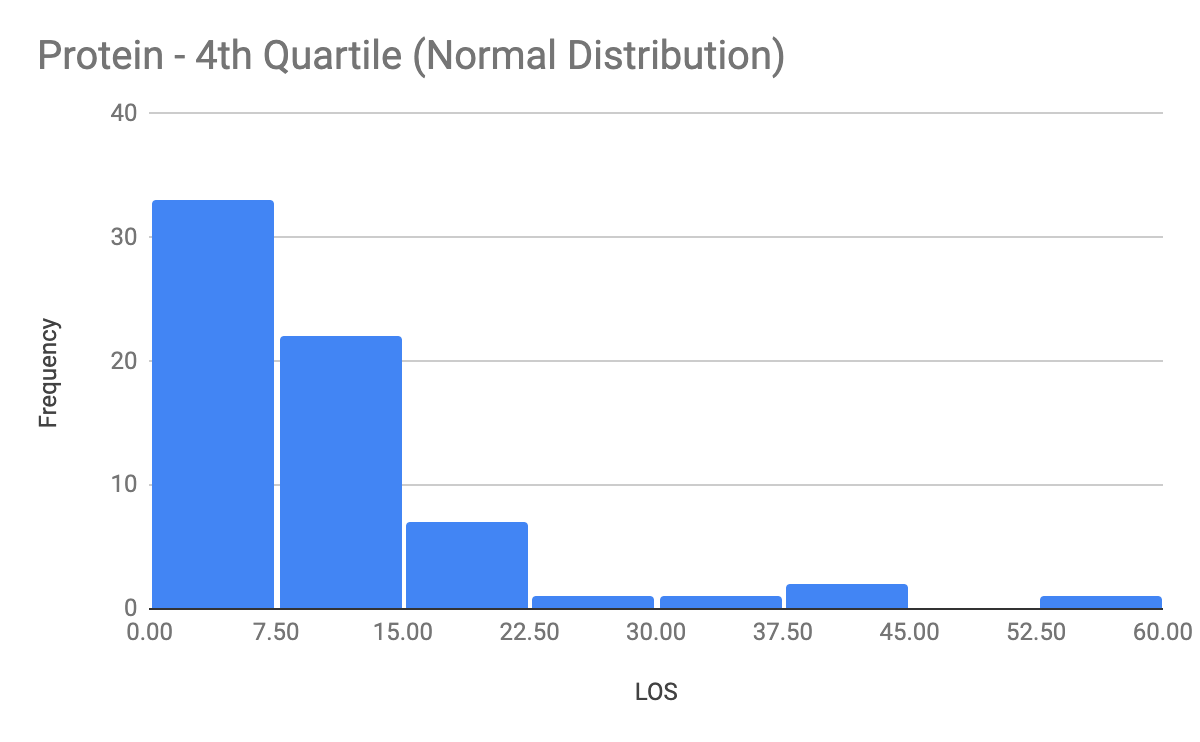


Figure S3 (i): Normal distribution of protein deviation (fourth quartile) of 34-37 weeks gestation


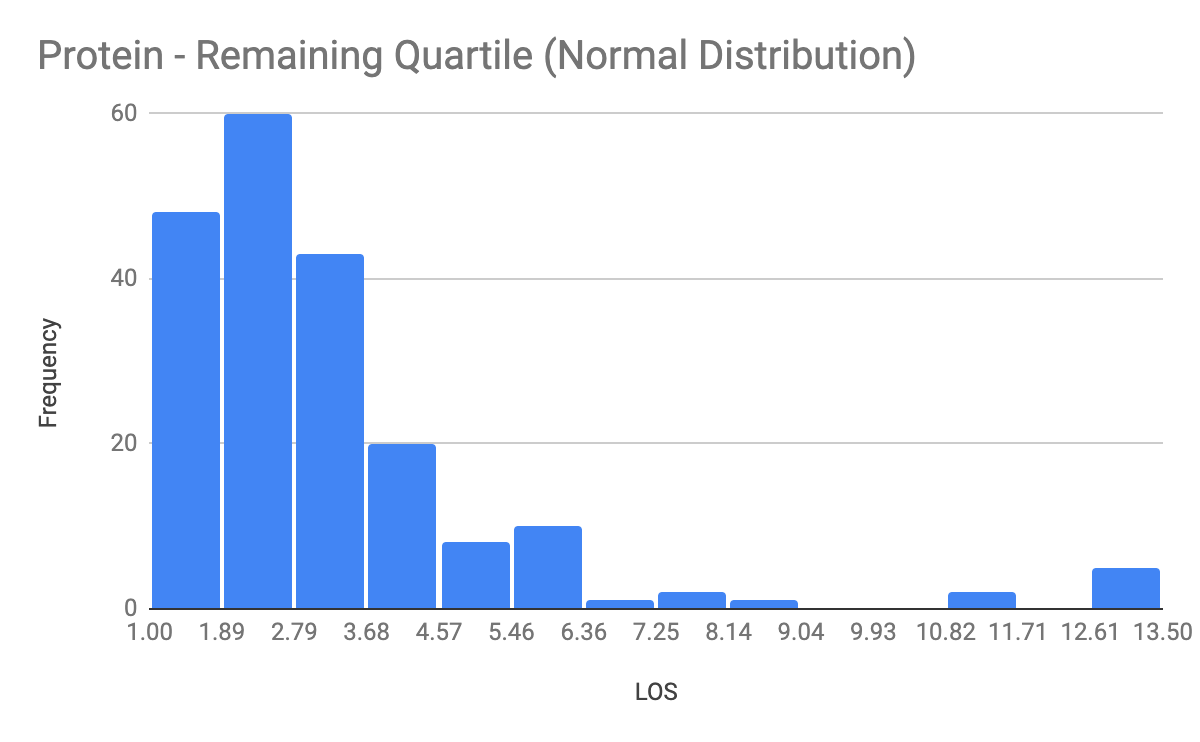


Figure S3 (j): Normal distribution of protein deviation (remaining quartile) of 34-37 weeks gestation


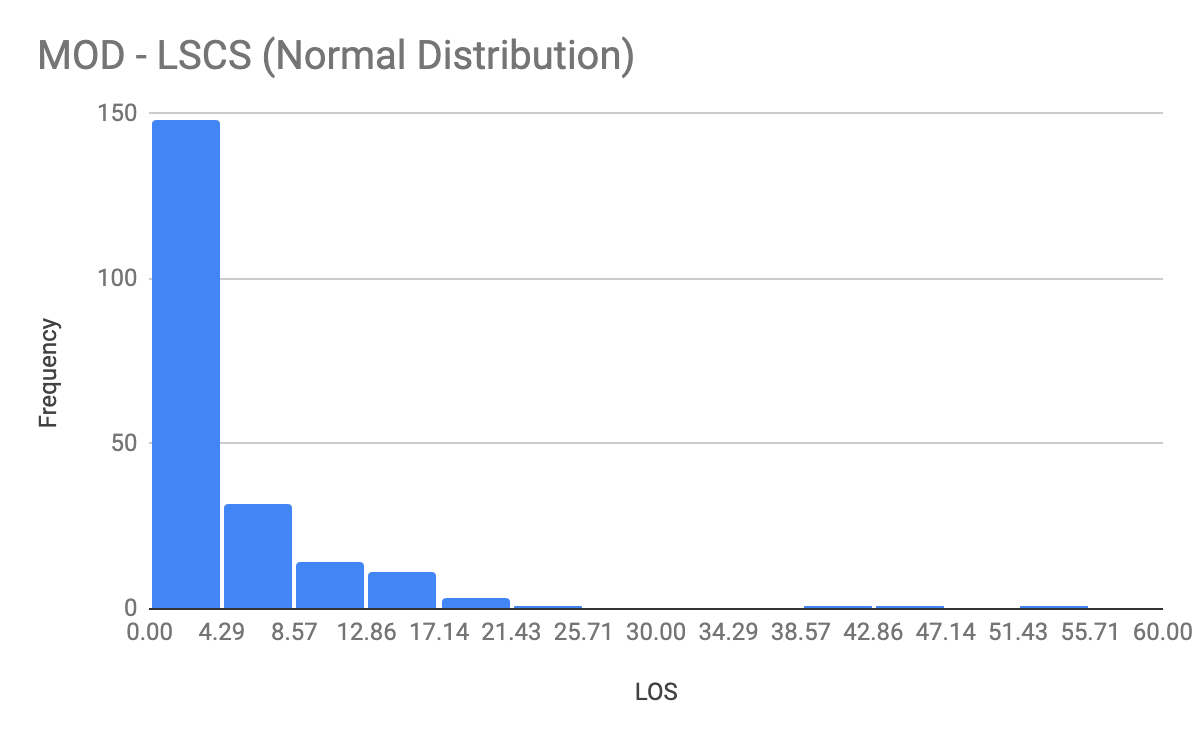


Figure S3 (k): Normal distribution of LSCS of 34-37 weeks gestation


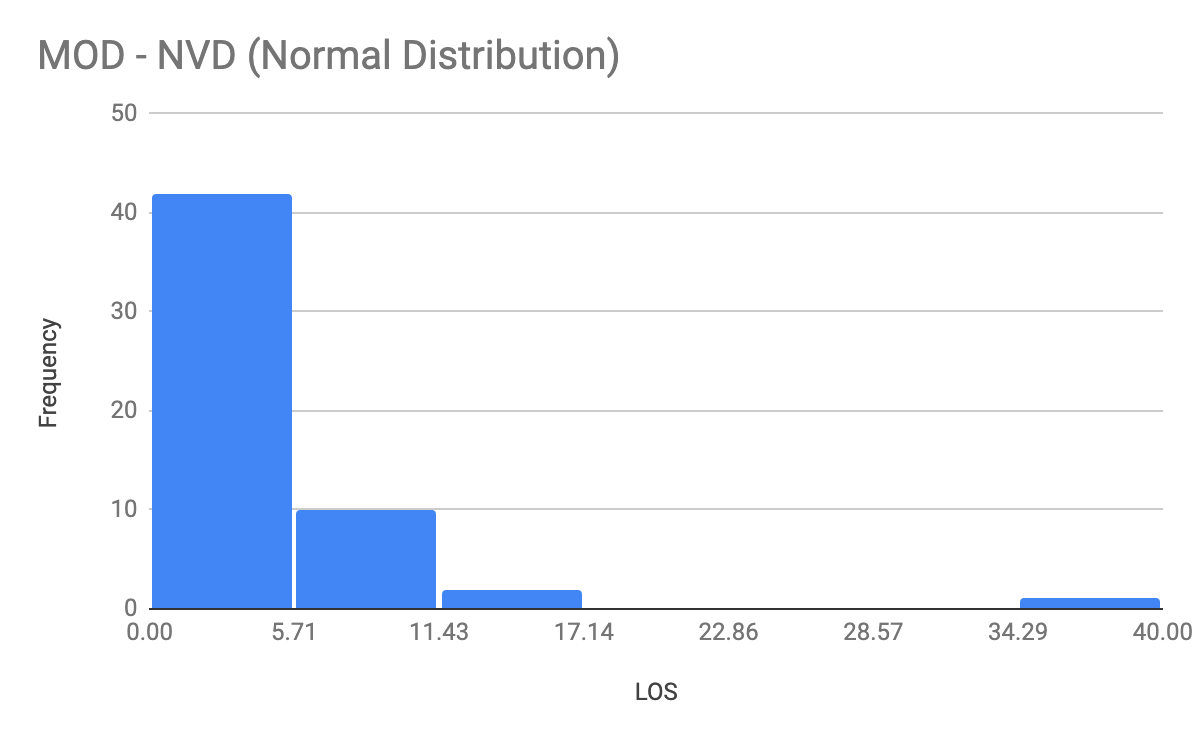


Figure S3 (l): Normal distribution of normal vaginal delivery of 34-37 weeks gestation


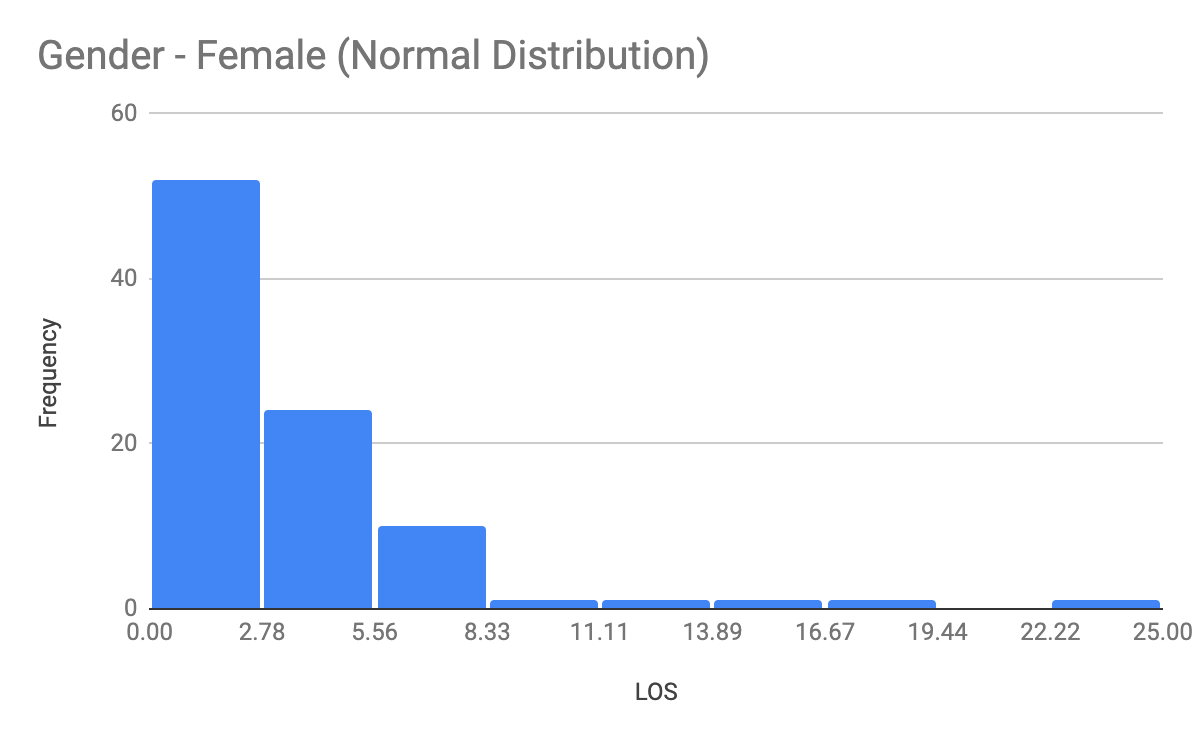


Figure S3 (m): Normal distribution of female of 34-37 weeks gestation


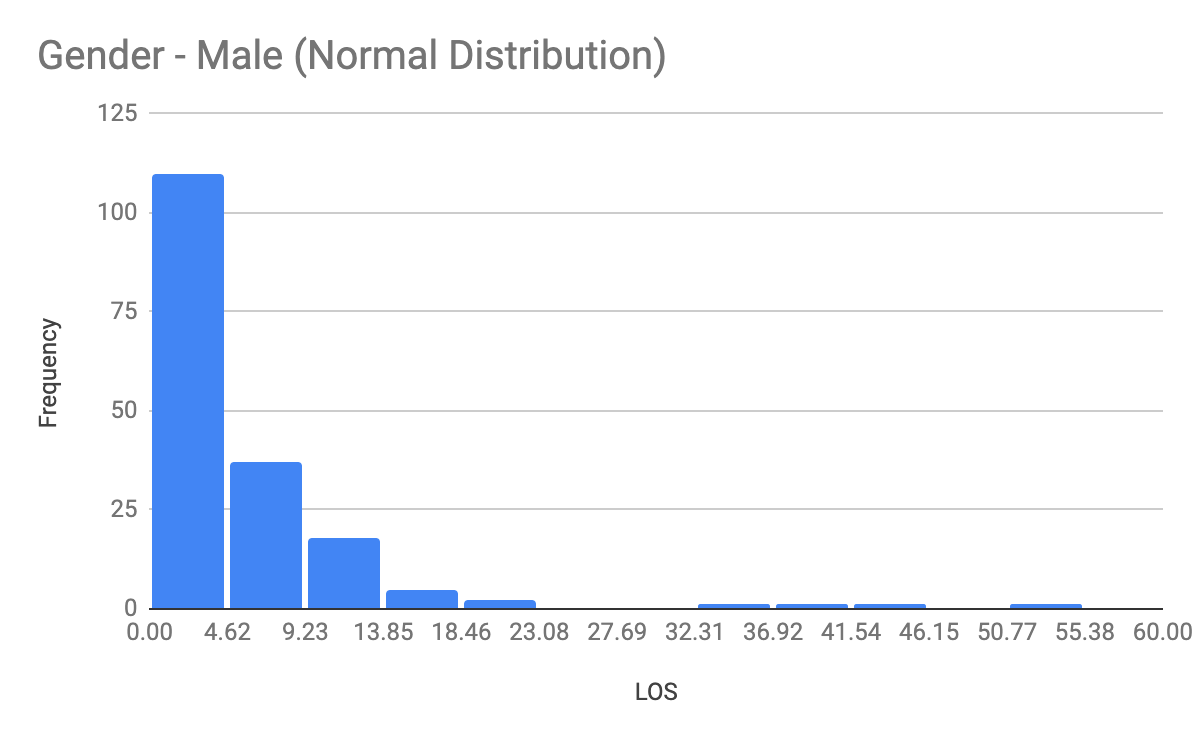


Figure S3 (n): Normal distribution of male of 34-37 weeks gestation

4. For > 37 weeks


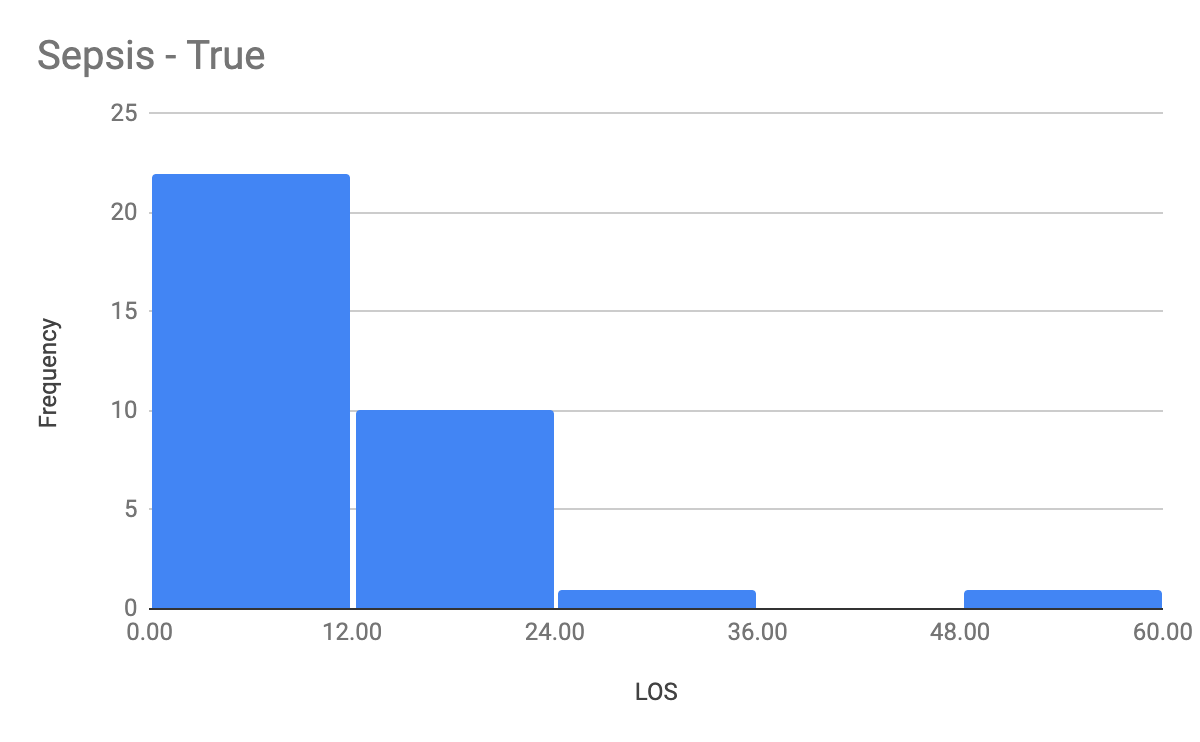


Figure S4 (a): Normal distribution of sepsis (true cases) of >37 weeks gestation


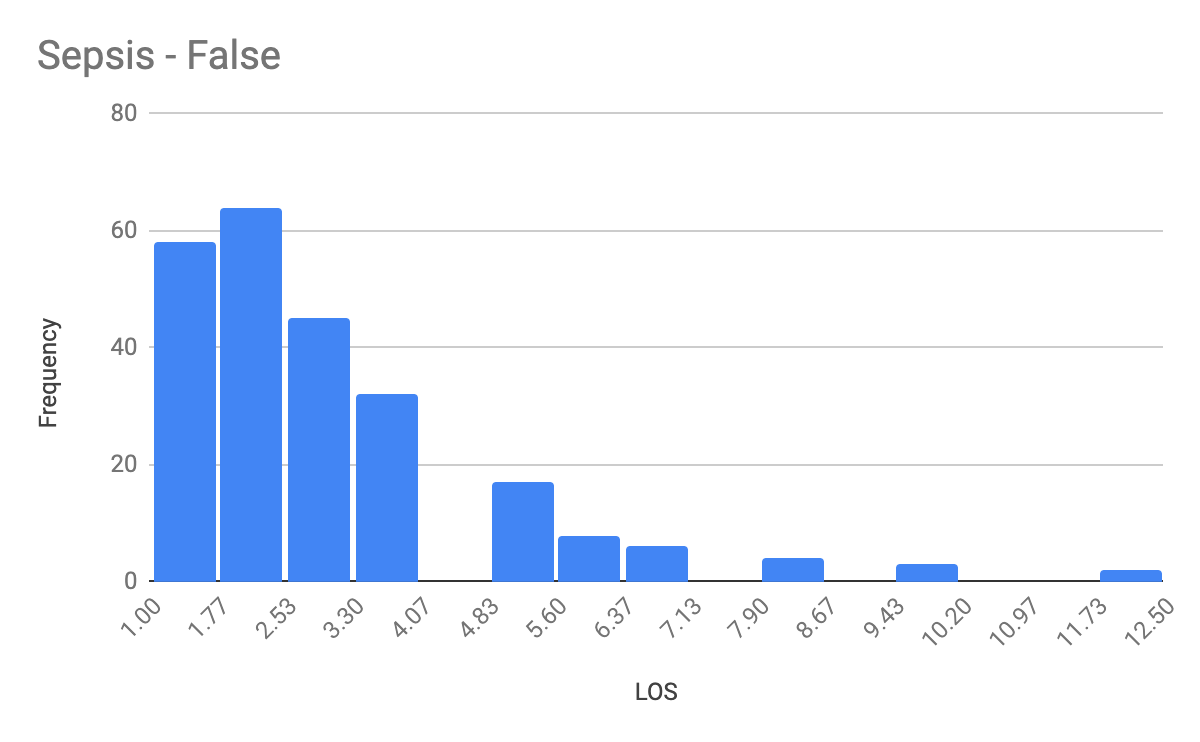


Figure S4 (b): Normal distribution of sepsis (false cases) of >37 weeks gestation


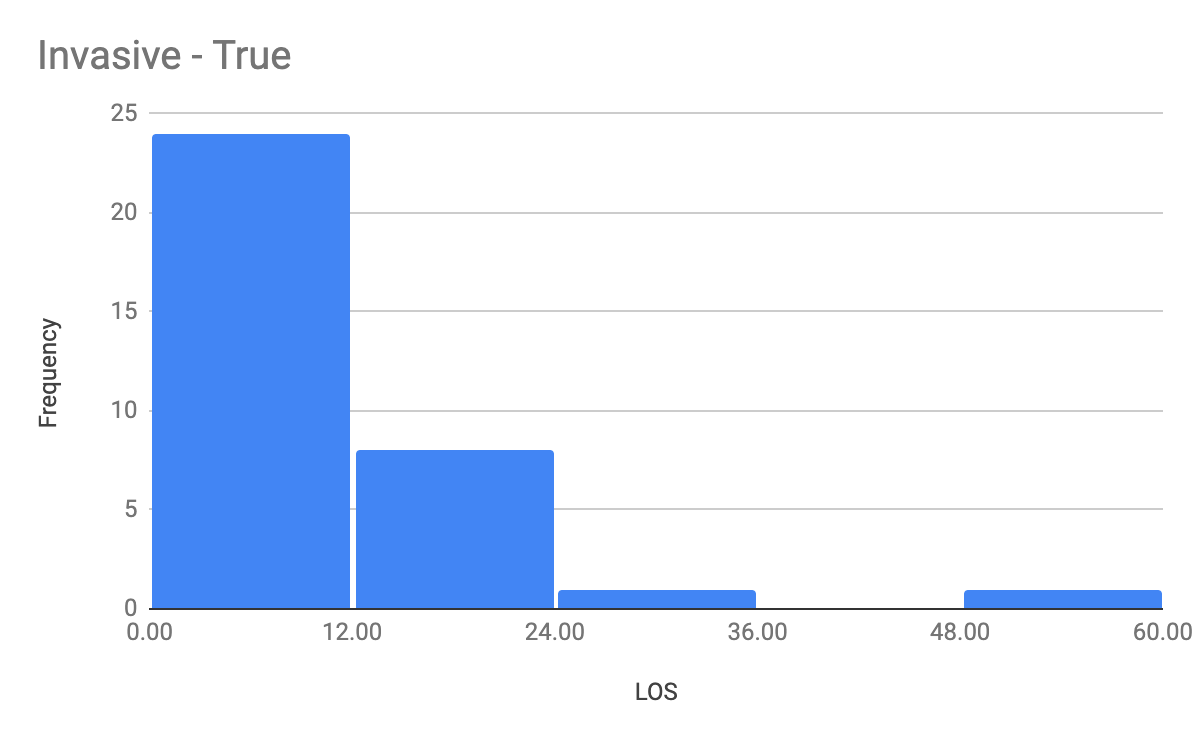


Figure S4 (c): Normal distribution of invasive (true cases) of >37 weeks gestation


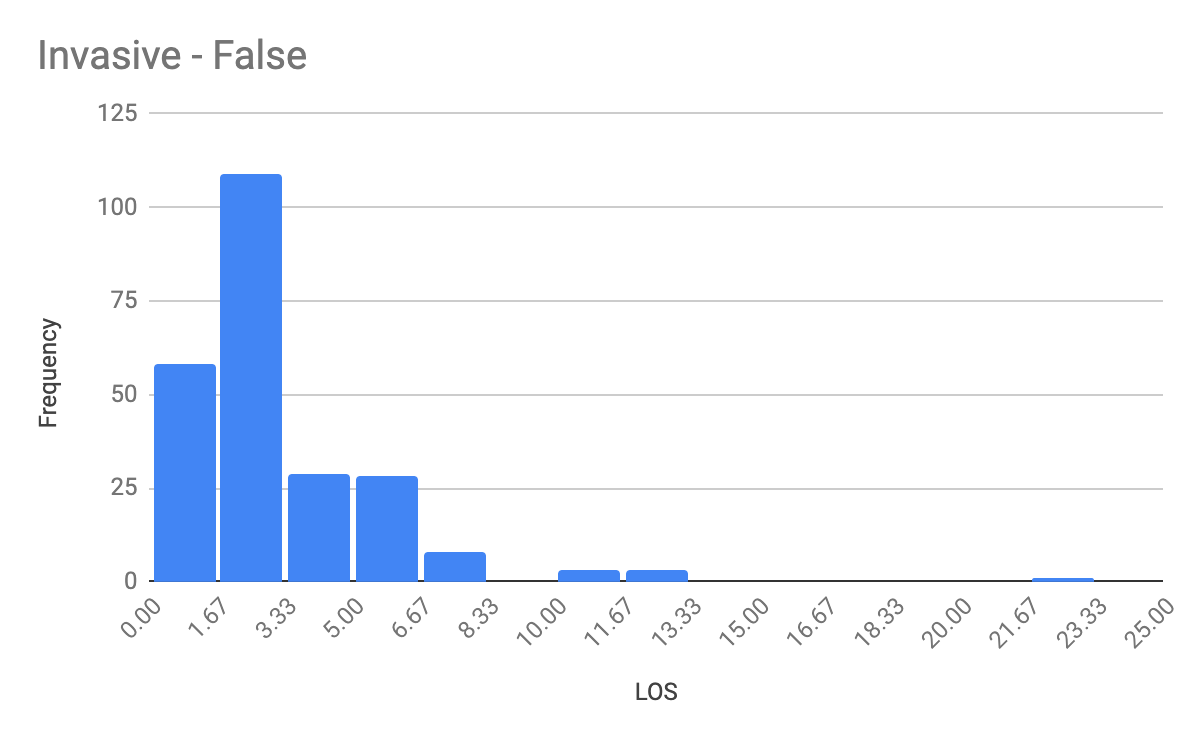


Figure S4 (d): Normal distribution of invasive (false cases) of >37 weeks gestation


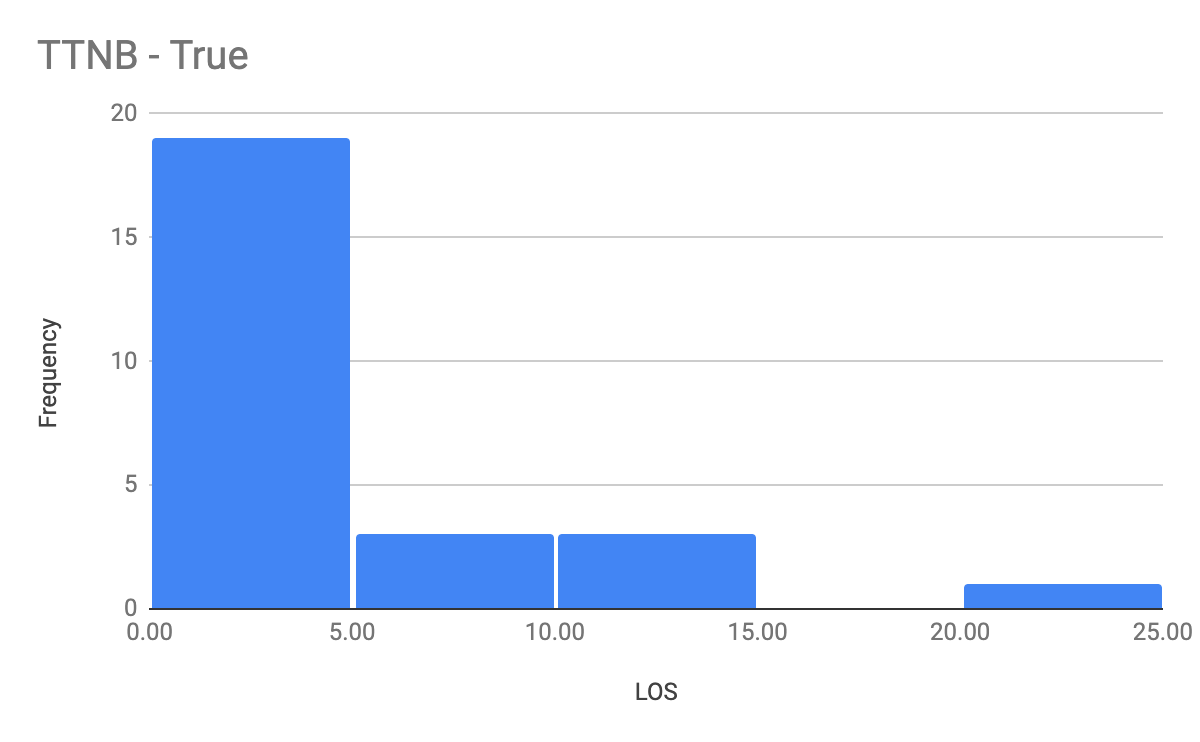


Figure S4 (e): Normal distribution of TTNB (true cases) of >37 weeks gestation


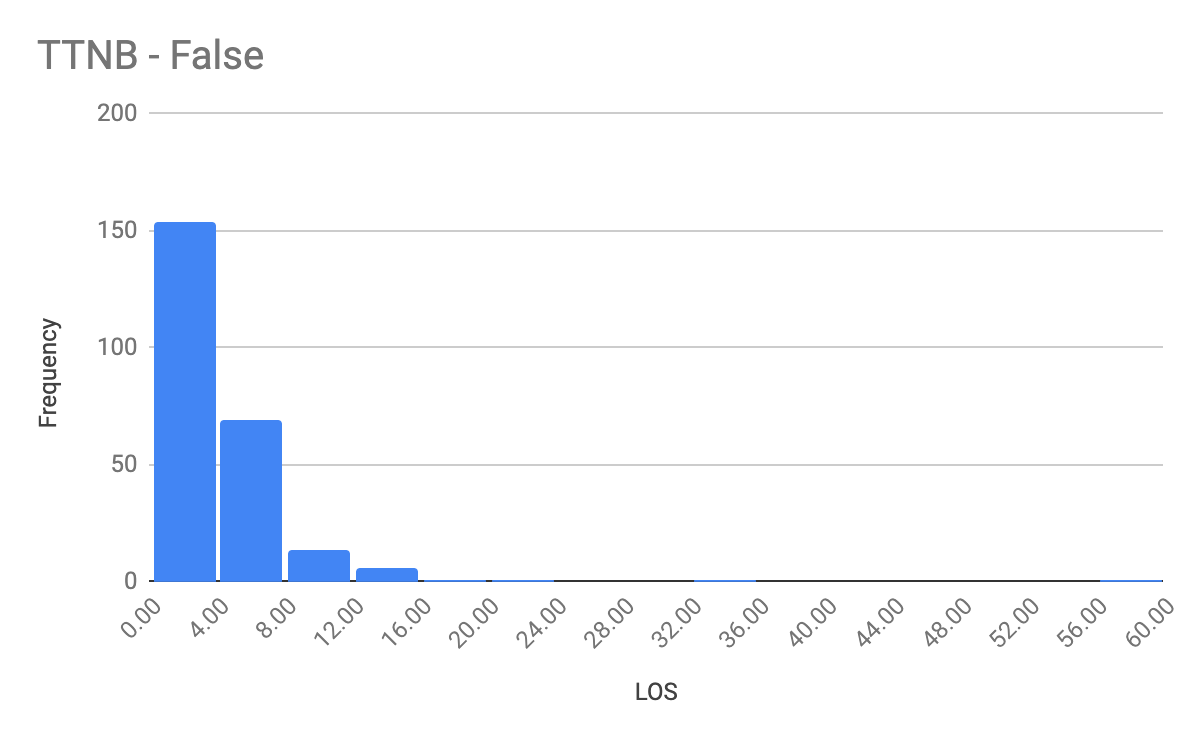


Figure S4 (f): Normal distribution of TTNB (false cases) of >37 weeks gestation


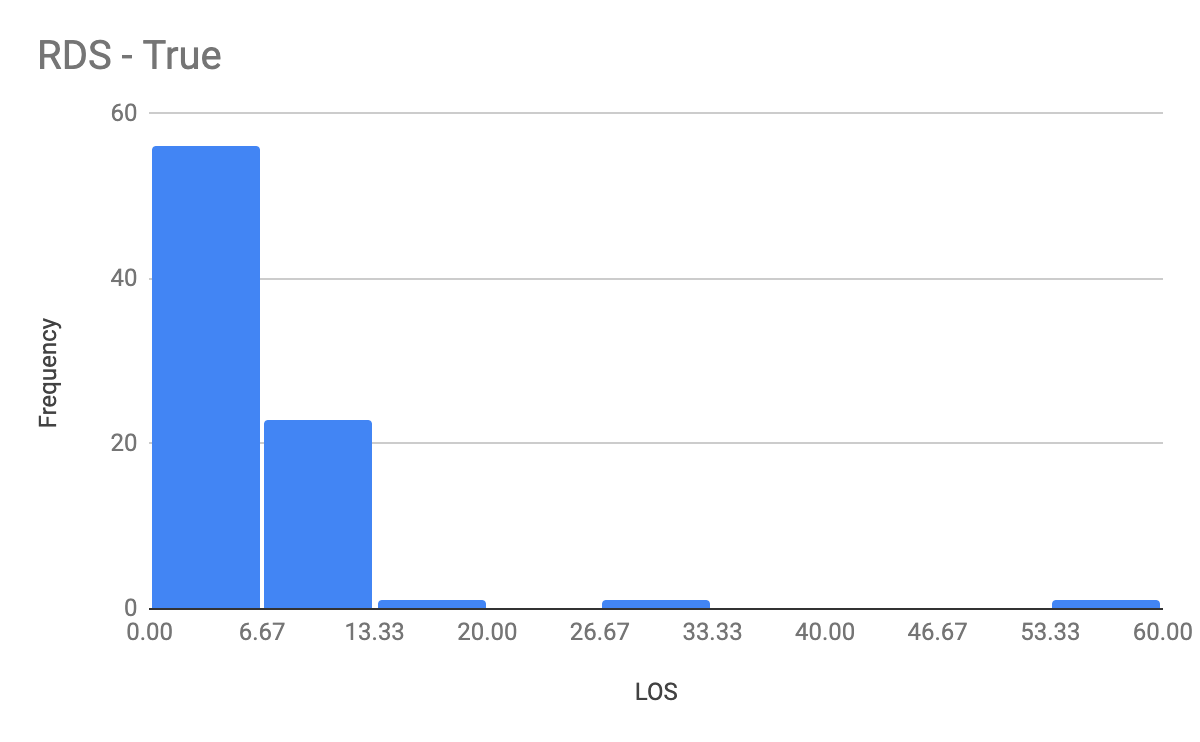


Figure S4 (g): Normal distribution of RDS (true cases) of >37 weeks gestation


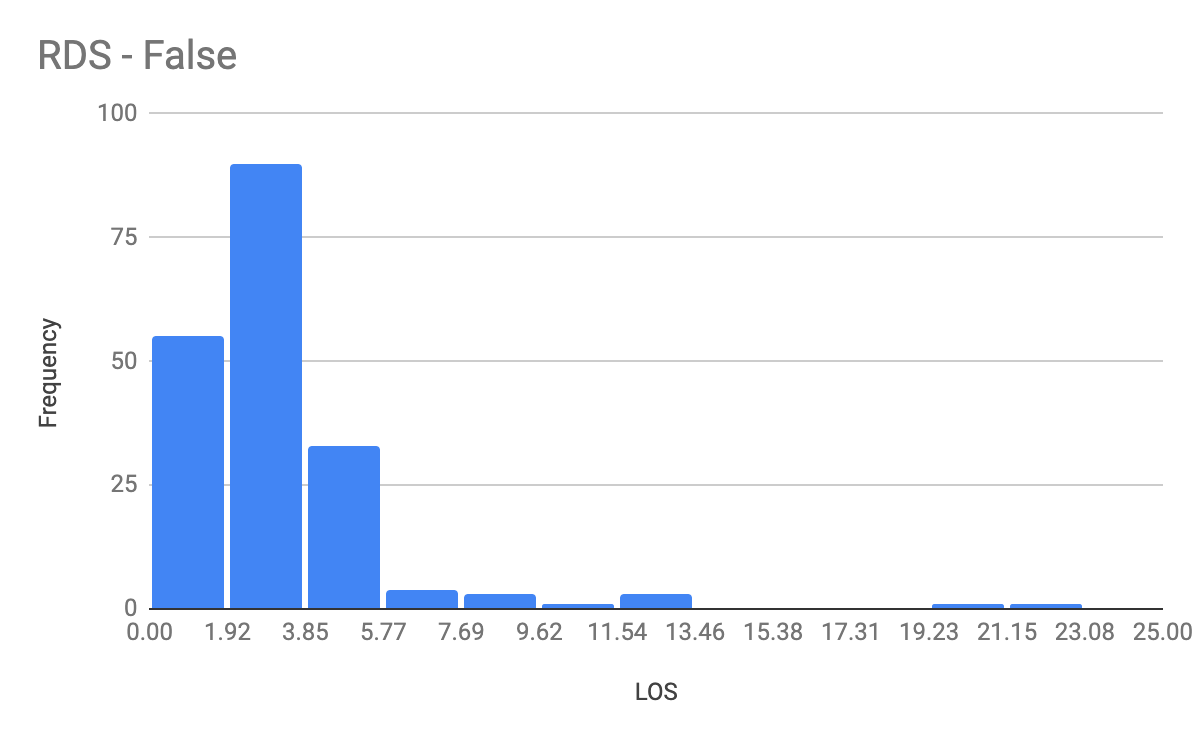


Figure S4 (h): Normal distribution of RDS (false cases) of >37 weeks gestation


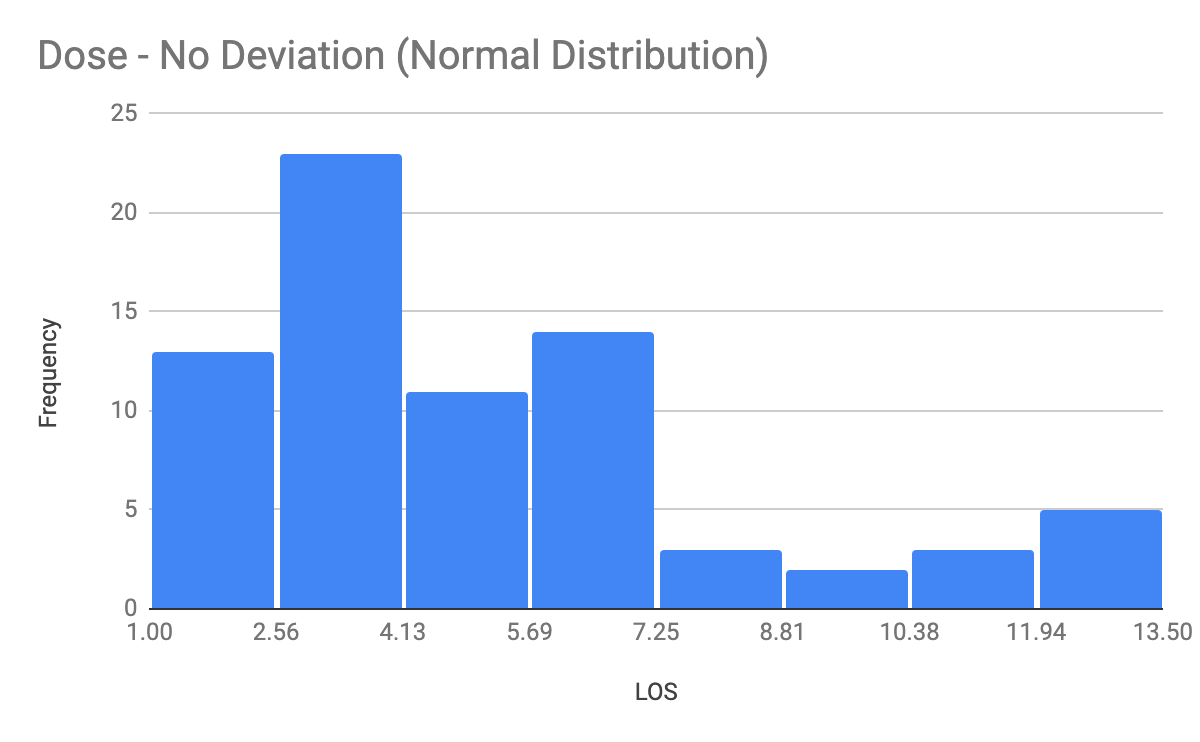


Figure S4 (i): Normal distribution of medication dose (no deviation) of >37 weeks gestation


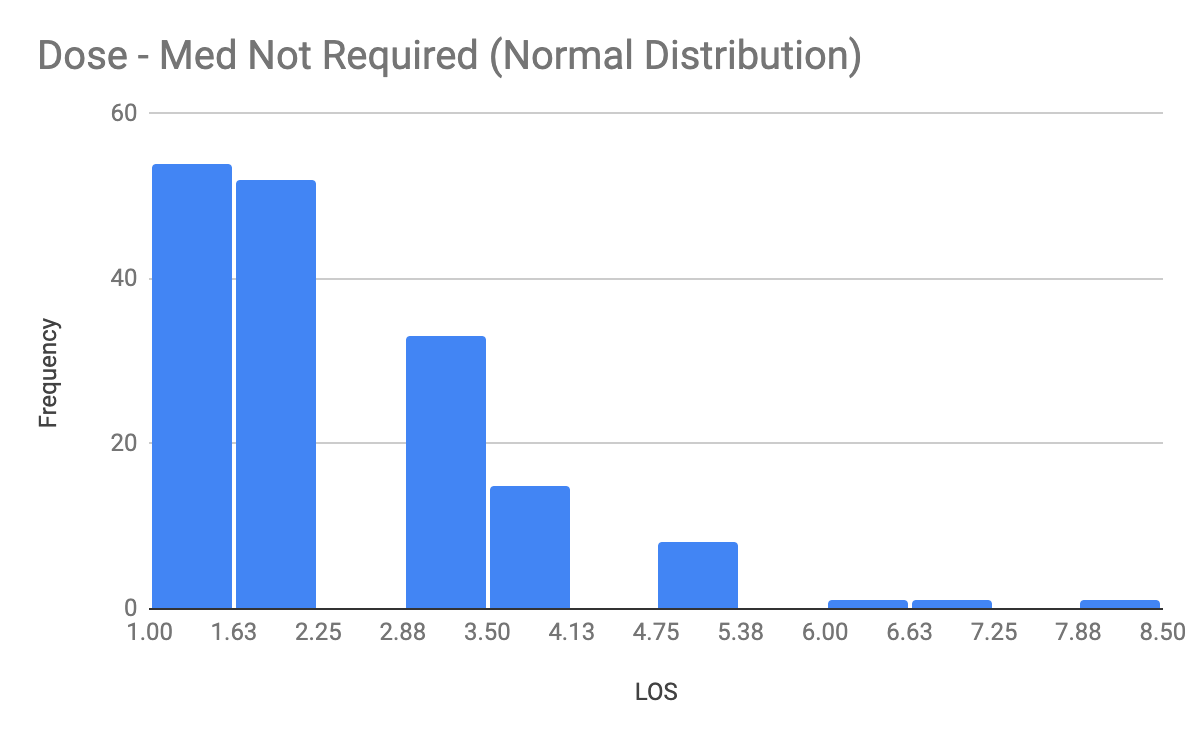


Figure S4 (j): Normal distribution of medication dose (medication not required) of >37 weeks gestation


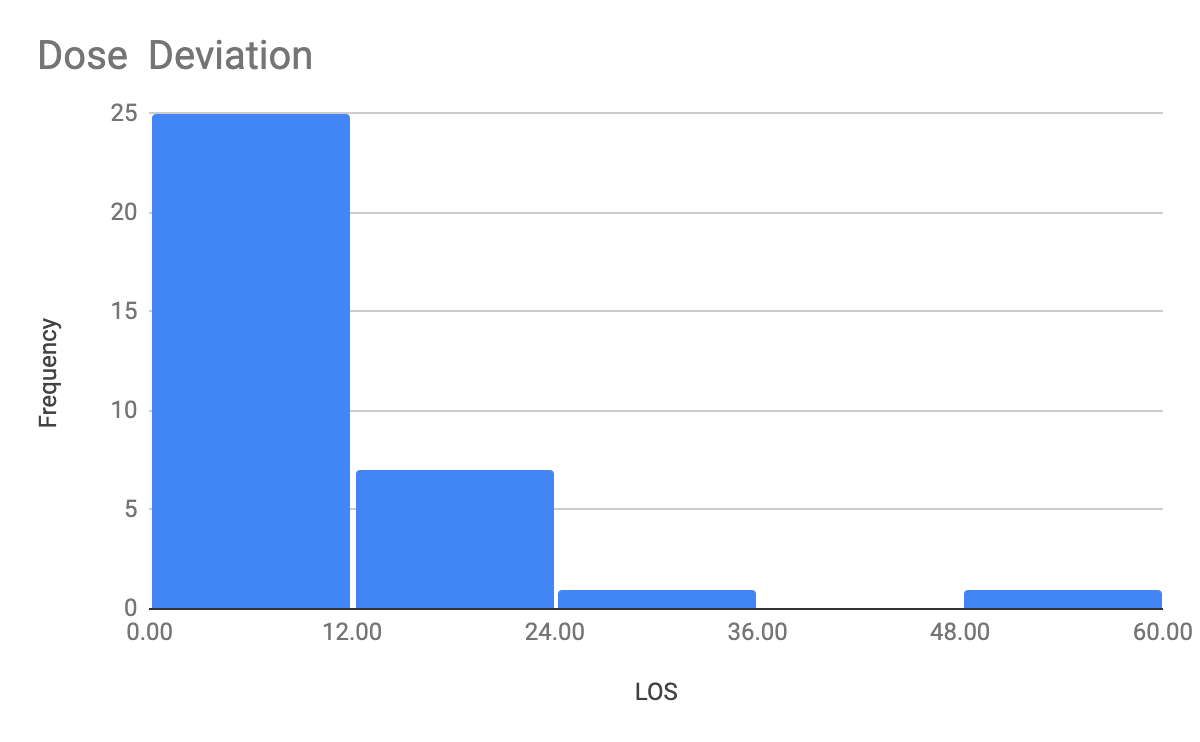


Figure S4 (k): Normal distribution of medication dose (deviation) of >37 weeks gestation


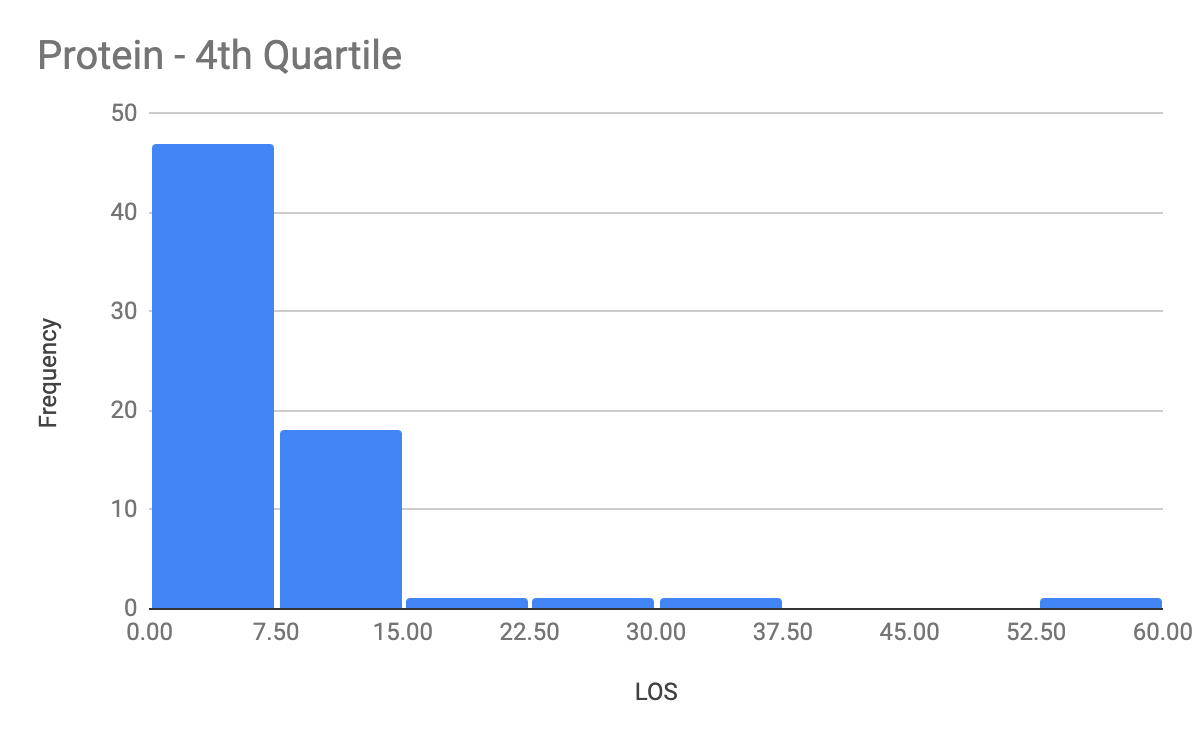


Figure S4 (l): Normal distribution of protein deviation (fourth quartile) of >37 weeks gestation


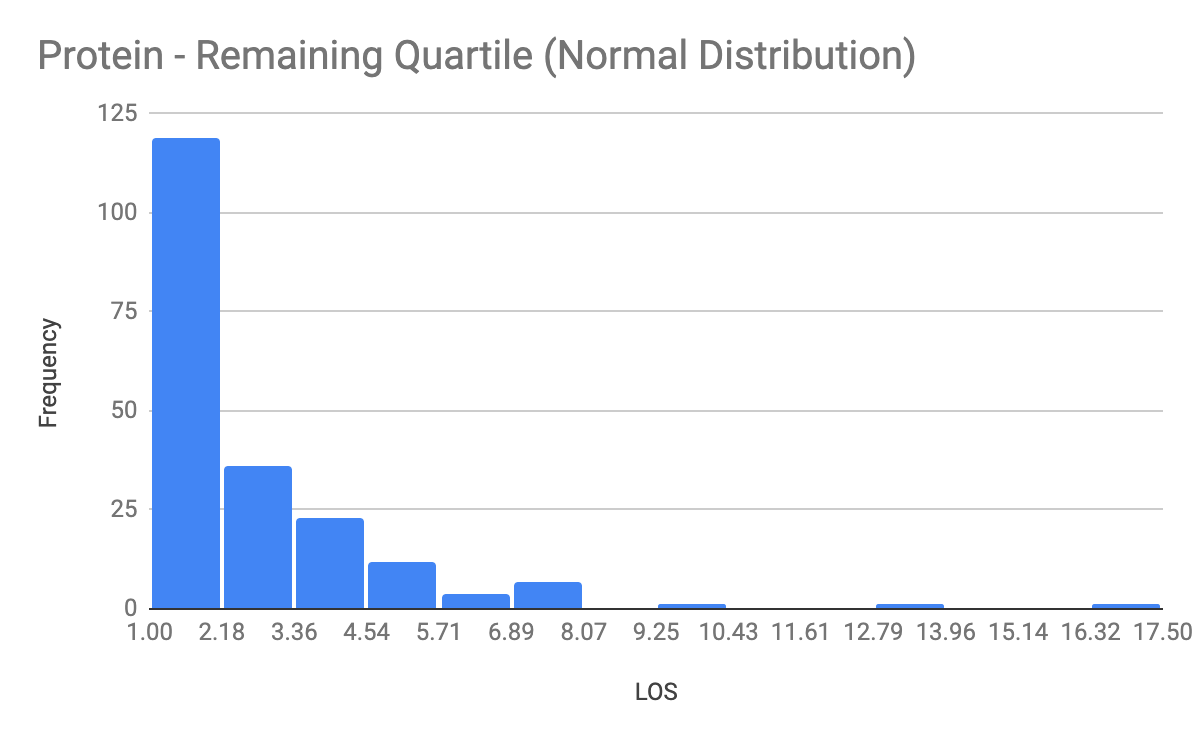


Figure S4 (m): Normal distribution of protein deviation (remaining quartile) of >37 weeks gestation

**
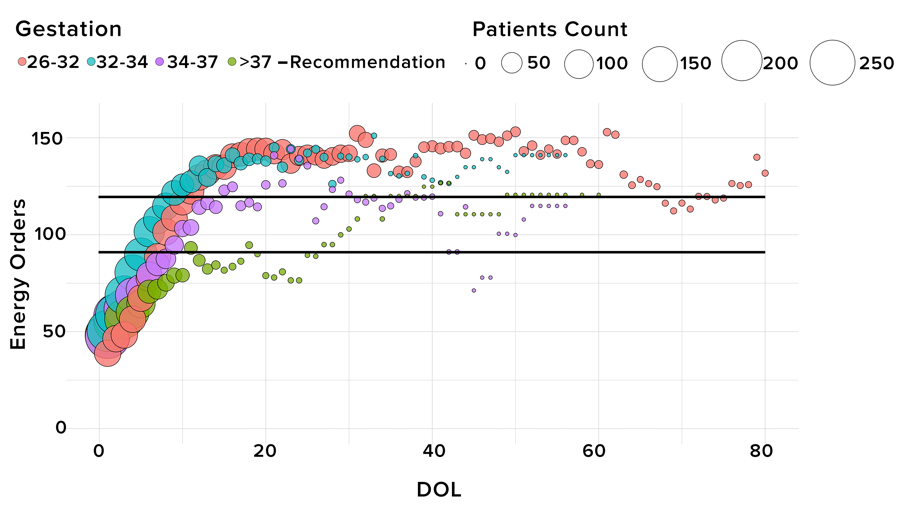
**

Figure S5 (a): Intake in energy orders across gestational age categories- bubble plot


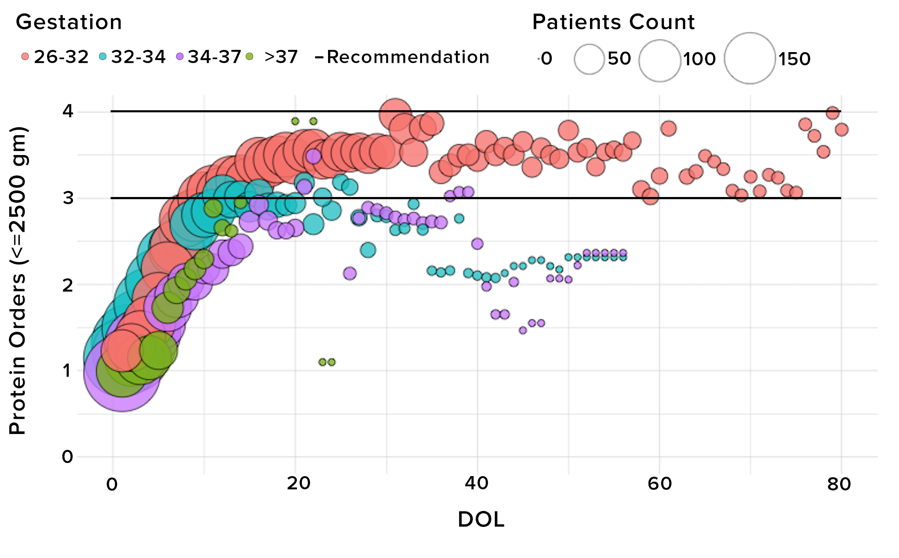


Figure S5 (b): Protein intake of patients with birth weight <=2500 gram


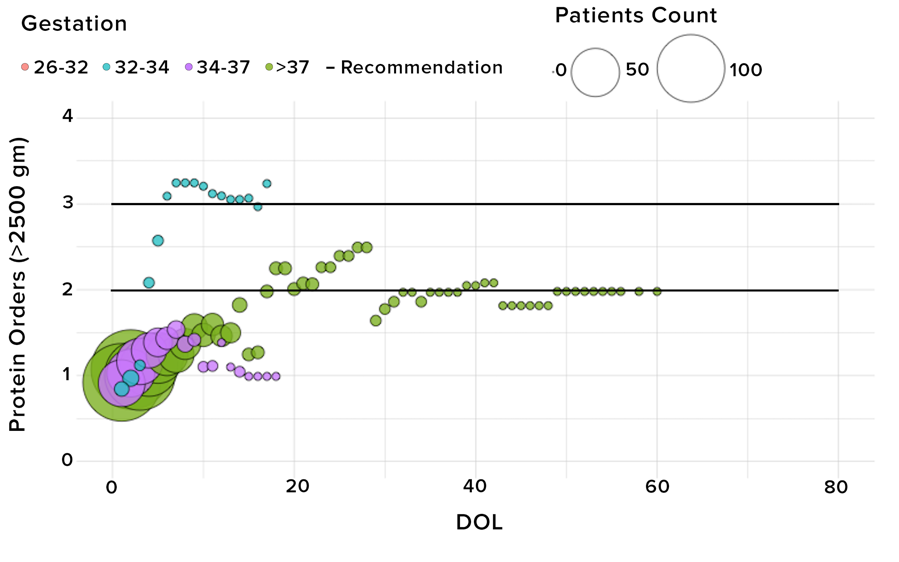


Figure S5(c): Protein intake of patients with birth weight > 2500 gram


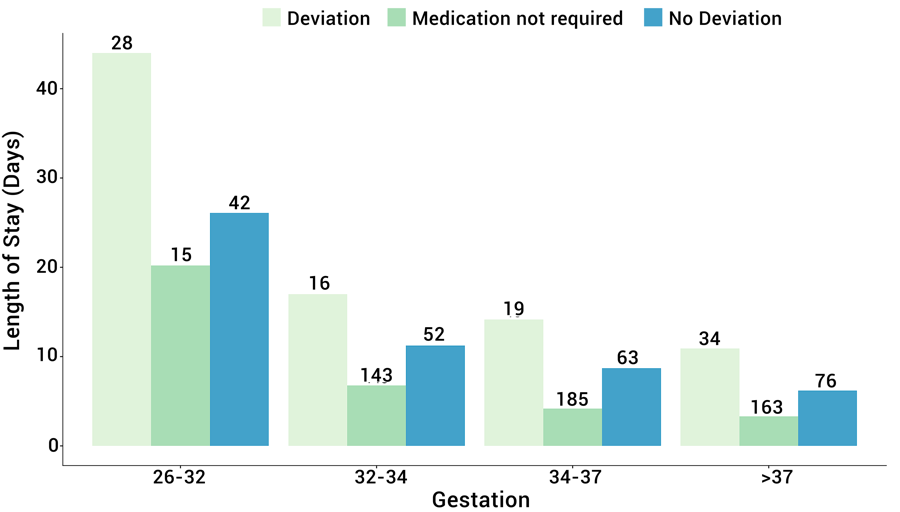


Figure S6 (a): Medicine deviation (dosage) across gestational age groups (Numbers mentioned on bars denote the “Count of patients”)


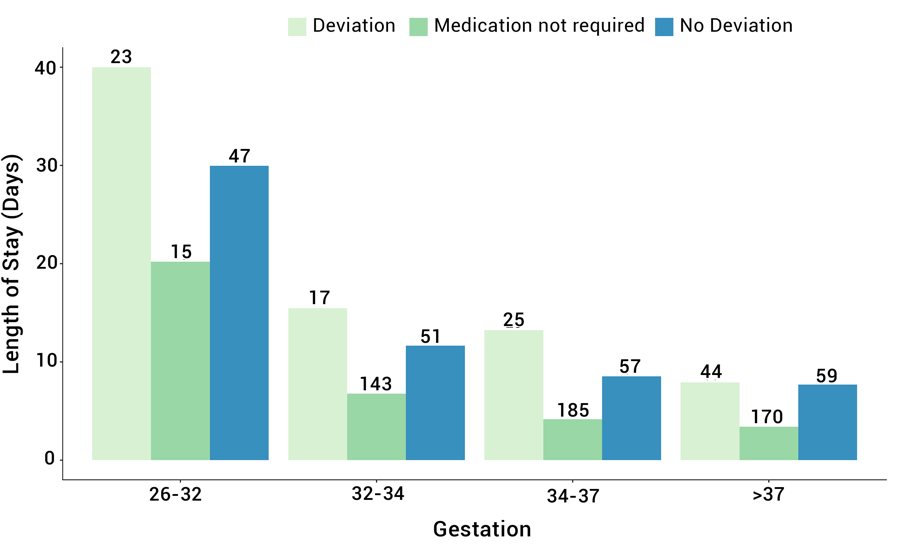


Figure S6 (b): Medicine deviation (frequency) across gestational age groups (Numbers mentioned on bars denote the “Count of patients”)


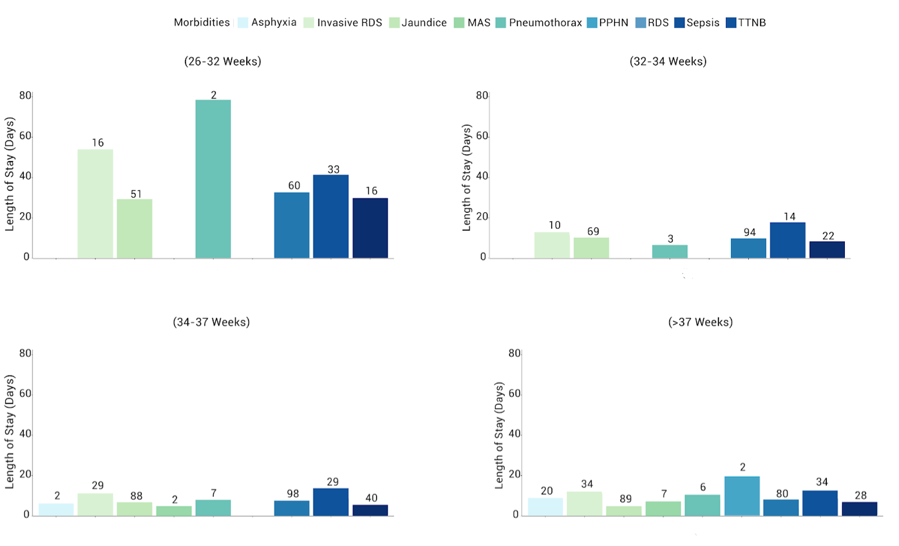
 Figure S7: Clinical diagnosis distribution across gestational age groups (Numbers mentioned on bars denote the “Patient counts”)


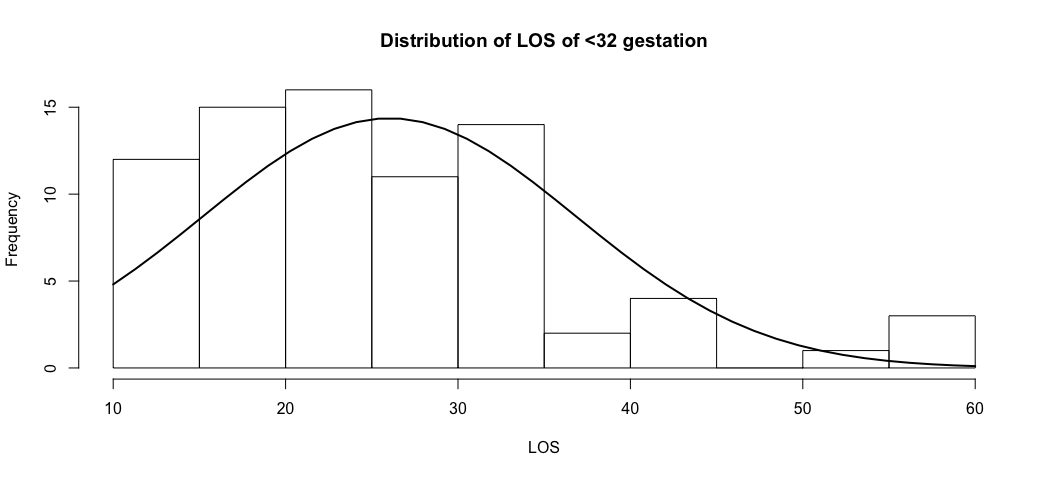


Figure S8 (a): 26-32 weeks’ (Patient frequency vs LOS) distribution


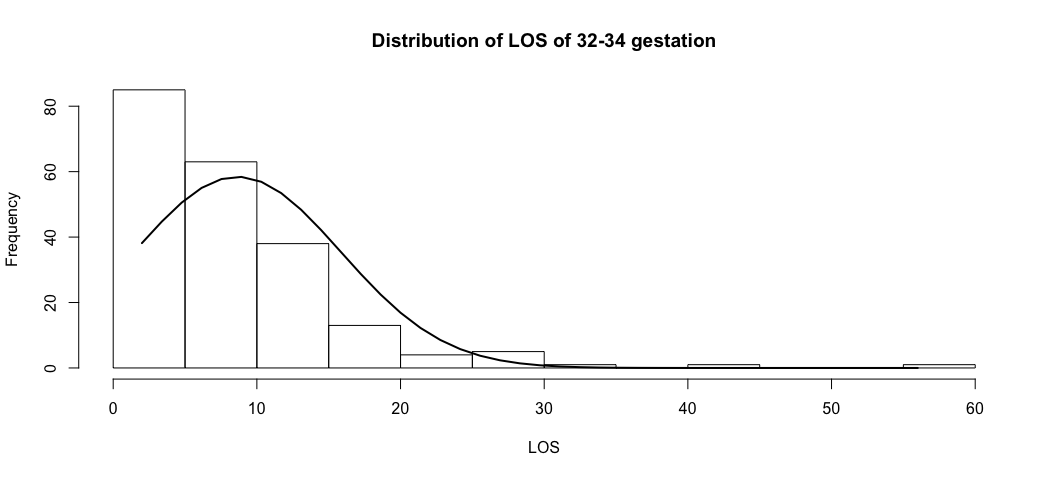


Figure S8 (b): 32-34 weeks’ (Patient frequency vs LOS) distribution


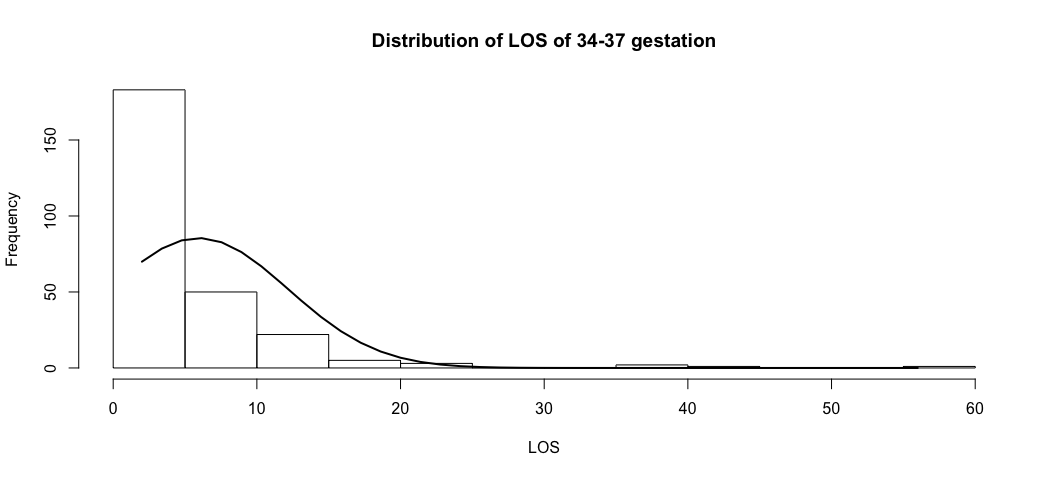


Figure S8 (c): 34-37 weeks’ (Patient frequency vs LOS) distribution


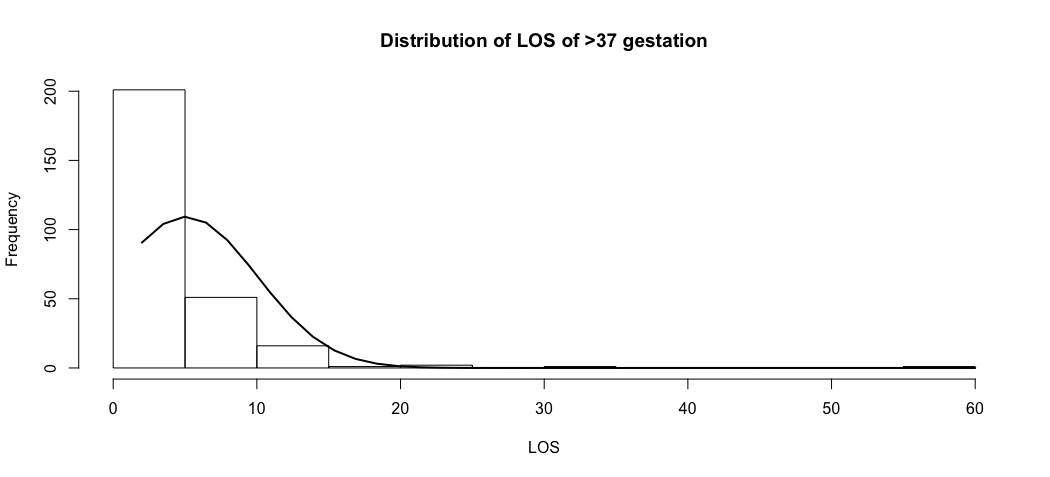


Figure S8 (d): >37 weeks’ (Patient frequency vs LOS) distribution


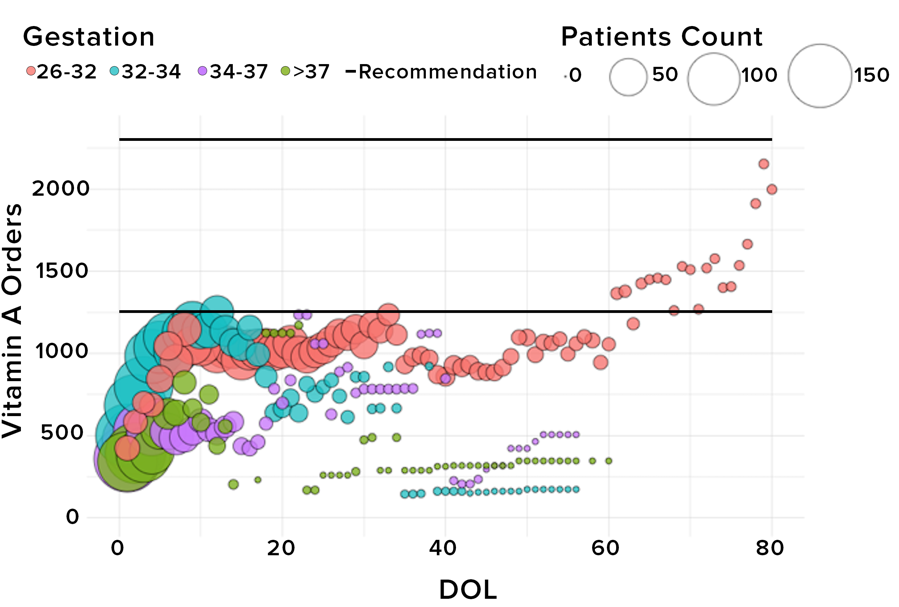


Figure S9 (a): Intake in Vitamin A orders across gestation categories – bubble plot


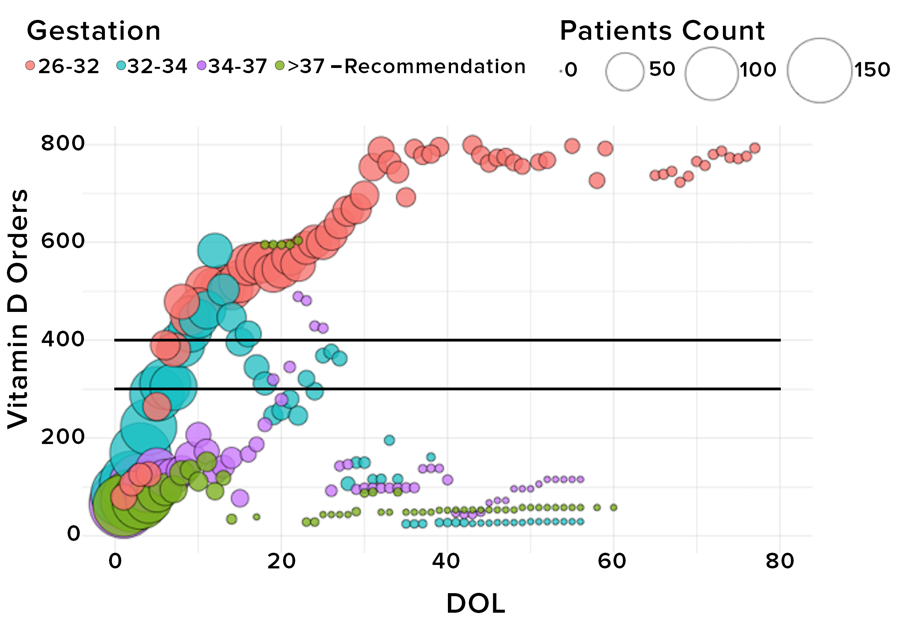


Figure S9 (b): Intake in Vitamin D orders across gestation categories – bubble plot


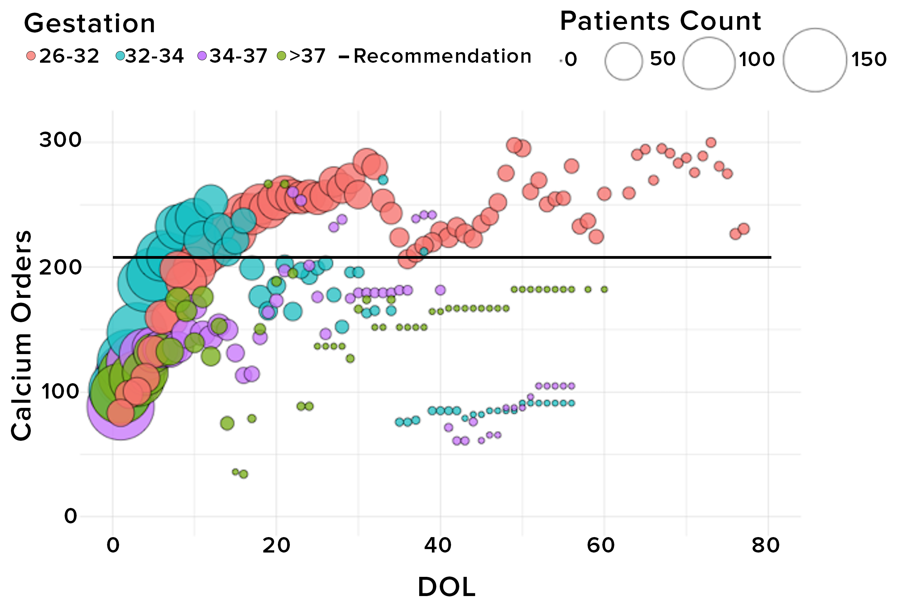


Figure S9 (c): Intake in Calcium orders across gestation categories – bubble plot


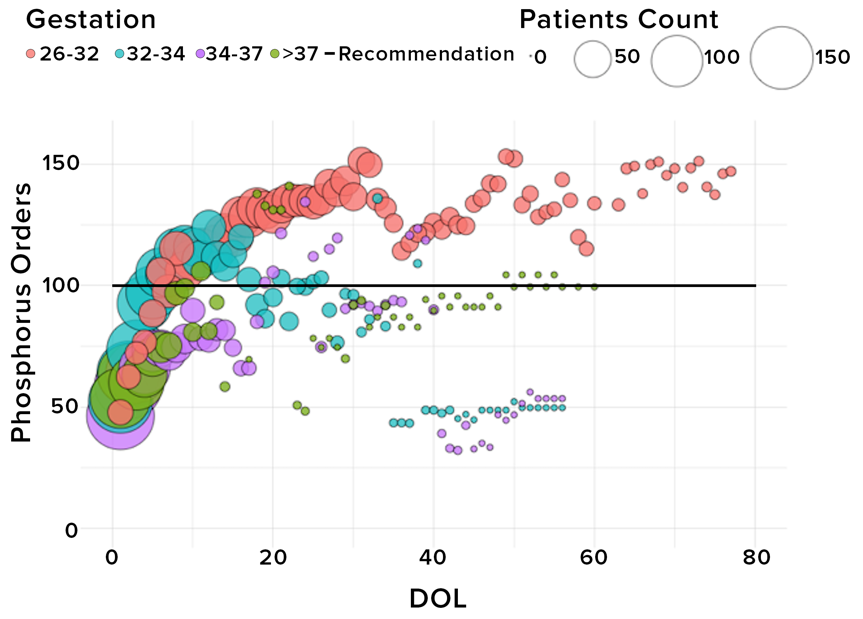


Figure S9 (d): Intake in Phosphorus orders across gestation categories – bubble plot


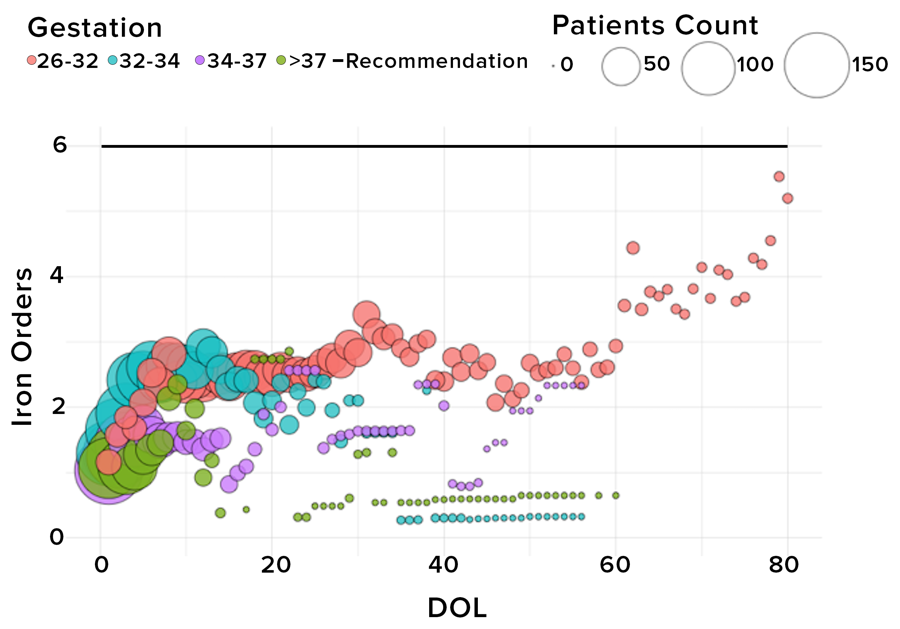


Figure S9 (e): Intake in Iron orders across gestation categories – bubble plot


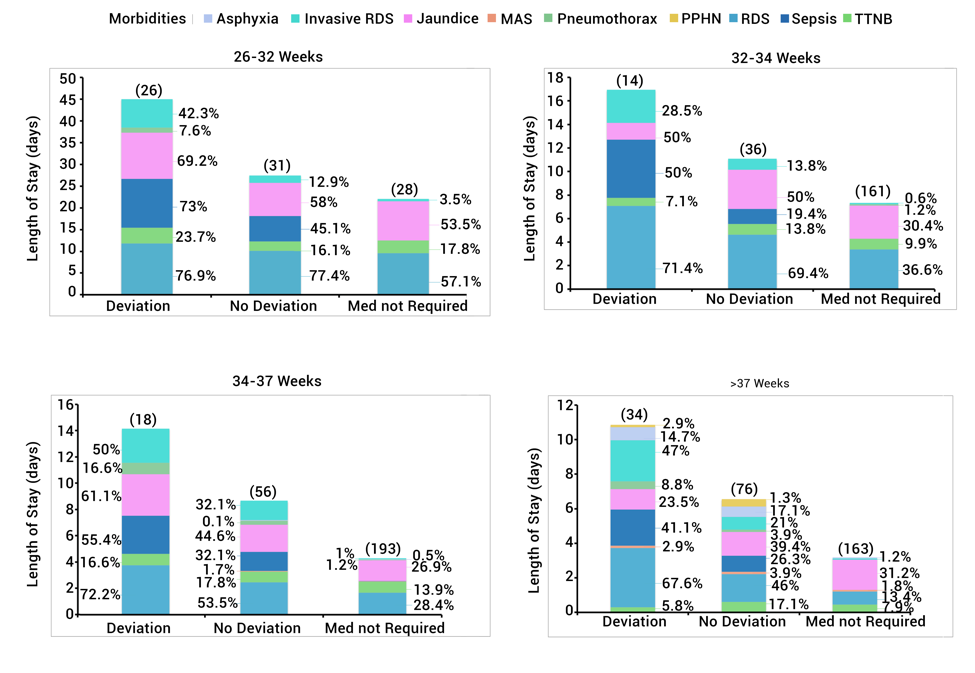


Figure S10: Clinical diagnosis distribution across gestation groups with medication deviation (Numbers mentioned on bars denote the “Count of patients”)

**Tables**

| **Table S1(a):** Observed and Predicted LOS (in days) for 26-32 Weeks | | | | |
| --- | --- | --- | --- | --- |
|  | 26-32 weeks | | | |
|  | Difference between Observed and predicted LOS | R squared | AIC | BIC |
| Antenatal and Perinatal Details | 0.8 | 86.4 | 20.19 | 31.9 |
| Antenatal, Perinatal and Diagnoses Details | 0.5 | 91.8 | 18 | 35.7 |
| Antenatal, Perinatal and Nutrition Details | 0.4 | 88.5 | 20.1 | 34.2 |
| Antenatal, Perinatal and Medication Details | 0.5 | 90 | 16.8 | 31 |
| Antenatal, Perinatal, Nutrition and Diagnoses Details | 0.3 | 93.1 | 18.03 | 38.05 |
| Antenatal, Perinatal, Medication and Diagnoses Details | 0.3 | 92.7 | 19.3 | 39.3 |
| Antenatal, Perinatal, Nutrition, Medication and Diagnoses Details | 0.4 | 93.7 | 19.5 | 41.8 |

| **Table S1(b):** Observed and Predicted LOS (in days) for 32-34 Weeks | | | | |
| --- | --- | --- | --- | --- |
|  | 32-34 weeks | | | |
|  | Difference between Observed and predicted LOS | R squared | AIC | BIC |
| Antenatal and Perinatal Details | 1.6 | 21.5 | 136.2 | 157.2 |
| Antenatal, Perinatal and Diagnoses Details | 1.2 | 43.7 | 125.8 | 157.5 |
| Antenatal, Perinatal and Nutrition Details | 1 | 34.3 | 129.2 | 154.5 |
| Antenatal, Perinatal and Medication Details | 0.9 | 50.6 | 111.8 | 137.1 |
| Antenatal, Perinatal, Nutrition and Diagnoses Details | 0.7 | 54.1 | 117.4 | 153.3 |
| Antenatal, Perinatal, Medication and Diagnoses Details | 1.7 | 57.9 | 112.1 | 126.3 |
| Antenatal, Perinatal, Nutrition, Medication and Diagnoses Details | 0.5 | 63.9 | 106.7 | 146.8 |

| **Table S1(c):** Observed and Predicted LOS (in days) for 34-37 weeks | | | | |
| --- | --- | --- | --- | --- |
|  | 34-37 weeks | | | |
|  | Difference between Observed and predicted LOS | R squared | AIC | BIC |
| Antenatal and Perinatal Details | 0.8 | 7.8 | 135.5 | 156.7 |
| Antenatal, Perinatal and Diagnoses Details | 0.7 | 23.1 | 138.2 | 174.3 |
| Antenatal, Perinatal and Nutrition Details | 0.4 | 17.4 | 132.6 | 158.1 |
| Antenatal, Perinatal and Medication Details | 0.7 | 27.9 | 124.2 | 149.7 |
| Antenatal, Perinatal, Nutrition and Diagnoses Details | 0.4 | 28.2 | 138 | 178.4 |
| Antenatal, Perinatal, Medication and Diagnoses Details | 0.6 | 34.6 | 132.2 | 172.6 |
| Antenatal, Perinatal, Nutrition, Medication and Diagnoses Details | 0.4 | 38.9 | 131.9 | 176.5 |

| **Table S1(d):** Observed and Predicted LOS (in days) for >=37 Weeks | | | | |
| --- | --- | --- | --- | --- |
|  | >=37 weeks | | | |
|  | Difference between Observed and predicted LOS | R squared | AIC | BIC |
| Antenatal and Perinatal Details | 0.8 | 16.2 | 129.5 | 146.8 |
| Antenatal, Perinatal and Diagnoses Details | 0.5 | 54.9 | 105.9 | 140.4 |
| Antenatal, Perinatal and Nutrition Details | 0.6 | 32.8 | 119.4 | 141 |
| Antenatal, Perinatal and Medication Details | 0.2 | 48.2 | 102.7 | 124.3 |
| Antenatal, Perinatal, Nutrition and Diagnoses Details | 0.3 | 60.7 | 101 | 139.9 |
| Antenatal, Perinatal, Medication and Diagnoses Details | 1.1 | 57.6 | 105.8 | 144.7 |
| Antenatal, Perinatal, Nutrition, Medication and Diagnoses Details | 0.1 | 62.42 | 102.2 | 145.4 |

**Supplementary Method S2**

Steps involved in generating Table 2:

1. From birth, antenatal and perinatal details; several categorical risk factors were considered.

2. The different risk factors are:

(i) Gestational age – made categorical by subdividing gestation in equally distributed subgroups.

(ii) Gender – Male and Female.

(iii) Mode of Delivery – Vaginal (NVD) and Cesarean delivery (LSCS).

(iv) Birth status – Inborn and Out born.

(v) Pregnancy type – Single birth and multiple births (twins, triplets etc.)

(vi) Antenatal Steroids – Given (Yes) and Not given (No).

(vii) Antenatal diseases – Yes and No (Hypertension, Gestational Hypertension, Diabetes, Gestational diabetes mellitus, Chronic Kidney Disease, Hypothyroidism, Hyperthyroidism)

(viii) Antenatal infections – Yes and No (Fever, Urinary tract infection, History of intrauterine infections)

(ix) Antenatal risk factors – Yes and No (Premature Rupture Of Membrane, Preterm Premature Rupture Of Membrane, Prematurity, Chorioamniotis, Oligohydraminos, Polyhydraminos)

(x) Umbilical doppler – Normal, abnormal, unknown

(xi) Need for resuscitation – No, Initial steps, O2, PPV, Chest compression

(xii) APGAR (1 or 5 mins) – Greater than 5, Lesser than 5, unknown

(xiii) RDS – Presence (Yes) and absence (No)

(xiv) Severe RDS (invasive ventilation) – Presence (Yes) and absence (No)

(xv) Sepsis – Presence (Yes) and absence (No).

(xvi) Neonatal hyperbilirubinemia (NNH) - Presence (Yes) and absence (No).

(xvii) Pneumothorax – Presence (Yes) and absence (No).

(xviii) PPHN – Presence (Yes) and absence (No).

(xix) Asphyxia - Presence (Yes) and absence (No).

(xx) Medication dose deviation – No deviation, negative deviation or positive deviation.

(xxi) Nutrition deviation – Fourth quartile and rest three quartiles.

1. Energy
2. Protein

For each risk factor, a comparison between the factor and the median LOS of the group was done to conclude the extent of increase in number of days of stay as compared to the median LOS of the gestation group.

3. After the model was built, we used it to calculate Ls-means [27] for each categorical variable. To calculate Ls-means, we first created a reference grid. Reference grid is a regular grid of predictor settings. It has all possible combinations of independent variables. So, if there are 10 independent variables with each taking two possible values, then reference grid will have 210 vectors.

4. The regression model was applied on each vector in the reference grid to obtain the predicted LOS value. Confidence interval of 95% was used. Only those variables were displayed in the table that was found to be significant (p value< 0.05).

To calculate Ls-means of a particular value of an independent variable, we fixed that value in the reference grid and found the average of the predicted LOS values on all other factors. The least squared means of all variables are rounded to integer values and compared to the median LOS. The difference of both the values is mentioned as plus value against the factor that is larger or minus value against the factor that is smaller than the median LOS. Hence, we conclude that the category with (+value) stays (+value) days longer than the median LOS of that gestation group.

| **Table S2:** Models’ comparison | | | | | | |
| --- | --- | --- | --- | --- | --- | --- |
|  | **Fit statistics** | | **Fit for conditional distribution** | **RMSE** | | **R^2^** |
| **Model / Response Variable** | **AIC** | **BIC** | **Pearson Chi-sq/ DF** | **Construction Dataset** | **Testing Dataset** | **Construction Dataset** |
| 1 / LMM: raw scale | 4494.6 | 4664.7 | 1.03 | 4.91 | 5.57 | 0.59 |
| **2 / LMM: log scale** | **923.2** | **1093.3** | **1.03** | **5.15** | **5.62** | **0.69** |
| 3 / LMM: sqrt scale | 1519.6 | 1689.7 | 1.04 | 4.89 | 5.48 | 0.66 |
| 4 / LMM: (LOS)^(-0.3)^ | -1651.8 | -1481.6 | 1.04 | 5.89 | 6.21 | 0.68 |
| 5 / GLM: family (Poisson), link (Log) | 3988.1 | 4153.6 | 1.05 | 4.87 | 9.44 | 0.6 |
| 6 / GLM: family (NB), link(Log) | 3670 | 3840.2 | 1.05 | 5.07 | 9.43 | 0.31 |
| 7 / GLM: family (gamma), link(Log) | 3464.9 | 3635 | 1.05 | 5.24 | 9.43 | 0.16 |

| \| **Table S3:** Baseline characteristics of the validation population \| \| \| \| \| \| --- \| --- \| --- \| --- \| --- \| \| **Gestation (weeks)** \| \| \| \| \| \| **Characteristics** \| **26-32**  **(n = 24)** \| **32-34**  **(n= 61)** \| **34-37**  **(n= 62)** \| **>37**  **(n= 64)** \| \| **Perinatal Factors**  Multiple Pregnancy  Antenatal  Infections  Maternal disease  Steroids  Risk factors  Umbilical Doppler  Abnormal  Normal  **Birth details**  Caesarean section  Need for PPV  Apgar 5 min, < 5  Gender, male  Inborn  Gestation, weeks^*^  Birth weight, grams^*^  **Clinical diagnosis**  Respiratory Distress  RDS  TTNB  MAS  Need for MV^#^  Pneumothorax  PPHN  Sepsis  NNH^$^  Asphyxia  **Nutrition**^‡^  Energy deviation, kcal/kg  IV^th^ quartile  Remaining  Protein deviation,gram/kg  IV^th^ quartile  Remaining  **Medication**  Medication received  No deviation  Deviation days^  Medication Not Required \| 11 (45.8)  2 (8.3)  6 (25)  13 (54.1)  13 (54.1)  0  22 (91.6)  21 (87.5)  4 (16.6)  0  18 (75)  19 (79.1)  30.4 (1.7)  1446.5 (412)  15 (62.5)  2 (8.3)  0  1 (4.1)  0  0  7 (29.1)  11 (45.8)  0  248.9 (99)  115.5 (52.6)  7.5 (1.6)  3.8 (2.9)  16 (66.7)  8 (33.3)  2 (5.5)  8 (33.3) \| 40 (65.5)  1 (1.6)  31 (50.8)  41 (67.2)  11 (18)  9 (14.7)  36 (59)  52 (85.2)  3 (4.9)  0  31 (50.8)  39 (63.9)  33.1 (0.6)  1806.8 (288.7)  12 (19.6)  22 (36.0)  0  2 (3.2)  0  0  6 (9.8)  22 (36.0)  0  108.9 (131.6)  42.8 (25.6)  4.1 (0.4)  2.4 (0.8)  16 (26.2)  15 (24.5)  19 (0)  45 (73.8) \| 9 (14.5)  3 (4.8)  16 (25.8)  14 (22.5)  3 (4.8)  1 (1.6)  43 (69.3)  46 (74.1)  2 (3.2)  1 (1.6)  38 (61.2)  36 (58.1)  35.8 (0.9)  2456.4 (529.6)  10 (16.1)  9 (14.5)  3 (4.8)  3 (4.8)  0  0  5 (8.0)  29 (46.7)  1 (1.6)  103.2 (43.4)  40.7 (32.3)  3.3 (1.1)  0.65 (1.4)  11 (17.7)  8 (12.9)  4 (6.5)  51 (82.3) \| 0  0  8 (12.5)  3 (4.6)  1 (1.5)  0  33 (51.4)  39 (60.9)  3 (4.6)  0  47 (73.4)  30 (46.8)  38.7(1)  2917(393)  11 (17.1)  9 (14.0)  3 (4.6)  7 (10.9)  0  1 (1.5)  9 (14.0)  35 (54.6)  4 (6.2)  122.9 (42)  0 (38.4)  3.4 (1.6)  0 (1.1)  18 (28.1)  13 (20.3)  9 (6)  46 (71.9) \|   *All values are stated as Count (percentage) unless stated otherwise,*Mean (SD), ^#^Mechanical ventilation and/or surfactant administration, ^$^Neonatal Hyperbilirubinemia (requiring Phototherapy), ^Median of deviation days (IQR of deviation days), ^‡^Values mentioned as: Median (IQR) corresponding to respective quartiles.*  *PPV: Positive pressure ventilation, RDS: Respiratory Distress, TTNB: Transient tachypnea of the newborn, MAS: Meconium aspiration syndrome, MV: Mechanical Ventilation, PPHN: Persistent pulmonary hypertension of the newborn, NNH: Neonatal hyperbilirubinemia*   \| **Table S4 (a):** Medicines dosage deviation statistics \| \| \| \| \| \| --- \| --- \| --- \| --- \| --- \| \| **Medicines** \| **26-32 weeks** \| **32-34 weeks** \| **34-37 weeks** \| **>37 weeks** \| \| Meropenem \| 230/276 \| 11/25 \| 75/85 \| 56/69 \| \| Caffeine citrate \| 85/1604 \| 7/353 \| 8/195 \| 0/1 \| \| Amikacin \| 29/332 \| 26/204 \| 17/174 \| 0/124 \| \| Colistin \| 26/148 \| 1/33 \| 12/89 \| 12/77 \| \| Piperacillin Tazobactam \| 0/329 \| 0/261 \| 0/278 \| 0/311 \| \| Cefotaxime \| 0/27 \| 0/9 \| 0/6 \| 2/15 \| \| Vancomycin \| 0/21 \| 17/19 \| 22/22 \| 22/29 \| \| Fluconazole \| 0/17 \| 0/16 \| 14/20 \| 3/40 \| \| Surfactant  DOPamine \| 0/10  0/8 \| 0/7  0/4 \| 0/15  0/16 \| 0/9  0/70 \| \| Amphotericin B Liposome \| 0/2 \| 0/1 \| 0/28 \| 1/24 \| \| *All values are mentioned as “Number of days having deviations/Number of total days”, medicines arranged in descending orders according to deviation days* \| \| \| \| \|   **Table S4 (b):** Medicine deviation data as per dosage | | | | |
| --- | --- | --- | --- | --- | --- | --- | --- | --- | --- | --- | --- | --- | --- | --- | --- | --- | --- | --- | --- | --- | --- | --- | --- | --- | --- | --- | --- | --- | --- | --- | --- | --- | --- | --- | --- | --- | --- | --- | --- | --- | --- | --- | --- | --- | --- | --- | --- | --- | --- | --- | --- | --- | --- | --- | --- | --- | --- | --- | --- | --- | --- | --- | --- | --- | --- | --- | --- | --- | --- | --- | --- | --- | --- | --- | --- | --- | --- | --- | --- | --- | --- | --- | --- | --- | --- | --- | --- | --- | --- |
| **Medicines** | **26-32 weeks** | **32-34 weeks** | **34-37 weeks** | **>37 weeks** |
| Acetaminophen | 0/0 | 0/0 | 3/9 | 7/9 |
| Amikacin | 29/332 | 26/204 | 17/174 | 0/124 |
| Amphotericin B | 0/47 | 0/0 | 0/0 | 3/17 |
| Amphotericin B Liposome | 0/2 | 0/1 | 0/28 | 1/24 |
| Azithromycin | 0/13 | 0/0 | 0/0 | 0/1 |
| Caffeine citrate | 85/1604 | 7/353 | 8/195 | 0/1 |
| Cefotaxime | 0/27 | 0/9 | 0/6 | 2/15 |
| Ceftriaxone | 0/0 | 0/0 | 0/0 | 5/7 |
| Ciprofloxacin | 2/45 | 0/0 | 0/0 | 0/0 |
| Colistin | 26/148 | 1/33 | 12/89 | 12/77 |
| Dobutamine | 0/0 | 0/0 | 0/0 | 0/1 |
| Dopamine | 0/8 | 0/4 | 0/16 | 0/70 |
| Epinephrine | 0/0 | 0/0 | 0/0 | 0/2 |
| Fluconazole | 0/17 | 0/16 | 14/20 | 3/40 |
| Hydrocortisone | 0/0 | 0/0 | 0/0 | 3/4 |
| Ibuprofen | 0 | 0 | 0/6 | 0 |
| Levetiracetam | 0 | 0/1 | 0/21 | 18/76 |
| Linezolid | 13/62 | 0 | 0 | 0 |
| Meropenem | 230/276 | 11/25 | 75/85 | 56/69 |
| Morphine | 0 | 0 | 1/1 | 5/5 |
| Octreotide | 0 | 0 | 0 | 7/7 |
| Phenobarbital | 0 | 0 | 0 | 9/25 |
| Phenytoin | 0 | 0 | 0 | 0/1 |
| Piperacillin Tazobactam | 0/329 | 0/261 | 0/278 | 0/311 |
| Ranitidine | 29/29 | 0 | 0 | 0 |
| Sildenafil | 0 | 0 | 0 | 2/2 |
| Surfactant  Vancomycin | 0/10  0/21 | 0/7  17/19 | 0/15  22/22 | 0/9  22/29 |
| All values are mentioned as “Number of days having deviations/Number of total days” | | | | |

| **Table S4 (c):** Medicines and deviation days frequency statistics (the medicines are listed in rank order of deviations) | | | | |
| --- | --- | --- | --- | --- |
| **Medicines** | **26-32 weeks** | **32-34 weeks** | **34-37 weeks** | **>37 weeks** |
| Meropenem | 104/275 | 2/25 | 23/85 | 8/68 |
| Amikacin | 34/332 | 26/204 | 8/174 | 0/114 |
| Colistin | 28/148 | 15/33 | 17/89 | 7/76 |
| Piperacillin Tazobactam | 10/325 | 3/255 | 19/278 | 51/295 |
| Caffeine citrate | 3/1458 | 0/315 | 8/178 | 0/1 |
| Amphotericin B | 0/47 | 0/0 | 0/0 | 0/17 |
| Cefotaxime | 0/27 | 0/9 | 0/6 | 5/15 |
| Vancomycin | 0/20 | 0/19 | 0/22 | 6/29 |
| Fluconazole  Amphotericin B  (Liposome) | 0/17  0/2 | 2/16  0/1 | 6/20  0/28 | 16/39  0/24 |
| *All values are mentioned as “Number of orders having deviations/Number of total orders”* | | | | |

| **Table S4 (d):** Medicine deviation data as per frequency | | | | |
| --- | --- | --- | --- | --- |
| **Medicines** | **26-32 weeks** | **32-34 weeks** | **34-37 weeks** | **>37 weeks** |
| Acetaminophen | 0 | 0 | 6/9 | 2/9 |
| Amikacin | 34/332 | 26/204 | 8/174 | 0/114 |
| Amphotericin B | 0/47 | 0 | 0 | 0/17 |
| Amphotericin B Liposome | 0/2 | 0/1 | 0/28 | 0/24 |
| Azithromycin | 0/13 | 0 | 0 | 0/1 |
| Caffeine citrate | 3/1458 | 0/315 | 8/178 | 0/1 |
| Cefotaxime | 0/27 | 0/9 | 0/6 | 5/15 |
| Ceftriaxone | 0 | 0 | 0 | 5/7 |
| Ciprofloxacin | 0/45 | 0 | 0 | 0 |
| Colistin | 28/148 | 15/33 | 17/89 | 7/76 |
| Fluconazole | 0/17 | 2/16 | 6/20 | 16/39 |
| Hydrocortisone | 0 | 0 | 0 | 3 /4 |
| Ibuprofen Lysine | 0 | 0 | 0/6 | 0 |
| Levetiracetam | 0 | 1/1 | 19/19 | 56/61 |
| Linezolid | 30/62 | 0 | 0 | 0 |
| Meropenem | 104/275 | 2/25 | 23/85 | 8/68 |
| Octreotide | 0 | 0 | 0 | 7/7 |
| Phenobarbital | 0 | 0 | 0 | 0/9 |
| Phenytoin | 0 | 0 | 0 | 1/1 |
| Piperacillin Tazobactam | 10/325 | 3/255 | 19/278 | 51/295 |
| Ranitidine | 29/29 | 0 | 0 | 0 |
| Surfactant  Vancomycin | 0/10  0/20 | 0/7  0/19 | 0/15  0/22 | 0/9  6/29 |
| All values are mentioned as “Number of days having deviations/Number of total days” | | | | |

| **Table S5:** Significant independent variables affecting LOS across various categories (full table) | | | | |
| --- | --- | --- | --- | --- |
|  | **26-32weeks**  **(n=85)** | **32–34 weeks**  **(n=211)** | **34-37 weeks**  **(n=267)** | **37 weeks**  **(n=273)** |
| Median LOS (IQR) | 25 (16) | 6 (7) | 4 (3.5) | 4 (3) |
| **Perinatal factors** | | | | |
| Gestational Category (1^st^ Quartile) | +19 | +4 |  | 0 |
| Gestational Category (Remaining Quartile) | -3 | -1 |  | 0 |
|  |  |  |  |  |
| Male |  | +1 | +1 |  |
| Female |  | 0 | 0 |  |
| Inborn |  |  |  |  |
| Outborn |  |  |  |  |
| Caesarean delivery |  |  | +1 |  |
| Normal delivery |  |  | 0 |  |
| Single birth |  |  |  |  |
| Multiple births |  |  |  |  |
| Need for resuscitation Initial steps |  |  |  |  |
| Need for resuscitation O2 |  |  |  |  |
| Need for resuscitation PPV |  |  |  |  |
| No need for resuscitation |  |  |  |  |
| Need for resuscitation unknown |  |  |  |  |
| APGAR <=5 (1 or 5 mins) |  |  |  |  |
| APGAR >5 (1 or 5 mins) |  |  |  |  |
| Umbilical doppler normal |  |  |  |  |
| Umbilical doppler abnormal |  |  |  |  |
| Umbilical doppler unknown |  |  |  |  |
| **Antenatal and perinatal factors** | | | | |
| Antenatal steroids given |  |  |  |  |
| Antenatal steroids not given |  |  |  |  |
| Antenatal diseases present |  |  |  |  |
| No antenatal diseases |  |  |  |  |
| Antenatal infections present |  |  |  |  |
| Antenatal infections absent |  |  |  |  |
| Antenatal risk factors present |  |  |  |  |
| Antenatal risk factors absent |  |  |  |  |
| **Nutrition deviation** | | | | |
| Energy (1^st^, 2^nd^ and 3^rd^ quartiles) | -7 | -1 |  |  |
| Energy (4^th^ quartile) | +6 | +1 |  |  |
| Protein (1^st^, 2^nd^ and 3^rd^ quartiles) |  |  | -1 | -1 |
| Protein (4^th^ quartile) |  |  | +1 | +1 |
| **Medication** | | | | |
| Medication Not Required |  | 0 | 0 | -1 |
| **Medication deviation** | | | | |
| Dose deviation |  | +7 | +7 | +4 |
| Dose No deviation |  | +3 | +3 | +2 |
| **Clinical diagnosis** | | | | |
| RDS present |  |  |  | +2 |
| RDS absent  RDS TTNB present  RDS TTNB absent |  |  |  | -1  +1  0 |
| Severe RDS (Invasive ventilation) present |  |  | +5 | +6 |
| Severe RDS (Invasive ventilation) absent |  |  | 0 | 0 |
| Sepsis present |  | +9 | +7 | +7 |
| Sepsis absent |  | 0 | 0 | 0 |
| NNH present |  | +3 | +1 |  |
| NNH absent |  | 0 | 0 |  |
| Asphyxia present |  |  |  |  |
| Asphyxia absent |  |  |  |  |
| Pneumothorax present |  |  |  |  |
| Pneumothorax absent |  |  |  |  |
| PPHN present |  |  |  |  |
| PPHN absent |  |  |  |  |

| **Table S6 (a):** Energy deviation - Variation in LOS across gestation categories | | | | | | | | |
| --- | --- | --- | --- | --- | --- | --- | --- | --- |
| **ENERGY** | | | | | | | | |
| Min/Max from formula | 20 | 480 | 0 | 291 | 0 | 249 | 0 | 264 |
| Gestation | 26-32 | | 32-34 | | 34-37 | | >37 | |
|  | LOS | Value | LOS | Value | LOS | Value | LOS | Value |
| 1^st^ quartile | 17.5 | 99 | 5 | 22.7 | 3 | 0 | 3 | 0 |
| 2^nd^ quartile | 23 | 146.2 | 5 | 47.5 | 4 | 41 | 3 | 39.3 |
| 3^rd^ quartile | 31 | 181.6 | 7 | 71.7 | 4 | 64 | 4 | 77.6 |
| 4^th^ quartile | 41 | 247.7 | 13 | 128.8 | 7 | 120.4 | 7 | 132.8 |

| **Table S6 (b):** Protein deviation - Variation in LOS across gestation categories | | | | | | | | |
| --- | --- | --- | --- | --- | --- | --- | --- | --- |
| **PROTEIN** | | | | | | | | |
| Min/Max from formula | 1 | 9 | 0 | 8 | 0 | 11 | 0 | 9 |
| Gestation | 26-32 | | 32-34 | | 34-37 | | >37 | |
|  | LOS | Value | LOS | Value | LOS | Value | LOS | Value |
| 1^st^ quartile | 22.5 | 2.5 | 4 | 1.7 | 3 | 1 | 3 | 0.3 |
| 2^nd^ quartile | 23 | 4.4 | 6 | 2.5 | 3 | 2 | 3 | 1.5 |
| 3^rd^ quartile | 25 | 5.6 | 6 | 3.5 | 4 | 3.4 | 4 | 2.6 |
| 4^th^ quartile | 32 | 7.6 | 11 | 5.1 | 8 | 4.7 | 6 | 4.1 |

| **Table S6 (c):** Vitamin A deviation – Variation in LOS across gestation categories | | | | | | | | |
| --- | --- | --- | --- | --- | --- | --- | --- | --- |
| **Vitamin A** | | | | | | | | |
| Min/Max from formula | 0 | 11654 | 0 | 8610 | 0 | 6672 | 0 | 9173 |
| Gestation | 26-32 | | 32-34 | | 34-37 | | >37 | |
|  | LOS | Value | LOS | Value | LOS | Value | LOS | Value |
| 1^st^ quartile | 22 | 952.2 | 4 | 560.5 | 3 | 0 | 3 | 0 |
| 2^nd^ quartile | 29 | 1903.1 | 6 | 1018.5 | 3 | 902.7 | 3 | 1056.5 |
| 3^rd^ quartile | 31 | 3164.6 | 6 | 1729 | 3 | 1539.6 | 3 | 1566.5 |
| 4^th^ quartile | 28 | 4890.2 | 11 | 2938.1 | 6 | 2473.1 | 5 | 2212.7 |

| **Table S6 (d):** Vitamin D deviation - Variation in LOS across gestation categories | | | | | | | | |
| --- | --- | --- | --- | --- | --- | --- | --- | --- |
| **Vitamin D** | | | | | | | | |
| Min/Max from formula | 0 | 4707 | 0 | 2770 | 0 | 2045 | 0 | 1691 |
| Gestation | 26-32 | | 32-34 | | 34-37 | | >37 | |
|  | LOS | Value | LOS | Value | LOS | Value | LOS | Value |
| 1^st^ quartile | 22 | 410.3 | 4 | 187.9 | 4 | 0 | 4 | 0 |
| 2^nd^ quartile | 22 | 965.5 | 5 | 309.6 | 3 | 276 | 3 | 271.2 |
| 3^rd^ quartile | 22 | 2052.8 | 6 | 555.9 | 3 | 397 | 3 | 390.3 |
| 4^th^ quartile | 41 | 3151.8 | 13 | 1169 | 7 | 632.7 | 5 | 548.5 |

| **Table S6 (e):** Calcium deviation - Variation in LOS across gestation categories | | | | | | | | |
| --- | --- | --- | --- | --- | --- | --- | --- | --- |
| **Calcium** | | | | | | | | |
| Min/Max from formula | 27 | 1470 | 0 | 1519 | 0 | 1069 | 0 | 719 |
| Gestation | 26-32 | | 32-34 | | 34-37 | | >37 | |
|  | LOS | Value | LOS | Value | LOS | Value | LOS | Value |
| 1^st^ quartile | 22.5 | 255.7 | 4 | 69 | 3 | 0 | 3 | 0 |
| 2^nd^ quartile | 17 | 408.9 | 5 | 145.8 | 3 | 139.7 | 3 | 130.4 |
| 3^rd^ quartile | 29 | 572.4 | 7 | 280.7 | 4 | 216.7 | 4 | 212 |
| 4^th^ quartile | 30 | 783.9 | 13 | 405.7 | 7 | 355.2 | 5 | 288.9 |

| **Table S6 (f):** Phosphorus deviation - Variation in LOS across gestation categories | | | | | | | | |
| --- | --- | --- | --- | --- | --- | --- | --- | --- |
| **Phosphorus** | | | | | | | | |
| Min/Max from formula | 27 | 765 | 0 | 542 | 0 | 448 | 0 | 310 |
| Gestation | 26-32 | | 32-34 | | 34-37 | | >37 | |
|  | LOS | Value | LOS | Value | LOS | Value | LOS | Value |
| 1^st^ quartile | 21.5 | 143.2 | 4 | 28.4 | 3 | 0 | 3 | 0 |
| 2^nd^ quartile | 20 | 197.4 | 5 | 66.8 | 3 | 60.7 | 3 | 56.5 |
| 3^rd^ quartile | 31 | 270.2 | 6 | 135.5 | 4 | 100.4 | 4 | 93.1 |
| 4^th^ quartile | 32 | 375.4 | 13 | 186.4 | 7 | 155.9 | 7 | 129.8 |

| **Table S6 (g):** Iron deviation - Variation in LOS across gestation categories | | | | | | | | |
| --- | --- | --- | --- | --- | --- | --- | --- | --- |
| **IRON** | | | | | | | | |
| Min/Max from formula | 2 | 33 | 0 | 38 | 0 | 35 | 0 | 35 |
| Gestation | 26-32 | | 32-34 | | 34-37 | | >37 | |
|  | LOS | Value | LOS | Value | LOS | Value | LOS | Value |
| 1^st^ quartile | 19.5 | 8.3 | 4 | 4.3 | 4 | 0 | 4 | 0 |
| 2^nd^ quartile | 24 | 13.4 | 6 | 6.6 | 3 | 5.8 | 3 | 5.6 |
| 3^rd^ quartile | 30 | 16.7 | 6 | 9.2 | 3 | 8.2 | 3 | 8.2 |
| 4^th^ quartile | 36 | 24.3 | 11 | 14.2 | 7 | 12.7 | 5 | 11.4 |
